# Discovery and Analysis of Evolutionarily Conserved Intronic Splicing Regulatory Elements

**DOI:** 10.1371/journal.pgen.0030085

**Published:** 2007-05-25

**Authors:** Gene W Yeo, Eric L. Van Nostrand, Tiffany Y Liang

**Affiliations:** 1 Crick-Jacobs Center for Theoretical and Computational Biology, Salk Institute, La Jolla, California, United States of America; 2 Laboratory of Genetics, Salk Institute, La Jolla, California, United States of America; RIKEN Genomic Sciences Center, Japan

## Abstract

Knowledge of the functional *cis-*regulatory elements that regulate constitutive and alternative pre-mRNA splicing is fundamental for biology and medicine. Here we undertook a genome-wide comparative genomics approach using available mammalian genomes to identify conserved intronic splicing regulatory elements (ISREs). Our approach yielded 314 ISREs, and insertions of ~70 ISREs between competing splice sites demonstrated that 84% of ISREs altered 5′ and 94% altered 3′ splice site choice in human cells. Consistent with our experiments, comparisons of ISREs to known splicing regulatory elements revealed that 40%–45% of ISREs might have dual roles as exonic splicing silencers. Supporting a role for ISREs in alternative splicing, we found that 30%–50% of ISREs were enriched near alternatively spliced (AS) exons, and included almost all known binding sites of tissue-specific alternative splicing factors. Further, we observed that genes harboring ISRE-proximal exons have biases for tissue expression and molecular functions that are ISRE-specific. Finally, we discovered that for *Nova1, neuronal PTB, hnRNP C,* and *FOX1,* the most frequently occurring ISRE proximal to an alternative conserved exon in the splicing factor strongly resembled its own known RNA binding site, suggesting a novel application of ISRE density and the propensity for splicing factors to auto-regulate to associate RNA binding sites to splicing factors. Our results demonstrate that ISREs are crucial building blocks in understanding general and tissue-specific AS regulation and the biological pathways and functions regulated by these AS events.

## Author Summary

During RNA splicing, sequences (introns) in a pre-mRNA are excised and discarded, and the remaining sequences (exons) are joined to form the mature RNA. Splicing is regulated not only by the binding of the basic splicing machinery to splice sites located at the exon–intron boundaries, but also by the combined effects of various other splicing factors that bind to a multitude of sequence elements located both in the exons as well as the flanking introns. Instances of alternative splicing, where usage of splice site(s) is incomplete or different between tissues, cell types, or lineages, can be created by the interaction of sequence elements and tissue, cell type, and stage-specific splicing factors. To better understand constitutive and alternative pre-mRNA splicing, the authors describe a comparative genomics approach, using available mammalian genomes, to systematically identify splicing regulatory elements located in the introns proximal to exons. A quarter of the elements were tested experimentally, and most of them altered splicing in human cells. The authors also showed that that the intronic elements are close to tissue-specific alternative exons and are more likely to be located in specific positions in the introns, suggestive of potential regulatory function. These elements are also frequently found in tissue-specific genes, suggesting a coupling between expression and alternative splicing of these genes. Finally, the authors propose a strategy using the elements to identify the binding sites of several splicing factors.

## Introduction

Considering that the human genome contains upwards of 20,000 genes with an average of eight to ten exons per gene, it is remarkable that the RNA splicing machinery faithfully distinguishes exons from intronic sequences that are 100- to 1,000-fold larger in size. Many studies show that the fidelity of splicing relies on cooperative interaction between the splicesomal complex and splicing *trans* factors (reviewed in [1]). Simple models of exon recognition depict *trans* factors binding to splicing regulatory elements (SREs) in *cis* that are in intronic regions proximal to the exon, or within the exon itself, resulting in either increased exon usage (splicing enhancers), or decreased splice site recognition (splicing silencers) [2–4]. In addition to regulating constitutive splicing (e.g., where a gene has only one isoform), SREs are also important in regulating tissue-specific and developmentally regulated alternative splicing events [2–4]. Evidence that as many as 75% of human genes undergo alternative splicing, whereby multiple isoforms are derived from the same genic location, underscores the complexity of RNA splicing regulation [5]. Last, a study restricted to analysis of the canonical splice signals reported that 15% of point mutations disrupted splicing [6], a likely gross underestimate of the impact of splicing on human disease. A complete catalog of SREs is necessary to improve our understanding of the mechanisms controlling splicing, for it will enable rapid diagnosis and treatment of splicing-associated diseases [7].

Recent work in the splicing field has focused on establishing a “splicing code” or a “parts list” of SREs [8]. Both computational approaches and experimental screens have made progress in identifying exonic splicing enhancers [9,10] and exonic splicing silencers [10,11], of which several also modulate splice site choice [12]. Recent computational results have also suggested that intronic regions flanking constitutive exons contain potential splicing regulatory sequences [13–15]. With the availability of multiple genomes, several comparative genomics approaches have been applied to identify exonic regulatory elements that affect alternative and constitutive splicing in mammals [16], and intronic regulatory elements that are proximal to alternative exons in worms [17]. From another direction, expressed sequence tags (ESTs) and splicing-sensitive microarrays have been useful in discovering cell type and tissue-specific included or skipped alternative spliced exons [5,18–22]. Sequence analysis can be used to derive tissue-specific SREs in the exonic regions or in flanking introns [18].

By exploiting evolutionary conservation of functional elements, comparative genomics has proven effective in identifying known and novel regulatory elements in noncoding regions of mammalian genomes [23]. A recent study identified regulatory motifs in the promoters and 3′ untranslated regions of mammalian genes [24]. However, the authors surveyed the last two introns of genes but overlooked the intronic regions proximal to all exons [24]. Computational analyses of alternative exons that are evolutionarily conserved in human and mouse indicated that up to 150 bases of intronic regions flanking alternative exons in both human and mouse have significantly higher conservation than regions flanking constitutive exons [25–27], suggesting that they have a regulatory function.

Our goal is to systematically identify intronic splicing regulatory elements (ISREs) that occurred frequently in the intronic regions proximal to exons and that are conserved across mammalian evolution. We subjected candidate ISREs to a battery of computational analysis to discover their biological functions, and experimentally tested almost a quarter of predicted ISREs by splicing reporter assays, with a validation rate of 84% for the downstream ISREs and 94% for the upstream ISREs. While previous studies restricted their analyses to exons that are predicted to be alternatively spliced (AS) by alignments of ESTs, or to exons identified from splicing-sensitive microarray experiments [17–19,27], we believed a whole-genome comparative genomics approach would yield an unbiased, comprehensive set of evolutionarily important SREs.

## Results/Discussion

### Discovery of Conserved ISREs

Here, we describe our steps to identify conserved ISREs. First, we generated a set of conserved exons and intronic regions flanking the exons. A total of ~17,870 isoform clusters from the human RefSeq database were obtained from the University of California Santa Cruz (UCSC) genome browser. Next, ~2.7 million spliced human ESTs were aligned to each cluster, and exon boundaries were extended to generate a composite exon–intron structure for the cluster. The mammalian exon dataset was generated by extracting conserved *(Homo sapiens, Canis familiaris, Rattus norvegicus,* and *Mus musculus)* internal exons from genome-wide multiple alignments [28,29]. First exons were excluded, as the first introns of protein-coding genes are known to contain transcription factor–binding sites, and may obscure the search for SREs. In order to capture a sufficiently large region while avoiding other functional elements, such as microRNAs and snoRNAs, 400 bases of flanking intronic sequence proximal to both splice sites upstream and downstream of exons were targeted for analysis. Approximately 161,730 exons comprising 24.2 Mb of exonic sequence and ~129 Mb of intronic sequence were generated from the human genome, not including the orthologous sequence segments from other mammalian genomes.

Second, we counted the number of words (five to seven bases in length) that were completely conserved in mammals and the total number of occurrences in human, in the intronic regions 400 bases downstream and 400 bases upstream of all internal exons, respectively. We designated a χ^2^ value to each word, which measured the relative conservation rate of each word compared to all other words of the same length. Next, we retained words that had significantly high χ^2^ values (*p* < 0.001, using a Bonferroni correction for multiple hypothesis testing). For example, the lowest scoring word of length 6 retained was GAAACT with a conservation rate ~1.3-fold higher than background. GAAACT had a χ^2^ score of 20.8, with an associated *p* value of approximately 5 × 10^−6^. This implied that out of 4,096 possible words of six bases in length, we did not expect to see any words with an equal or higher score by chance. In order to avoid identifying transcription factor–binding sites, we repeated our procedure to extract significantly conserved words in a previously published dataset of promoter regions aligned across the same mammalian genomes [24]. A total of 583 downstream and 630 upstream words from our intronic screen were significantly enriched in promoters, and were discarded from further analysis. In all, 8.69%, 3%, and ~1% of downstream words; and 9.3%, 3.3%, and 1.2% of upstream words of five, six, and seven bases, respectively, scored significantly. In addition, approximately 25% of the significantly enriched words were common between upstream and downstream words.

Third, we developed a score-based clustering procedure to group the words into motif families, and defined each family as an ISRE. Our clustering procedure comprised four steps (see Figure S1). In the “backward” step we compared all longer words (e.g., seven bases) to shorter words (e.g., six bases). A word was defined as a “parent” of another word (“child”) if it was a strict substring of the longer word and had a higher χ^2^ score. In the “forward” step, we compared all shorter words to longer words, and defined a word as a child of a longer word if it was a substring of the longer word and had a lower χ^2^ score, and if it was already not a parent from the backward step. Next, two families were “married” if they shared more than half of their children. The parent with the higher χ^2^ score was designated the new parent; the other parent became a child in the family. Two families were combined if parents had five bases in common, and if all children of the lower scoring parent had five bases in common with the higher-scoring parent. Again, the parent with the higher χ^2^ score was designated the new parent. If a child had more than one final parent it was uniquely associated with the highest-scoring parent. Our clustering strategy had several features. First, it ensured that the parent and thus representative of a motif family had the highest conservation rate in the family. Second, words were grouped by similarity to an actual word(s), which existed in biological sequence and can be experimentally verified, and not to a statistical average of the words, such as in positional weight matrices (PWMs). Using a weight-matrix approach has the limitation that high-scoring sequences with a weight matrix may never occur in reality. In this paper we used a weight-matrix representation for visualization purposes, but as much as possible retained groups of words for an ISRE during enumeration and experimental validation. An important caveat in our analysis is that on occasion we had more than one cluster with motifs that might have been grouped as one cluster by other clustering methods. We preferred to rely on future experimental means to associate the clusters with a splicing factor, rather than to tease apart different splicing factors binding to the same cluster. Alternatively, each element could be treated as a separate ISRE, but for current practical purposes, we believed our clustering procedure was a reasonable intermediate approach.

Our strategy generated a final set of 158 downstream (D1–D158) and 156 upstream (U1–U156) mammalian ISREs. The median number of words comprising each ISRE is five; the smallest ISRE family consisted of two words, and the largest family consisted of 32 words (Table S1 contains all downstream ISREs, and Table S2 contains all upstream ISREs). Overall, 22% of the ~400 base intronic regions were perfectly conserved across mammals, and the ISREs comprised 5.21% (downstream) and 5.5% (upstream) of the conserved regions. This implied that 1.1% to 1.2% of exon-proximal intronic regions were evolutionarily preserved ISREs.

### ISREs Resemble Known SREs

Here, we compare ISREs to known SREs. First, we used positional distribution biases (described in Methods and Materials) to identify ISREs that resembled canonical splicing signals, namely 5′ splice sites (5′ss), 3′ splice sites (3′ss), and branch signals, which we expected to be highly conserved (Figure S2). A total of 17% (27 of 158) of downstream ISREs resembled the major spliceosomal 5′ss consensus (GT[AG]AGT), and 1.3% (two of 158) downstream ISREs resembled the 5′ss of the U12 minor spliceosome ([A|G]TATCCT) (see Protocol S1 for a discussion of conserved U12-type introns). Similarly, 1.3% (two of 156) upstream ISREs resembled the 3′ss consensus (TTTCAG). The branch point signal was detected as well (Table S2), peaking at ~15–40 bp from the 3′ss (Figure S2) [30].

**Figure 1:**
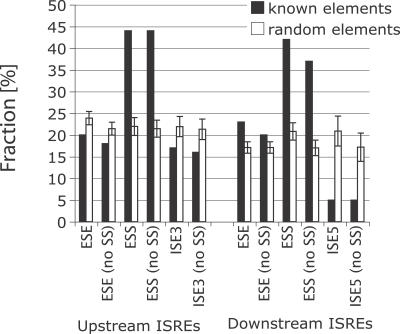
Overlap of ISREs with Known Classes of SREs, Namely ESE, ESS, ISE5, and ISE3 Hexamers Two sequences are defined as overlapping if one is a subsequence of the other. The fraction of ISREs that overlap with a class of hexamers is indicated in filled black bars. The white bars represent the mean fraction of 1,000 comparisons of ISREs with sets of random hexamers of equal size as the class (error bars depict one standard deviation). Refer to Protocol S1 on details of the compilation of known splicing regulatory sequences from multiple sources. “No SS” refers to using ISREs without splice-site like sequences.

Next, we asked if ISREs overlapped with previously published sets of SREs, other than canonical splice signals. Three categories of k-mers representing exonic splicing enhancers (ESEs) [9,10], exonic splicing silencers (ESSs) [10,12], and intronic splicing enhancers (ISEs) [13] were analyzed [31] (see Protocol S1 for details on the compilation of the sets). We observed that a significantly larger fraction of ESS sequences matched upstream ISREs (Figure 1; 44% of 386 ESSs, compared with 21.5% ± 2% for random sequences; *p* < 0.001), and a similar fraction matched downstream ISREs (37% of 386 sequences, compared with 17% ± 1.8% for random sequences; *p <* 0.001). Alternative splicing factors such as *Nova1* or *Nova2* inhibited exon inclusion when bound to *Nova* binding sites in the regulated exon, but modulated exon inclusion/exclusion differently depending on whether the binding sites are located in the upstream or downstream intron [32,33]. Therefore, one can assign more than one role to the *Nova* binding site, as being both an ESS and an intronic regulatory sequence. Indeed, the *Nova1/Nova2* binding site was predicted as an ISRE, and also was present as an ESS [11]. Our results suggest that in addition to being intronic modulators of splicing, at least 53% (84) of upstream ISREs and 42% (67) of downstream ISREs might have dual roles as ESSs (Tables S1 and S2 indicate the ISREs and their overlap with known elements).

Next, we compared ISREs with ISEs, which were divided into those that were downstream of 5′ss (ISE5s) and those that were upstream of 3′ss (ISE3s) [13]. We observed a significant but small depletion of upstream ISREs in ISE3s: 17% of ISE3s matched upstream ISREs, compared with 21.9% ± 2.3% for random sequences (*p* < 0.05). Strikingly, only 5% of ISE5s matched downstream ISREs, compared with 20.8% ± 3.5% (*p* < 0.001). Similar results were observed when both ISE5s and ISE3s were combined: we observed a small depletion of upstream ISREs (*p* < 0.05, unpublished data) and a more significant depletion of downstream ISREs in all lSEs (*p* < 0.001, unpublished data) in the combined set. ISEs were originally computationally identified as sequence elements that were more frequent in introns versus exons, and more frequent in introns proximal to weak splice sites versus introns proximal to strong splice sites flanking constitutive exons [34]. In addition, ISEs in mammals were more frequently proximal to splice sites in short introns (<125 bases) relative to long introns (>1,000 bases) [13,34]. However, as alternative exons tend to be flanked by significantly longer introns relative to constitutive exons [25–27], these results suggest that ISREs are unlikely to have a role in regulating constitutive exons, but that they are involved with the regulation of alternative exons. In addition, the 3-fold lower depletion of ISE5 sequences in ISREs compared with ISE3 sequences suggested that splice site–specific regulatory differences between alternative versus constitutive splicing may exist at the 5′ss. Finally, comparing the ISREs to ESEs showed small differences (Figure 1). We concluded that while a fraction of the identified ISREs resembled ESSs, most likely do not function as known ESEs or ISEs, and were likely to represent novel SREs.

### ISREs Inhibit Intron-Proximal 5′ss and 3′ss

In order to test whether ISREs generally altered splicing in human cells, we used previously published splicing reporters [12]. Wang and colleagues observed that ESSs identified from an experimental screen altered splice site choice by inhibiting the intron-proximal splice site when inserted between competing 5′ss, with similar but weaker effects when inserted between competing 3′ss [11,12]. The authors also showed that five out of ten ESEs enhanced use of the intron-proximal 5′ss, but had no appreciable effect on splice site choice in the 3′ss construct [12].

**Figure 2:**
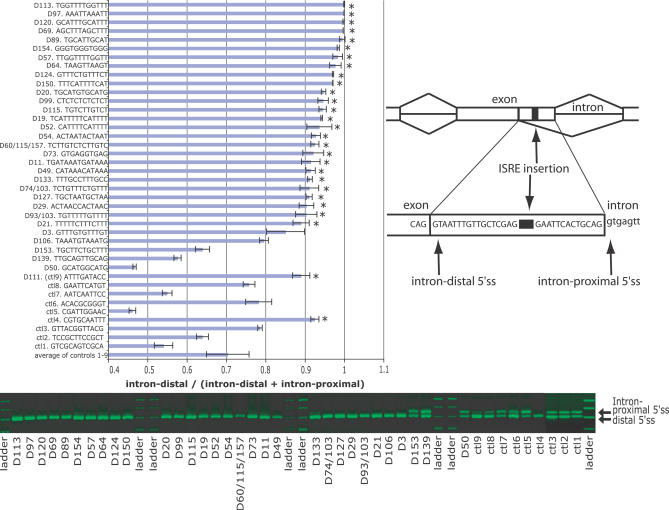
Downstream ISREs Alter Splice Site Choice The 5′ss reporter assay is depicted on the right. Boxes indicate exons, horizontal lines connecting the exons indicate introns; angled lines connecting boxes represent splicing choices. The nucleotide sequence between the intron-proximal and intron-distal splice sites (indicated by arrows) is depicted. ISREs indicated as a black bar are inserted in tandem duplicates between the competing 5′ss. Each ISRE-containing reporter was transfected in triplicate in human 293T cells. A representative PCR product for each ISRE-containing reporter is depicted on the etimidium bromide–stained agarose gel. The larger and shorter bands on the gel correspond to the intron-proximal and intron-distal isoforms, respectively (indicated by arrows on the right of the gel). Horizontal bars depict the mean of three replicates measuring the ratio between the intron-distal isoform to the sum of both isoforms. Error bars depict one standard deviation. ctl indicates individual control elements. Asterisks on the right of each bar indicate ISREs that significantly altered splicing relative to the averaged controls by a *t* test (*p* < 0.01). Please refer to Protocol S1 for details on quantification of the isoforms.

**Figure 3:**
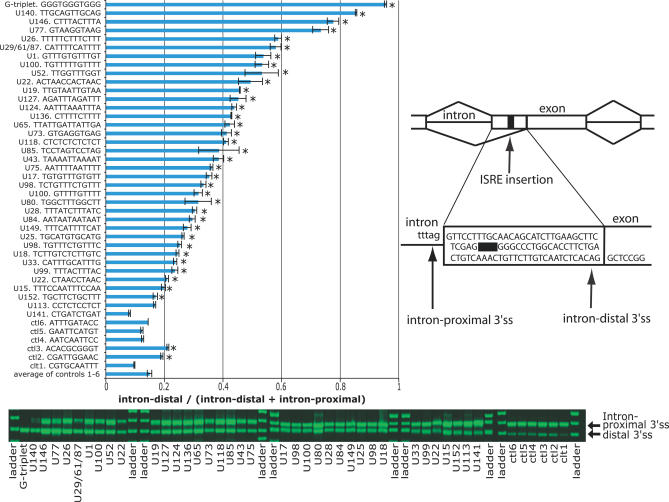
Upstream ISREs Alter Splice Site Choice The 3′ss reporter assay is depicted on the right. Boxes indicate exons, horizontal lines connecting the exons indicate introns; angled lines connecting boxes represent splicing choices. The nucleotide sequence between the intron-proximal and intron-distal splice sites (indicated by arrows) are depicted. ISREs indicated as a black bar are inserted in tandem duplicates between the competing 3′ss (see legend to Figure 2).

As the ISREs were significantly enriched with published ESS sequences, we reasoned that they would affect splice site choice in a similar manner. A total of 80 constructs, including previously published control elements [12], were generated and transfected in triplicate into human 293T cells. Several ISRE elements that resembled known elements, such as the *FOX1* binding site (UGCAUG) [35–38], the *PTB* binding site (CU-rich) [39], and the *Nova* site (YCAY) [40], were included to test their effects on splice site choice. We adopted an identical strategy to that of the previous authors for validating the ISREs [12] by generating most of the constructs with a tandem duplication of the parent sequence of the ISRE family. As many known alternative splicing factor binding sites, such as CU-rich sites for *PTB* [39] and YCAY sites for *Nova* [40], occurred in clusters, we believed that our insertions, which ranged from ten to 14 bases, represented accurate facsimiles of biological targets for the splicing factors. As in the previous study, the insertions did not overlap the proximal or distal 5′ss or 3′ss in the constructs (Figure 2 and 3). For these experiments, we measured the relative amount of the short (intron-distal) isoform as a fraction of both isoforms. An ISRE could affect splice site choice in two ways: (1) an ISRE could increase the preference for the intron-distal splice sites by suppressing the intron-proximal splice sites, resulting in a higher relative abundance of shorter isoform; or (2) an ISRE could increase the preference for the intron-proximal splice sites by suppressing the intron-distal splice sites, resulting in more of the longer isoform. For each inserted element, we asked if the ratios of the intron-distal isoform relative to the sum of both isoforms for an ISRE was significantly different from the average ratio of the controls by computing *t* test statistics, assuming that the elements were independent and the ratios were normally distributed. An ISRE was determined to suppress the intron-proximal 5′ss or 3′ss if the mean was significantly higher from the average controls at a *p*-value cutoff of 0.01.

We observed that 83% (26 of 31) of the tested downstream ISREs significantly suppressed the intron-proximal 5′ss in the 5′ss reporter, compared with the average ratio of the control elements (Figure 2). Negative control elements 1 to 3 were randomly selected sequences that did not match ISREs, ESSs, or ESEs; control elements 4 to 9 were the same as previously published controls, which were neither ESSs nor ESEs [12]. Interestingly, control 9 (ATTTGATACC in Figure 2) was revealed by our analysis to resemble a child of D111 (ATTTGAT). As predicted, this element also suppressed the intron-proximal 5′ss; thus, we did not consider it a valid control element, refining our validation rate to 84% (27 of 32). Of the remaining eight controls, only control element 4 suppressed the intron-proximal 5′ss. Therefore, we concluded that downstream ISREs were more likely to inhibit intron-proximal 5′ss (6- to 7-fold), relative to control sequences.

Downstream ISRE TGCATG (D20), which was similar to the *FOX1/2* binding site UGCAUG [35–38], suppressed the intron-proximal 5′ss. Interestingly, two other clusters which bore sequence similarity to D20, namely TGCAT (D89) and GCATG (D50), differed significantly in their ability to suppress the intron-proximal 5′ss when compared with TGCATG (D20)—D89 exhibited stronger suppression (*p <* 0.01), and D50 did not suppress the intron-proximal 5′ss (*p* < 0.001), which may reflect differences in the binding affinity of the splicing factor associated with the site. This observation suggested that our clustering strategy, although overtly conservative, had an advantage of allowing us to experimentally reveal ISRE differences in splice site regulation between closely related clusters. CU-rich motifs (D99) resembling the *PTB* binding site, TCATTT (D19) resembling the *Nova1* or *Nova2* binding sites, and a CTG-rich motif (D74, D103) resembling the MBNL [41] or CELF protein-binding sites also repressed the intron-proximal 5′ss [42,43]. ACTAAC (D29), which resembled the branch signal and was recently identified to be enriched downstream of muscle-inclusive alternative exons by Sugnet et al. [18], also suppressed the 5′ss, which is to our knowledge the first experimental evidence that this motif affected 5′ss selection in human cells.

We observed that 94% (34 of 36) of the upstream ISREs significantly suppressed the intron-proximal 3′ss in the 3′ss reporter, compared with the average ratio of the controls (Figure 3). Negative control elements 1 to 6 were the same as previously published controls [12]. As an additional positive control, we included a motif (GGGTGGGTGGG) that was not present in our upstream ISREs but which had sequences that resembled G triplets, a known intronic splicing enhancing sequence [34]. This motif exhibited the strongest suppression of the intron-proximal splice site. TTGCAG (U140), a novel motif, exhibited the next strongest suppression of the intron-proximal 3′ss. We observed that two elements enriched in the upstream introns resembled 5′ss, such as GTAAG (U77) and GTGAG (U73). These elements also suppressed the intron-proximal 3′ss, suggesting that a splicing element resembling the 5′ss positioned close to the 3′ss may be another mechanism for alternative splicing regulation. A possible role for splice site–like sequences in exon silencing has been proposed previously [44,45]. Similarly, ISREs resembling known splicing factor-binding sites, such as TGCATG (U25), a CU-rich element (U118), a GU-rich element (U17), and TCATTTT (U149), also suppressed the intron-proximal 3′ss. Expectedly, the element resembling the branch signal, CTAAC (U22), did not alter the choice of 3′ss. In contrast, four of six controls exhibited significantly greater use of the intron-proximal 3′ss—only two significantly suppressed the intron-proximal 3′ss. If we compared the upstream ISREs with control 3 (ACACGCGGGT), the control resulting in the highest suppression of the intron-proximal 3′ss, 79% (29 of 37, including the G triplet) of the upstream ISREs significantly suppressed the intron-proximal 3′ss, relative to 0% (0 of 6) of the controls.

As the χ^2^ score distributions of the experimentally tested ISREs and the remainder of the ISREs were not significantly different by a two-sample Kolgomorov-Smirnov test (*p* > 0.05), we concluded that 84% of tested downstream ISREs and 94% of tested upstream ISREs altered splice site choice in splicing constructs in human cells. Together with published observations that ESE sequences identified by the RESCUE-ESE method [9] tended to have an opposite effect on 5′ss selection and no pronounced effect on 3′ss selection compared with ESSs [12], our data supports a rule that ISREs (like ESSs) generally inhibited intron-proximal splice sites. An important caveat in our analyses is that the ISREs, which were identified within 400 bases proximal to the exon, may not be in its natural context between competing splice sites. For example, the recent study by Ule and colleagues demonstrated that *Nova* could function as an activator or as a repressor in a context-dependent manner [40]. Although the ISREs generally suppressed the intron-proximal splice sites in this particular context, they might have a different effect in the same constructs in other cellular environments (i.e., varying concentrations of the relevant splicing factors). Alternatively, the ISREs may also have a different effect depending on the sequence context next to the ISRE. Future analysis will be required to study these contextually dependent effects on the regulation of splice site choice by ISREs.

### ISREs Exhibit Strong Positional Biases

As SREs are known to have a positional preference near splice sites [1,2,13,34], we asked if ISREs had positional biases to be conserved at particular positions in the intron (~400 bases from the exon) relative to shuffled sequences controlling for the background levels of conservation. We incorporated the following observations in designing the shuffling strategy. First, previous reports indicated that the amount of flanking intronic conservation was highest near the exon–intron boundaries and diminished further away from the exon [25–27]. Second, there are known preferences for particular nucleotides to be enriched in intronic regions close to the exons (such as G-rich tracts) [13,34]. To preserve these attributes, original and shuffled sequences were compared within relatively small windows (30 bases) as we moved downstream (upstream) of the exon to retain the overall positional biases of nucleotides. Exchanging nucleotides from 300 bases away with sequences ten bases from the splice sites would not be a sufficient nor appropriate control (nor would assuming a uniform distribution for each element). Importantly, only nucleotides from perfectly conserved regions were shuffled, retaining the positional bias for conservation (i.e., a five-base conserved tract at position +10 to +14 of the downstream intron remained preserved across mammals at the same position, but the nucleotides were exchanged); this also preserved the conservational preference for particular nucleotides.

**Figure 4:**
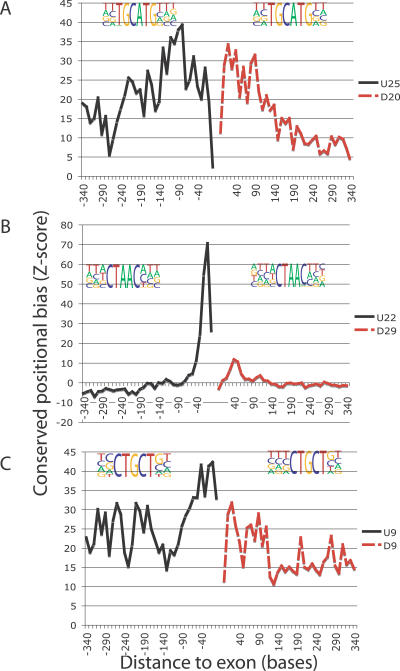
Conserved Positional Bias (Enrichment) Plots of ISREs Conserved positional bias plots of ISREs in the intronic regions upstream (black line) and downstream (red dashed line) of all exons (see Materials and Methods). The PWM representation of the ISREs is depicted above their respective plots. (A) ISRE U25 and D20 resembled the FOX1 binding site (TGCATG) and showed significant conserved enrichment throughout the 400 bases flanking exons, as well as prominent peaks in the region −130 to −40 and +15 to +120 of the intron. (B) ISRE U22 and D29 resembled the branch signal (TACTAAC). U22 showed a prominent peak at −40 to −10. D29 was significant from +15 to +80. (C) ISRE U9 and D9 resembled the “CUG” binding site of MBNL, and showed significant conserved enrichment throughout the 400 bases flanking exons. U9 peaked at −60 to 0 and D9 peaked at +0 to +120.

The positional enrichment of each ISRE compared with shuffled controls was calculated (see Materials and Methods and Protocol S1 for an example). We illustrate with three ISREs in Figure 4. The *FOX1* binding site (TGCATG, U25, and D20) was enriched across the entire 400 bases of the flanking intronic region, and peaked at 15 to 150 bases near the splice sites (Figure 4A). Unlike the *FOX1* binding site, the motif that resembled the branch signal (U22 and D29) had an asymmetric distribution around exons (Figure 4B). The motif is not enriched in most parts of the upstream intronic region, but has a sharp peak of conservation at −40 to −10 bases upstream of the 3′ss, consistent with previous studies on the location of the branch signal [13,46]. As expected, aside from the branch signal, the 3′ss and 5′ss also exhibit sharp peaks of positional enrichment (Figure S2). Noticeably, the motif also had significant biases 15–80 bases downstream of the 5′ss, consistent with results in a recent study [18]. Last, the motif resembling the *CUG-BP* binding signal (U9 and D9) was significantly enriched in the flanking intronic regions, with strong peaks at −60 to 0 upstream of the 3′ss and 0 to 120 downstream of the 5′ss (Figure 4C). In summary, we demonstrated that 84% (131 of 156) of the upstream ISREs and 76% (120 of 158) of the downstream ISREs had significantly biased positional distributions, indicating that most of the ISREs were likely to regulate splicing (Dataset S1 contains Z scores for all ISREs in the upstream and downstream intronic regions).

### ISREs Are Enriched Near Alternative Exons

Next, we asked if candidate ISREs were enriched near AS exons. Using available human transcript data, we generated a high-quality set of ~13,650 mammalian conserved exons that have evidence of exon skipping in humans. Next, we generated separate sets of upstream and downstream intronic regions from alternative exons (“alternative” intronic regions), and from constitutively spliced exons (“constitutive” intronic regions). We calculated a Z score representing the enrichment of every conserved 5- to 7-mers in the alternative intronic regions as opposed to the constitutive intronic regions, separately for upstream and downstream introns (see Materials and Methods). A high Z score indicated that the k-mer was enriched and conserved in alternative intronic regions, and each ISRE set was represented by the maximum Z score of an element in the set (Table S3). We note that as the available transcripts do not afford complete coverage across all biological conditions, the Z scores are likely underestimates, as some constitutive exons are likely to be AS in conditions with insufficient transcript evidence.

**Figure 5:**
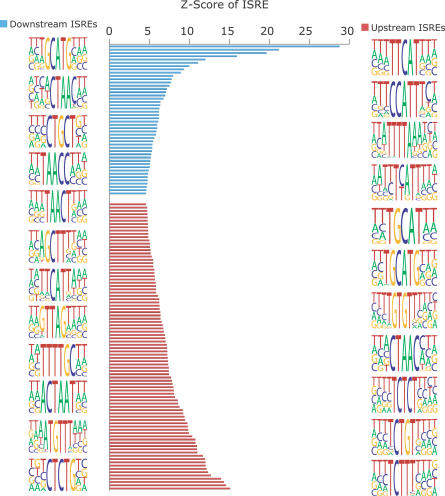
The Most Enriched ISREs Near Human AS Exons The lengths of the horizontal bars represent the maximum Z score for the ISRE (see Table S3 for all ISREs enriched near AS exons; see Protocol S1 for Z score calculation). The blue bars correspond to the downstream ISREs, represented by PWMs on the far left. PWMs were generated for each ISRE by aligning all instances of sequences that make up the ISRE in the introns proximal to exons (see Protocol S1 for details). The red bars correspond to the upstream ISREs, represented by PWMs on the far right. The PWMs are organized from most enriched (top) to least enriched (bottom) near AS exons. PWMs are used here for visualization purposes.

We identified ISREs that had high enrichment scores near alternative exons (Figure 5). We found that many of them resembled known binding sites of splicing factors involved with alternative splicing. For instance, the most enriched elements in the downstream introns were the *FOX1* binding site TGCATG (D20) and its subsequence GCATG (D50), which have been shown to be intronic splicing *cis* elements for the mammalian fibronectin and calcitonin/CGRP genes [35–37]; both mammalian *FOX1* and *FOX2* have been demonstrated to regulate TGCATG-containing exons in human neuronal cell cultures [47]. Our results indicate a potentially widespread regulation of alternative splicing by the *FOX1* family of splicing regulators: 737 exons had a conserved TGCATG in the downstream intron and 34% (248) had transcript evidence for exon skipping, compared with 8.5% for the entire set of exons (~13,650 of ~161,700 exons).

In addition, 39% (85 of 217) of mammalian exons with a conserved ISRE ACTAAC (D29) in the downstream introns had transcript evidence for exon skipping, a 5-fold enrichment relative to 8.5%. This motif has recently been shown to be enriched downstream of muscle-included alternative exons [18], and the factor that binds the motif has not been experimentally determined. ISRE TTTCAT (D150 and U149) was associated with alternative exons: 13.6% (104 of 766) of exons with conserved downstream TTTCAT and 14.7% (143 of 974) of exons with conserved upstream TTTCAT had transcript evidence for exon skipping, a 2-fold enrichment compared with 8.5%. TTTCAT resembled the *Nova* binding site. *Nova1,* a neuron-specific nuclear RNA binding protein, was first shown to recognize UCAU repeats by an immunoprecipitation and affinity elution-based RNA selection procedure [48]. Further studies using ultraviolet cross-linking and immunoprecipitation (CLIP) revealed that *Nova* proteins regulate alternative splicing of ~30 transcripts [33], but our method suggests many more potential *Nova* target exons.

We identified several motifs that contained a CTG motif enriched in introns flanking alternative exons. For instance, 14.7% (394 of 2,676) of exons containing downstream CTGCT (D9), and 14% (402 of 2,868) of exons containing upstream CTGCT (U9) had transcript evidence of exon skipping, 2-fold enrichments compared with 8.5%. We believe that these might be endogenous targets of the *MBNL* family of proteins. *MNBL* had been shown previously to bind to stretches of CUG repeats and to colocalize in vivo with CUG and CCUG repeats in myotonic dystrophy cells [42,43]. A total of 12.8% (638 of 4,978) of conserved exons proximal to TCTCT (U118) and 12% (876 of 7,368) of conserved exons proximal to TCTTT (U64) in the upstream alternative intronic regions have evidence for exon skipping, 1.5-fold over 8.5%. These resembled binding sites (UCUU and CUCUCU) for the *PTB* family of splicing regulators. *PTB* was first identified as a regulator of the *c-src* N1 exon [49,50]. Last, 18% (168 of 937) of exons proximal to TGTGTT (U17) have evidence of exon skipping, compared with 8.5%, a 2-fold enrichment of the motif. The GT-rich motif resembled the GU-rich binding site of *CELF/Bruno-like* families of RNA binding proteins [51].

In summary, 56% (88 of 156) of upstream ISREs and 30% (47 of 158) of downstream ISREs had a maximal Z score greater than 4 (a Z score of 2.5 is associated with *p* < 0.01). Of interest was our observation that downstream ISRE GTGAG (D73) was the only ISRE that appeared overrepresented in constitutive exons, due to its resemblance to the 5′ss consensus. Together, our results suggested that many ISREs resembled known SREs involved with regulation of alternative splicing. In addition, the ISRE-proximal exons represented candidate exons regulated by alternative splicing *trans*-factors.

**Figure 6:**
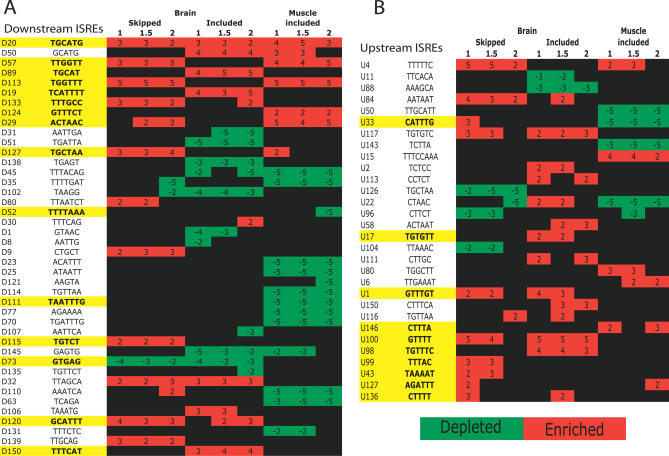
Enrichment and Depletion of ISREs Proximal to Alternative Exons Differentially Included or Skipped in Brain or Muscle from Splicing-Specific Microarray Data Intronic regions upstream and downstream of exons predicted to be included/skipped at different significance levels (see Protocol S1 for details on the dataset). The significance of enrichment (red boxes)/depletion (green boxes) are reported as log_10_ *p* values; a plus sign indicates enrichment, and a minus sign indicates depletion. For example, an ISRE represented by GCATG was enriched in intronic regions downstream of brain-included exons at *p* < 1 × 10^−4^ (reported as 4). The motifs highlighted in yellow have been experimentally verified to alter splice site choice in our reporter constructs. (A) Downstream ISREs enriched/depleted in the downstream intronic regions. (B) Upstream ISREs enriched/depleted in the upstream intronic regions.

In a recent splicing-sensitive microarray study, Sugnet et al. identified mouse alternative splicing events that were brain and muscle specific [18]. Here, we asked if ISREs were enriched in the flanking introns of the putative brain- and muscle-specific alternative events relative to a set of constitutive exons. In agreement with their findings, we observed that ISRE TGCATG was significantly enriched in the flanking introns of brain-regulated exons; TCATTTT and TTTCAT motif clusters were significantly enriched in the downstream introns of brain-included exons (Figure 6A); a TGTTTC motif cluster was significantly enriched in the upstream introns of brain-included exons (Figure 6B), similar to UG[CU]U[UG][UG][CG] identified in Sugnet et al. [18] and the ACTAAC motif was significantly enriched in the downstream introns of muscle-included exons similar to U[A/C]C[U/A]AAC identified in Sugnet et al. [18] (Figure 6A). In addition, we identified other ISREs that were significantly enriched or depleted in the flanking introns of brain and muscle alternative exons (Figure 6). Importantly, we observed that the ISREs that were enriched proximal to tissue-specific alternative exons altered splice site choice in human cells (motifs highlighted in yellow in Figure 6). This suggests that the splicing reporters in our assay were sensitive to changes by elements that were typically used in tissue-specific alternative splicing. With the availability of more splicing-specific data across tissues, cell types, and developmental stages, ISREs can be associated to various tissue-specific or developmental stage–specific regulated exons.

### Genes Containing ISRE-Proximal Exons Are Tissue Specific and Have Molecular Biases

Motivated by the findings that *Nova*-regulated alternative exons are expressed differentially in neurons and regulate synapse formation [52], we hypothesized that other genes with alternative exons containing common intronic regulatory sequences may also exhibit tissue/cell type– or developmental stage–specific expression, and share common molecular functions. As preliminary analyses indicated that genes with higher conservation in the noncoding regions were biased for neuronal expression [53] (Figure S3), we designed a sampling strategy to test for significance of differential RNA expression using a published survey of 79 human tissues and cell lines [54], as well as Gene Ontology (GO) categories, while controlling for overall intronic conservation levels (see Materials and Methods). For the purposes of our study, we considered tissues/cell lines and GO terms significantly different (enriched/depleted) using a *p*-value cutoff of 0.001.

**Figure 7:**
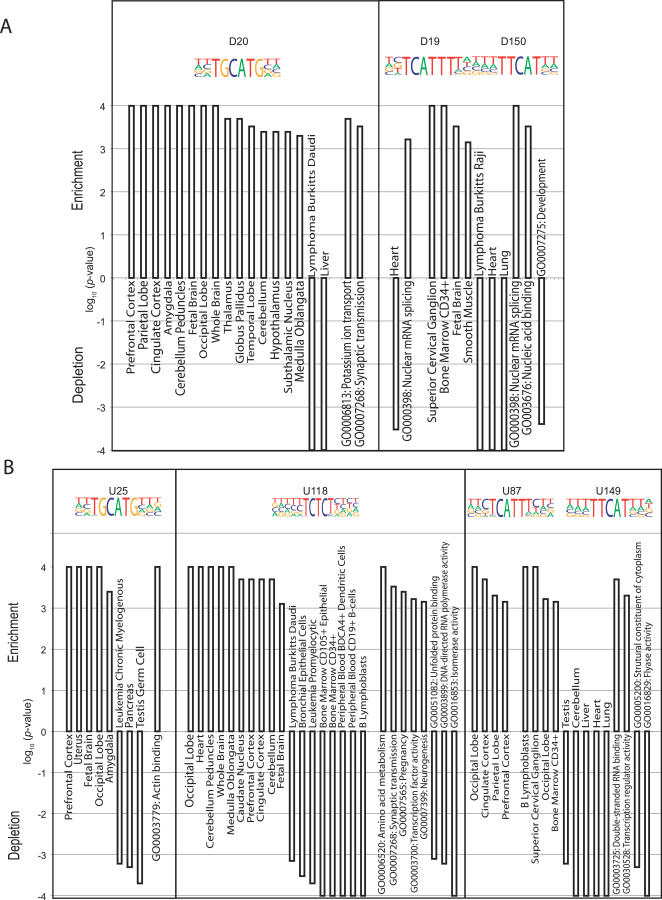
Several Examples of GO and Tissue Expression Biases for Genes Containing ISRE-Proximal Exons A gene is defined as differentially expressed in a specific tissue or cell line if its expression in the tissue or cell line is two standard deviations above the median expression across all tissues/cell lines. We denote genes that contain ISRE-proximal exons as ISRE genes. For each ISRE, the tissues or cell lines in which the ISRE genes were significantly enriched (positive value) or depleted (negative value) are illustrated, followed by the enriched or depleted GO terms. The *y*-axis is the log_10_ *p* value for significance. For notational simplicity, a minus sign indicates significantly depleted values. Please refer to Protocol S1 for details on controlling for background conservation in the intronic sequences. (A) Downstream ISREs. (B) Upstream ISREs.

Using available RNA expression data [54], we found that genes that have exons containing highly conserved TGCATG (*FOX1* binding) sites in the downstream intron were significantly biased for differential expression in many regions of the brain (*p* < 0.001). In agreement, analysis of the GO categories demonstrated significant enrichment of *FOX1* candidate genes in potassium ion transport and synaptic transmission (*p* < 0.001). Furthermore, genes with exons containing TGCATG elements in the upstream introns were also associated with high expression in the fetal brain, occipital lobe, amygdala, prefrontal cortex, and uterus (Figure 7A). This was consistent with the tissue-specific expression of *FOX1* in the brain (Figure S4).

Genes that contained conserved TTTCAT (D150, *Nova1,* and *Nova2* binding sites) in the downstream introns exhibited significant bias in the superior cervical ganglion, smooth muscle, CD34^+^ bone marrow cells, and fetal brain, and were depleted in heart, lung, and lymphoma Burkett Raji cells (Figure 7A). This was consistent with the expression patterns of both genes (i.e., *Nova1* is highly differentially expressed in all these tissues except in fetal brain, and is expressed less in heart and lung; *Nova2* is expressed highly in the superior cervical ganglion and CD34^+^ bone marrow cells, and is expressed less in smooth muscle cells and the lung; Figure S4). In addition, TTTCAT-containing introns were enriched in genes overrepresented in mRNA splicing. Surprisingly, in addition to being expressed highly in CD34^+^ bone marrow cells and B lymphoblasts, genes that contain TTTCAT sites (U87, U149) in the upstream introns of exons were significantly enriched in various subregions of the brain (occiptal and parietal lobes, cingulated cortex, superior cervical ganglion, and prefrontal cortex; Figure 7B). This may reflect differences in the regulation of alternative splicing, which depended on the binding of *Nova*1 or *Nova2* to YCAY sites in the upstream or downstream intronic regions [40]. Last, conserved CT-rich motifs (U118), resembling binding sites for *PTB* and its neuronal paralog, were enriched in the upstream introns of exons in genes that were expressed highly in various regions of the brain, and were significantly underrepresented in genes that were expressed in cells and tissues of the immune system. This is consistent with the expression of *PTB* and *nPTB: PTB* is expressed highly in the immune system and significantly depleted in the brain and subregions of the brain, and *nPTB* is enriched in both the brain and subregions of the brain (Figure S4). As *nPTB* is known to antagonize *PTB* binding [55], this suggested that *PTB* and *nPTB* might coordinately regulate target genes to achieve tissue-specific alternative splicing in a widespread manner.

ISREs in Figure 7 were not isolated examples. In total, 113 of 158 downstream ISREs (71%) and 126 of 156 upstream ISREs (81%) exhibited differential gene expression biases (Table S4 contained enriched GO terms associated with ISREs, and Table S5 contained expression biases associated with ISREs). A few studies had analyzed correlations between splicing and transcription in the global regulation of gene expression: for example, Johnson and colleagues observed that similar tissues have similar patterns of alternative splicing [5]; Pan and colleagues showed that AS profiles reflected tissue identity [22]. However, these correlations were performed on expression-level data, independent of the splicing factors or splicing *cis* elements that regulate the exons. Here, an alternative way of analyzing the correlation between transcription and alternative splicing was suggested. Our analysis revealed that exons that had conserved ISREs in common were found in tissue-specific, functionally coherent groups of genes. If we assume that the ISREs were bound by several known and as yet unidentified splicing factors, this implies that the exons with common ISREs are likely coordinately regulated. Although the associations described here between transcription and splicing are likely to be coincident (similar to *Nova* [52]) rather than a mechanistic interaction between the transcriptional machinery and splicing factors, our results provide preliminary evidence suggesting that the regulation of alternative splicing by ISREs are widespread in tissue-specific, functionally biased genes.

### Conserved ISREs and ACEs Predict Autoregulation of Splicing Factors and the Cognate RNA Binding Site

Several proteins have been reported to affect their own alternative splicing, including *PTB, Nova1, ADAR2, hnRNP A1, Srp20, SC-35, TIA1* and *TIAR,* and *FOX2* [32,56–62]. Interaction of the protein with its own pre-mRNA via its RNA binding sites typically causes alternative splicing of an exon that generates an isoform containing a premature termination codon. Autoregulation by alternative splicing might usher candidates down the nonsense-mediated decay pathway [62], potentially regulating the amount of splicing factor available in the cell. Alternatively, autoregulation may also lead to an inactive form of a protein [56].

Evolutionarily preserved AS exons have high sequence conservation in the flanking intronic sequences [25,26], which, together with additional features such as exon and intron length, splice site strength, and k-mer counts, enabled the genome-wide identification of alternative conserved exons (ACEs) in human and mouse genes with the algorithm ACEScan [27,63]. An updated genome-wide ACEScan analysis, consistent with our previous results [27], indicated that genes containing ACEs were enriched, among other categories, for RNA binding and mRNA splicing (Figure S5). This was in agreement with a study analyzing ultraconserved elements (UCEs), which identified ~200-bp long sequences that are 100% conserved in orthologous segments of human, mouse, and rat genomes [64]. The authors had discovered that the exon-overlapping UCEs were often located in genes involved in RNA binding and regulation of splicing [64]. Not surprisingly, there is substantial overlap of ACEs and UCEs (i.e., 49 of 65 UCEs that map to internal exons have positive ACEScan scores; Table S6). However, very little is known about why the flanking introns have such a high degree of sequence conservation; some speculate that the RNA secondary structure may be under selection, or that splicing regulatory motifs are being preserved [25,26].

**Figure 8:**
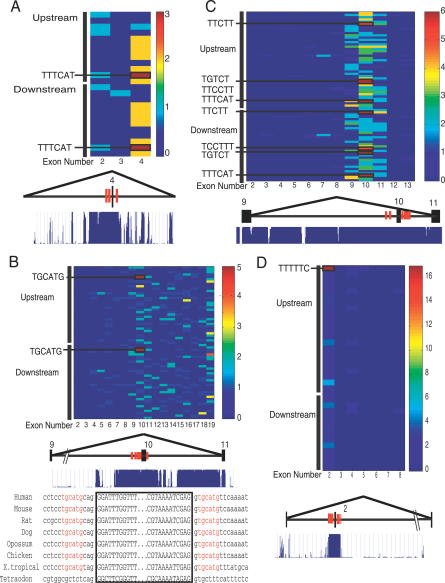
ISREs Proximal to Alternative Exons in Splicing Factors Resemble its RNA Binding Sites A total of 400 bases of intronic region flanking each internal exon were extracted for each splicing factor. Each box in the grid represents the total number of nonoverlapping occurrences of the conserved ISRE (either upstream ISRE or downstream ISRE) in the flanking intronic regions. ISREs that occur at least once in the flanks of at least one exon are retained in the grid. The exon–intron structure of selected exons are depicted below each grid. Tall vertical rectangles depict exons, and interconnecting lines represent introns; the line connecting nonadjacent exons represents alternative splicing of the middle exon. Red bars proximal to the alternative exon illustrates the ISRE. The level of conservation across multiple species (from the hg17 UCSC genome browser) is depicted below the exon. (A) *Nova1:* U149 (TTTCAT) and D150 (TTTCAT) are the most abundant ISREs in the flanking intronic region of exon 4. The flanking intronic region of exon 4 has the highest sequence conservation across mammalian genomes among all flanking intronic regions in *Nova1.* (B) *FOX1:* U25 (TGCATG) and D20 (TGCATG) are the most abundant ISREs in the flanking intronic region of exon 10 in *FOX1.* The flanking intronic region of FOX1 has the highest sequence conservation among all flanking intron regions in FOX1. The exon 10 sequence (boxed) and 15 bases of flanking intronic regions are depicted below the exon–intron structure of exons 9, 10, and 11. TGCATG sites (in red) are overlapping the 5′ss and 3′ss. (C) *nPTB:* exon 10 of *nPTB* has transcript evidence for alternative splicing, and contained highly abundant U26 (TTCTT), U121 (TGTCT), U122 (TTCCTT), U149 (TTTCAT), D21 (TTCCTT), D108 (TCCTTT), D115 (TGTCT) and D150 (TTTCAT). (D) *hnRNP C:* the flanking intronic regions of exon 2 of *hnRNP C* contains 17 copies of TTTTT (an element in U4), and is the most highly conserved among the intronic regions in *hnRNP C.*

Here, we suggest a strategy to use ISREs to discover the binding sites of splicing factors, based on this broad notion that splicing factors regulated their own alternative splicing. We illustrate with four splicing factors, *Nova1*, *nPTB*, *FOX1,* and *hnRNP C.* In published work, high-affinity *Nova*-binding sites (YCAY) were identified within exon E4 of *Nova1,* which extended into the intronic region downstream of E4. Although the intronic elements themselves were shown to be insufficient for mediating *Nova*-dependent E4 inhibition, they are synergistic to E4 in enhancing *Nova*-dependent repression [32]. When we counted the ISREs in the upstream and downstream intronic regions (400 bases each) of the internal exons in *Nova,* we found that the ISRE TTTCAT, resembling the *Nova* site, occurred with the highest frequency in E4 compared with all other ISREs (Figure 8A). Our second example is the *FOX1* gene. We discovered that exon 10 (93 bp in length) contained five conserved TGCATG sites in the 400 bases flanking the exon, the highest density of mammalian-conserved TGCATG binding sites in this region, in the genome (Figure 8B). Closer examination of the exon showed that TGCATG overlapped both the 5′ss and the 3′ss in a similar arrangement to the autoregulated exon in its paralog *FOX2* [56]. In addition, these TGCATG sites were conserved from human to Xenopus tropicalis (but not in tetraodon). Exon 10 encodes RNP1, one of the two most critical motifs of the RRM, and is skipped in skeletal muscle [65]. The third example is the *nPTB,* which, like its paralog polypyrimidine tract binding protein (*PTB*), also bound CU-rich motifs [39]. *PTB* was shown to autoregulate itself by binding to CU-rich motifs in an exon leading to a nonsense-mediated decay candidate. Here we observed that exon 10 (34 bp) of *nPTB,* which is excluded in nonneuronal tissues [66], contained high numbers of conserved TTCTT, TTCCT, and TCCTTT ISREs in the flanking introns (Figure 8C). Noticeably, ISRE TTTCAT (resembling *Nova* sites) were also present (more than five copies) in the flanking introns, indicating that in this example, we narrowed down the potential binding site to two motifs. Last, heterogeneous ribonucleoprotein C (hnRNP C) contained an AS exon 2 (evidence from Mammalian Gene Collection clones BC007052, BC008423, and BC089438) that had 17 copies of mammalian conserved TTTTT (contained in ISRE TTTTTC) in the flanking introns (Figure 8D). Exclusion or inclusion of the 26-bp long exon alters the reading frame and generates a premature termination codon. This is further supported by a study indicating that the *hnRNP C* binding site obtained by SELEX is indeed a stretch of 5 U residues [67]. Interesting, all examples shown here have high sequence conservation in the intronic regions proximal to the identified exons (Figure 8A–8D). Importantly, we found that these exons had positive ACEScan scores (*NOVA1:* 0.38; *FOX1:* 1.24; *nPTB:* 1.88; *hnRNP C:* 0.26) [27]. We suggest that we can first predict conserved alternative exons in splicing factors with ACEScan. Next, by enumerating the occurrences of ISREs proximal to the alternative exon, we can computationally predict the binding site of the splicing factor. To our knowledge, this particular computational application has not been reported for any *trans*-factor before. Whether this observation is general for all splicing factors remains to be seen, and warrants further investigation.

### Conclusion

The key findings of this work are the following. First, it reveals a set of evolutionarily conserved ISREs, and hundreds of candidate ISRE-regulated exons conserved across mammalian genomes. Second, 84% and 94% of tested ISREs were shown to suppress intron-proximal 5′ss and 3′ss in competing splice site reporter constructs in human cells, demonstrating that most ISREs can affect splicing. Combined with computational evidence that ISREs are enriched for sequences that resembled ESSs, our results suggest that a subset of ISREs might play dual roles as ESSs. Third, ~30%–50% of ISREs were enriched near alternative exons, of which several resembled most of the known binding sites of tissue-specific splicing factors. In addition, we show that ISREs were enriched near tissue-specific alternative events. Fourth, we found that for most ISREs, human genes containing ISRE-proximal exons had strong tissue expression and functional biases. Furthermore, for several ISREs that resembled known binding sites, the tissue-specific expression biases of the target genes reflected the expression of the splicing factors in a panel of human tissues. Finally, we present a novel strategy that uses ISREs and exploits the observation that many splicing factors are autoregulated to predict the binding site(s) of four splicing factors: *Nova1, nPTB, hnRNP C,* and *FOX1.* Taken together, we believe that ISREs are cornerstones in the understanding of general and tissue-specific alternative splicing. With the growing interest and importance in the detection and regulation of cell type– and stage-specific alternative splicing, ISREs will be crucial in teasing apart the combinatorial regulation of alternative events [8].

The importance of identifying SREs is underscored by the observation that as many as 50% of disease mutations in exons may affect splicing [8]. In addition to mutations that affect exonic splicing elements, we believe that, as ISREs are highly conserved across mammalian evolution, have strong positional biases, and are enriched near alternative exons, mutations in ISREs will also alter splicing. We believe knowledge of ISREs will shed light on a currently unappreciated area of human disease. Last, in the near future, we will determine the splicing *trans*-factors that bind to each ISRE, and, armed with the networks of ISRE-proximal exons, we will be able to achieve a global “RNA map” [40].

## Materials and Methods

### Databases.

*Genome sequences and alignments:* The genome sequences of human (hg17), dog (canFam1), mouse (mm5), and rat (rn3) were obtained from the University of California Santa Cruz (UCSC), as were the whole-genome multiz and pairwise alignments (http://genome.ucsc.edu). The four-way mammalian (4-mammal) whole-genome alignment (hg17, canFam1, mm5, rn3) was extracted from the 8-way vertebrate multiz alignments (hg17, panTrol1, mm5, rn3, canFam1, galGal2, fr1, danRer1).

*Exon and intronic datasets:* The lists of known human genes (obtained March 2005; knownGene.txt.gz, obtained from the UCSC ftp server [http://hgdownload.cse.ucsc.edu] containing 43,401 entries) and known isoforms (knownIsoforms.txt.gz; containing 43,286 entries in 21,397 unique isoform clusters) with annotated exon alignments to human hg17 genomic sequence were processed as follows. Conservatively, known genes that were mapped to different isoform clusters were discarded. All mRNAs aligned to hg17 that were greater than 300 bases long were clustered with the known isoforms. Genes containing less than three exons were also removed from further consideration. The 5′ and 3′ ends of introns were required to be U1- or U12-type splice sites. For each internal exon, 400 bases of flanking intronic regions were extracted from the alignments.

### Conservation scores.

The conservation score (*S*) of a sequence element of length *k* (k-mer) was represented by the nonparametric χ^2^ statistic with Yates correction, computed from the two-by-two contingency table, *T* (*T_11_:* number of occurrences of the element perfectly conserved across alignments; *T_12_:* number of occurrences of all other conserved elements of similar length; *T_21_:* number of occurrences of element in the reference genome only [human]; *T_22_:* number of occurrences of all other elements of similar length in the reference genomes). Counts in the table had to be greater than ten. To correct for multiple hypothesis testing, *p* values were multiplied by the total number of comparisons. Enriched elements with corrected *p* values less than 0.001 were considered significant.

### Experimental validation of ISREs in splicing-sensitive constructs.

We obtained previously published splicing reporters with competing 5′ss or 3′ss [12]. ISRE sequences were duplicated and inserted between two competing splice sites by restriction enzyme digestion and ligation. 293T cells were cultured with D-MEM supplemented with 10% fetal bovine serum. Transfections were carried out with Fugene (Roche, http://www.roche.com) in 24-well culture plates. A total of 0.2 μg DNA (construct) was incubated with 1 μl Fugene reagent in 20 ul serum-free media for 20–60 min at room temperature before transfection into the cells. After 48 h, cells in each well were lysed in 300 ml RNA-bee (Teltest, http://www.tel-test.com). Total RNA was isolated by chloroform extraction of the aqueous phase, followed by isopropanol precipitation as per the manufacturer's instructions. The RNA was washed in 75% ethanol and eluted in DEPC-treated water before treatment with RQ1 DNAase (Promega, http://www.promega.com). The reverse transcription (RT) reaction was carried out by using 2 μg total RNA with SuperScript III (Invitrogen, http://www.invitrogen.com). One-tenth of the product from the RT reaction was used for PCR (20–25 cycles of amplification). PCR products were run on 2% agarose gels, and the gels were scanned by a fluorescence detection imager (Fuji, http://www.fujimed.com). For each PCR product, three electronic boxes of equal size were drawn on the images: one box surrounded the larger product (intron-proximal splice site was used), one box surrounded the smaller product (intron-distal splice site was used), and one box was situated above both products to measure the background fluorescence in the gel (“background”). We calculated the amount of splicing change—the fraction of intron-distal isoform out of both isoforms—as (intron-distal/background)/(intron-distal/background + intron-proximal/background).

### Calculation of Z score for enrichment near AS exons.

Human exons with transcript evidence (human mRNAs and ESTs) for exon inclusion/exclusion were designated as skipped exons. Constitutive exons are exons with no evidence for alternative splicing (alternative 3′ss usage, 5′ss usage, intron retention, or mutually exclusive exons). We generated separate datasets for the upstream and downstream intronic regions (400 bases) flanking skipped exons. A similar dataset was generated for constitutive exons. Next, the human intronic regions were aligned to orthologous intronic regions in human, dog, rat, and mouse. The alternative splicing conservation enrichment score of a sequence element of length *k* (k-mer) was represented by the nonparametric χ^2^ statistic with Yates correction, computed from the two-by-two contingency table, *T* (*T_11_:* number of occurrences of the element perfectly conserved across alignments proximal to skipped exons; *T_12_:* number of occurrences of the element in human proximal to skipped exons; *T_21_:* number of occurrences of element perfectly conserved across alignments proximal to constitutive exons); *T_22_:* number of occurrences of the element in human proximal to constitutive exons). Counts in the table had to be greater than five. The final score for each ISRE was the maximum Z score (χ value) associated with a sequence element from the set of sequences representing the ISRE.

### Calculation of enrichment proximal to tissue-specific alternative exons.

To determine the enrichment or depletion of ISREs in mouse introns proximal to tissue-specific alternative exons, we first computed *F,* the frequency of an ISRE in a set of sequences of size *N*. For each *r* of 5,000 random selections (*R* = 5,000), *N* sequences are randomly chosen from the control set and *G_r_*, the frequency of an ISRE in the random set *R*, is computed. The *p* value for enrichment of an ISRE is computed as (1 − *E/R*), where *E* is the number of times that (*F* > *G_r_*). The *p* value for depletion is computed as (1 − *D/R*), where *D* is the number of times that (*G_r_* > *F*). Due to the small numbers of muscle-skipping events, the *p* values for depletion are not reliable, and hence the muscle-skipping events are excluded.

### GO and tissue expression analysis.

We calculated the level of sequence conservation 400 bases upstream and downstream, separately for all exons. Exons are binned into one of six upstream (or downstream) intronic conservation bins: 10%–20%, 21%–30%, 31%–40%, 41%–50%, 51%–60%, and 61%–100%. GO identifiers (IDs) for each Refseq-annotated gene were obtained from EnsMart (June 2005 release; http://www.ensembl.org). Organizational principles (molecular function, biological process) were obtained from http://www.geneontology.org. For each conserved motif *M*, we identified all exons that have the motif *M* conserved in the 400 bases upstream (or downstream) of the exon (*M* exons), and the corresponding genes that contain these exons (*M* genes). Next we calculated, for each GO term (e.g., neurogenesis, GO ID: 0007399), *F*_M,GO_, the fraction of *M* genes associated with the term. Next, *M* exons were grouped into bins by their level of upstream (or downstream) intronic conservation (as above). For 10,000 iterations, we took a similar number of randomly chosen background exons with the same upstream (or downstream) intronic conservation level from the background set (*B* exons). This controlled for the level of intronic conservation of the upstream (or downstream) introns. After retrieving the corresponding background genes (*B* genes), we determined *F*_B,GO_, the fraction of background genes that are associated with each GO term. The *p* value for the significance of enrichment (or depletion) for each GO term was computed as 1 − *N*/10,000, where *N* was the number of iterations where *F*_M,GO_ > *F*_B,GO_ (or *F*_M,GO_ < *F*_B,GO_ for depletion). Affymetrix HG-133A and GNF1B microarray gene expression from 79 human tissues and cell lines previously published by Su and colleagues [54] were obtained from the Gene Expression Atlas (http://expression.gnf.org). Mappings for Affymetrix probe identifiers were obtained from EnsMart (release 19.1). Average difference values lower than 20 were standardized to 20, as described [54]. Genes expressed in a tissue or cell line at greater than two standard deviations above the median expression across tissues or cell lines were defined as tissue-specifically expressed in that tissue or cell line. The analysis was similar to the GO analysis, except with tissue type instead of GO term. GO term and tissue/cell lines that were significantly different (*p* < 0.001) were retained.

## Supporting Information

Dataset S1 Z Scores Representing Positional Biases of ISREs in Downstream and Upstream Introns (225 KB XLS)

Figure S1 Steps for Score-Based Clustering Algorithm (1) Backwards grouping, comparing short k-mers to longer k-mers. Shorter k-mers are designed as parents of longer k-mers if the k-mer is a subsequence of, and has a higher enrichment score than the longer k-mer.
(2) Forward grouping, comparing long k-mers to shorter k-mers that are not already parents. Shorter k-mers are a child of a longer k-mer if it is a subsequence of and has a lower enrichment score than the longer k-mer. In our example, TTGGT (score of 47), TTGGTT (score of 52), and TGGTTT (score of 78) are parents, with children (above them) designated by the connected green boxes (full lines, dashed lines, and spotted lines, respectively).
(3) Families are combined if more than half of the children are shared, designating the higher-scoring parent as the new parent. In our example, TTGGT (score of 47, which is less than 52) and children are collapsed into TTGGTT (score of 52), with TTGGTT as the new parent.
(4) Families are combined if parent sequences are similar in sequence, and children of the lower-scoring parent match the sequence of the higher-scoring parent.
(5) Children that have more than one parent are uniquely associated with the higher-scoring parent.
(6) Members of each family are weighted by the number of occurrences in the genome and aligned, generating position-specific weight.
(251 KB PDF)

Figure S2 Canonical Splice Signals Exhibit Strong Positional Enrichment Positional bias (Z scores) were computed as described in Protocol S1.
(259 KB PDF)

Figure S3 Inherent Biases for Tissue Expression in Genes Containing Exons with Different Degrees of Intronic Conservation (400 Bases Upstream or Downstream of the Exon) Upstream and downstream introns are binned into six bins of conservation (10%–20%, 21%–30%, 31%–40%, 41%–50%, 51%–60%, and 61%–100%). The tissues represent the top ten tissues enriched in genes from random samplings in the respective bins relative to all the genes. Tissues with asterisks are significantly enriched at *p* < 0.05.
(183 KB PDF).

Figure S4 Gene Expression Profiles of *Nova1, Nova2, PTB1, nPTB,* and *FOX1* Across Human Tissues from Available Microarray Data Average difference values (log base 2) are plotted on the *y*-axis; tissues and cell lines are depicted on the *x*-axis.
(32KB PDF).

Figure S5 GO Categories that Are Significantly Different in Genes Containing Predicted ACEs Compared with Genes that Do Not Contain Predicted ACEs (189 KB PDF)

Table S1 Downstream ISREs and Overlap with Known SREs Parents and children comprised each ISRE identified by our method. ESSs, ESEs, ISEs, and canonical splice signals (5′ss, 3′ss, and branch signals) that overlapped ISREs are listed (see Protocol S1 for how sequence overlap is determined).
(221 KB DOC)

Table S2 Upstream ISREs and Overlap with Known SREs Parents and children comprised each ISREs identified by our method. ESSs, ESEs, ISEs, and canonical splice signals that overlap ISREs are listed (see Protocol S1 for how sequence overlap is determined).
(310 KB DOC)

Table S3 Z Scores Representing the Enrichment of ISREs Proximal to AS Exons in the Downstream Intronic Region (Downstream ISRE) or Upstream Intronic Region (Upstream ISRE) The highest Z score for the ISRE and the k-mer with the highest Z score are represented in the last two columns (see Protocol S1 for calculation of the Z scores for each ISRE).
(175 KB DOC)

Table S4 GO Terms that Are Significantly (*p* < 0.001) Enriched in Genes Containing Downstream ISRE-Proximal Exons (D1–D158) and Upstream ISRE-Proximal Exons (U1–U156) Parents are indicated here to represent the ISRE.
(488 KB DOC)

Table S5 Tissue-Expression Biases of Genes Shown are tissue-expression biases of genes containing (A) downstream ISRE-proximal exons and (B) upstream ISRE-proximal exons.
(145 KB PDF)

Table S6 UCEs that Overlap ACEScan-Positive Exons (hg17 Coordinates) (104 KB DOC)

Protocol S1 Supplementary Information for Evolutionarily Conserved Mammalian ISREs (64 KB DOC)

## Abbreviations

5′ss: 5′ splice site
3′ss: 3′ splice site
ACE: alternative conserved exon
AS: alternatively spliced
ESE: exonic splicing enhancer
ESS: exonic splicing silencer
EST: expressed sequence tag
GO: Gene Ontology
ISE: intronic splicing enhancer
SRE: splicing regulatory element
ISRE: intronic splicing regulatory element
GO: Gene Ontology
PWM: positional weight matrix
UCE: ultraconserved element
