# Correction: Discovery and Analysis of Evolutionarily Conserved Intronic Splicing Regulatory Elements

**DOI:** 10.1371/journal.pgen.0030122

**Published:** 2007-07-27

**Authors:** Gene W Yeo, Eric L. Van Nostrand, Tiffany Y Liang

In *PLoS Genetics*, volume 3, issue 5: 10.1371/journal.pgen.0030085

Mr. Eric L. Van Nostrand's name was incorrectly listed in the online citation as Nostrand ELV. The correct citation is:

Yeo GW, Van Nostrand EL, Liang TY (2007) Discovery and Analysis of Evolutionarily Conserved Intronic Splicing Regulatory Elements. PLoS Genet 3(5): e85 doi:10.1371/journal.pgen.0030085.

