# Supplementary material for: Discovery and Analysis of Evolutionarily Conserved Intronic Splicing Regulatory Elements
Source: PLoS Genet. 2007 May 25;3(5):e85. doi: 10.1371/journal.pgen.0030085 (PMC1877881; doi:10.1371/journal.pgen.0030085)

Figure S1. Score-based Clustering Strategy

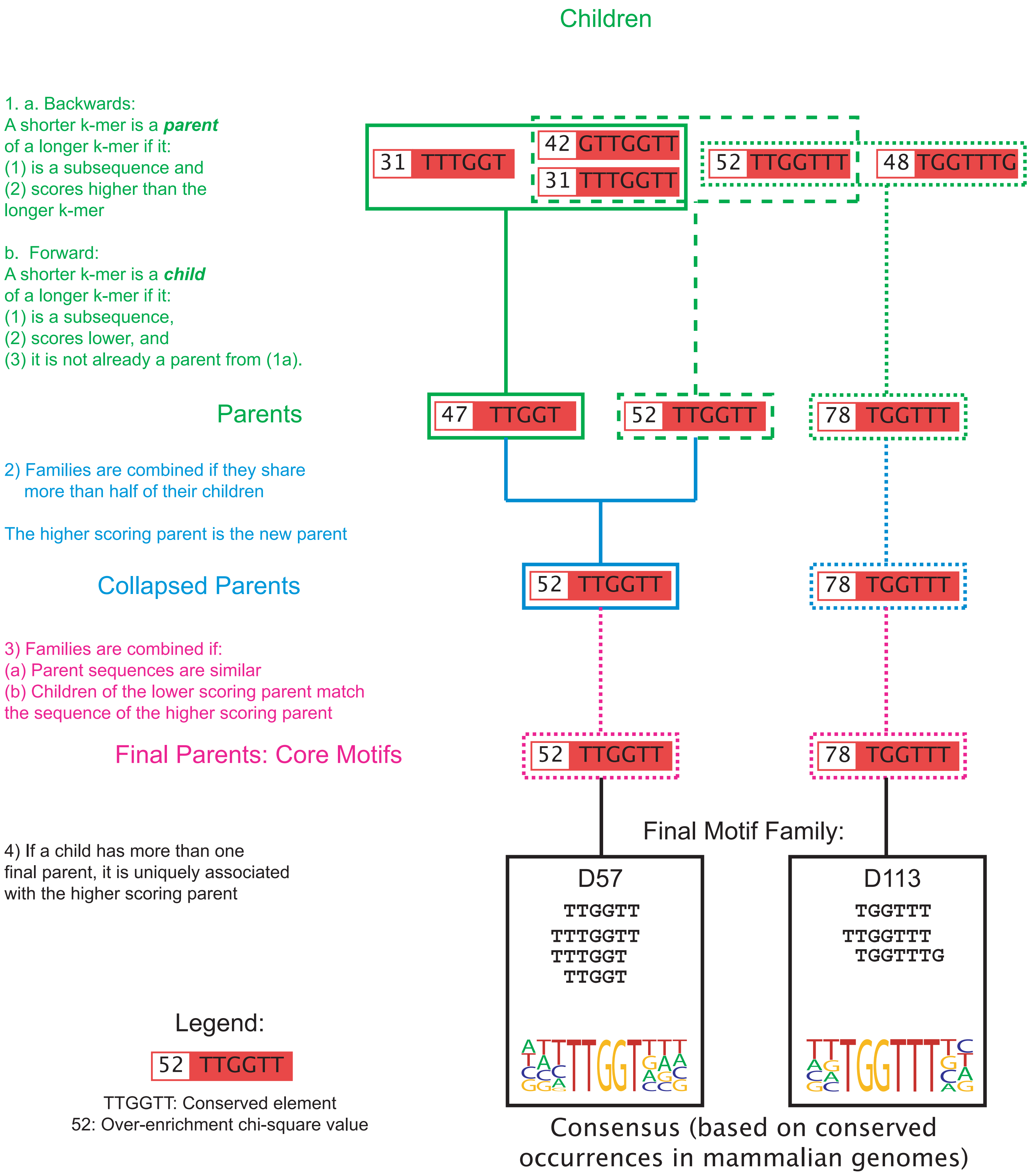

Supplement: Figure S1 — (1) Backwards grouping, comparing short k-mers to longer k-mers. Shorter k-mers are designed as parents of longer k-mers if the k-mer is a subsequence of, and has a higher enrichment score than the longer k-mer. (2) Forward grouping, comparing long k-mers to shorter k-mers that are not already parents. Shorter k-mers are a child of a longer k-mer if it is a subsequence of and has a lower enrichment score than the longer k-mer. In our example, TTGGT (score of 47), TTGGTT (score of 52), and TGGTTT (score of 78) are parents, with children (above them) designated by the connected green boxes (full lines, dashed lines, and spotted lines, respectively). (3) Families are combined if more than half of the children are shared, designating the higher-scoring parent as the new parent. In our example, TTGGT (score of 47, which is less than 52) and children are collapsed into TTGGTT (score of 52), with TTGGTT as the new parent. (4) Families are combined if parent sequences are similar in sequence, and children of the lower-scoring parent match the sequence of the higher-scoring parent. (5) Children that have more than one parent are uniquely associated with the higher-scoring parent. (6) Members of each family are weighted by the number of occurrences in the genome and aligned, generating position-specific weight. (251 KB PDF) [file pgen.0030085.sg001.pdf]
