# Supplementary figures and images for: Discovery and Analysis of Evolutionarily Conserved Intronic Splicing Regulatory Elements
Source: PLoS Genet. 2007 May 25;3(5):e85. doi: 10.1371/journal.pgen.0030085 (PMC1877881; doi:10.1371/journal.pgen.0030085)

FIGURE S2. ISREs resembling canonical splice signals exhibit positional biases

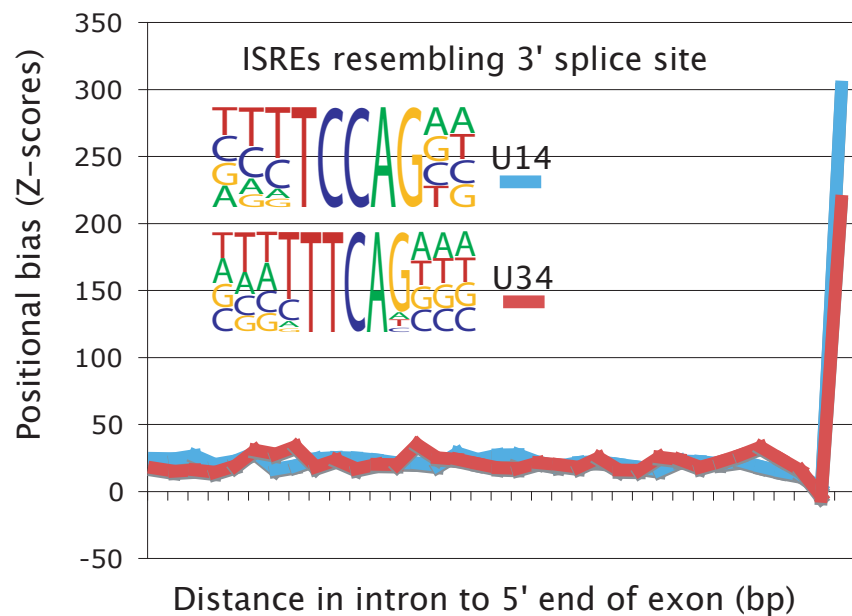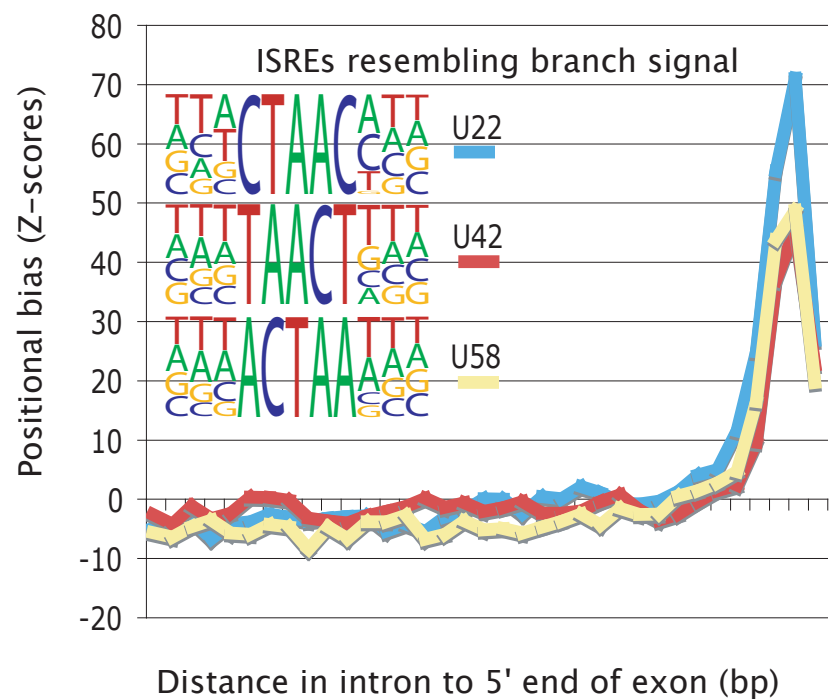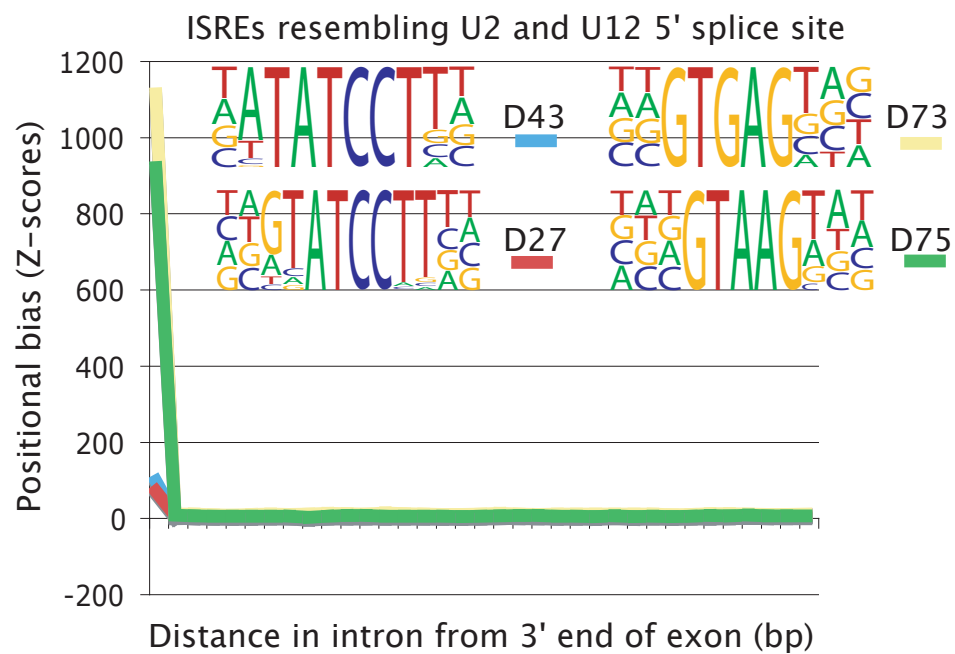

Supplement: Figure S2 — Positional bias (Z scores) were computed as described in Protocol S1. (259 KB PDF) [file pgen.0030085.sg002.pdf]

SUPPLEMENTARY FIGURE 5

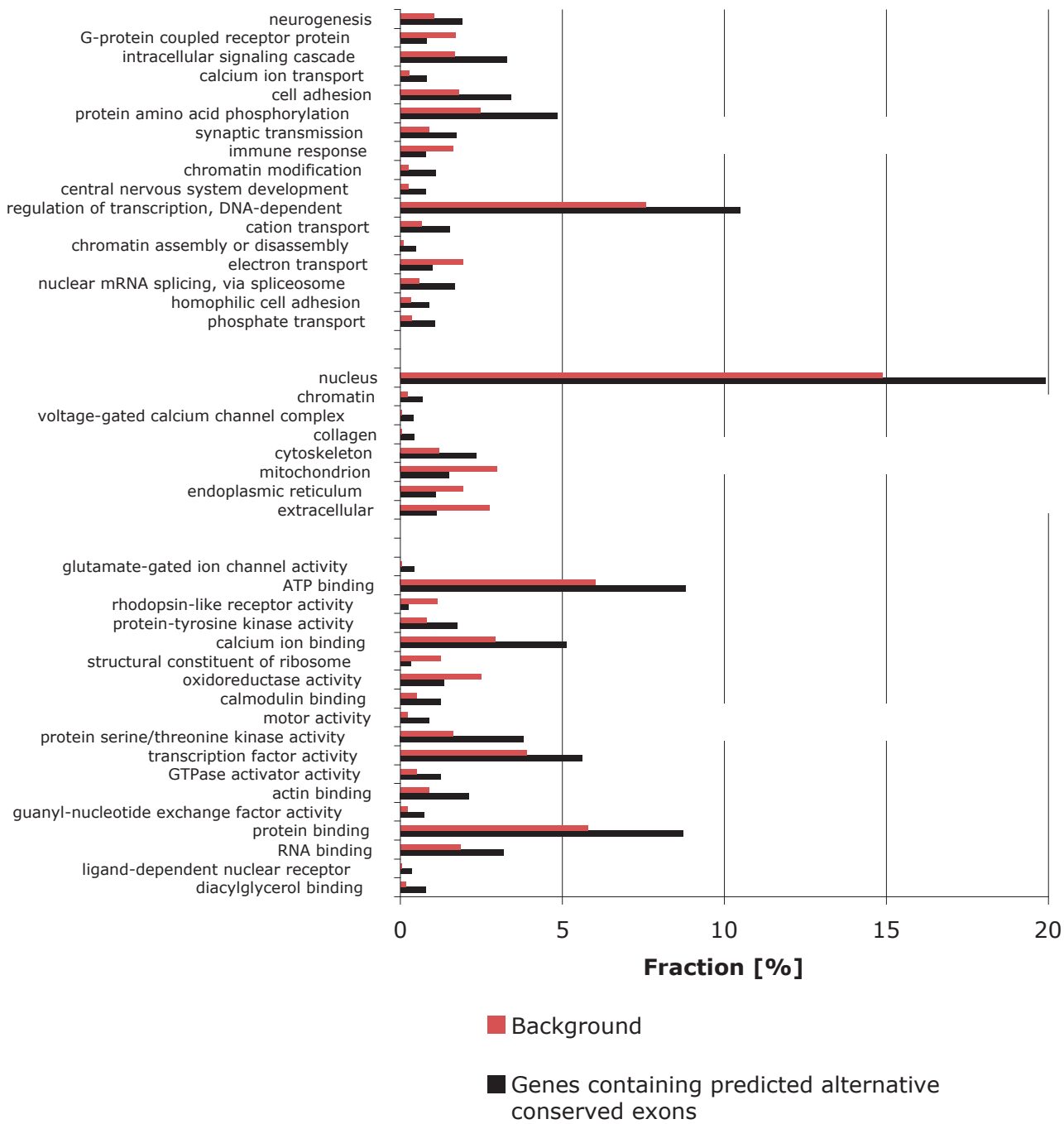

Supplement: Figure S5 — (189 KB PDF) [file pgen.0030085.sg005.pdf]
