# Supplementary material for: Discovery and Analysis of Evolutionarily Conserved Intronic Splicing Regulatory Elements
Source: PLoS Genet. 2007 May 25;3(5):e85. doi: 10.1371/journal.pgen.0030085 (PMC1877881; doi:10.1371/journal.pgen.0030085)
Supplement: Figure S3 — Upstream and downstream introns are binned into six bins of conservation (10%–20%, 21%–30%, 31%–40%, 41%–50%, 51%–60%, and 61%–100%). The tissues represent the top ten tissues enriched in genes from random samplings in the respective bins relative to all the genes. Tissues with asterisks are significantly enriched at p < 0.05. (183 KB PDF). [file pgen.0030085.sg003.pdf]

FIGURE S3. Inherent biases in expression of genes with various intronic conservation levels

Upstream intronic conservation (restricted to 400 bases upstream of exon)

0-10%	11-20%	21-30%	31-40%	41-50%	51-60%	>60%
leukemiachronicmyelogenous(k562)	PB-CD56+NKCells	Lung	*Pons	MedullaOblongata	*CerebellumPeduncles	*Amygdala
lymphomaburkittsDaudi	PB-BDCA4+Dentritic_Cells	thymus	SuperiorCervicalGanglion	PrefrontalCortex	OccipitalLobe	*fetalbrain
bonemarrow	DRG	kidney	bronchialepithelialcells	caudatenucleus	SkeletalMuscle	*PrefrontalCortex
BM-CD105+Endothelial	BM-CD33+Myeloid	Pancreas	TrigeminalGanglion	Amygdala	caudatenucleus	Thalamus
leukemiapromyelocytic(hl60)	PB-CD19+Bcells	PB-CD56+NKCells	PLACENTA	fetalbrain	fetalbrain	CingulateCortex
bronchialepithelialcells	leukemiachronicmyelogenous(k562)	PB-CD19+Bcells	OlfactoryBulb	subthalamicnucleus	TemporalLobe	OccipitalLobe
lymphnode	bronchialepithelialcells	leukemialymphoblastic(molt4)	PB-CD4+Tcells	TemporalLobe	PrefrontalCortex	TemporalLobe
721_B_lymphoblasts	721_B_lymphoblasts	WholeBrain	caudatenucleus	Tonsil	CingulateCortex	WholeBrain
Lung	BM-CD105+Endothelial	Prostate	WHOLEBLOOD	Hypothalamus	spinalcord	BM-CD71+EarlyErythroid
DRG	kidney	bonemarrow	MedullaOblongata	ParietalLobe	MedullaOblongata	PB-CD8+Tcells

Downstream intronic conservation (restricted to 400 bases downstream of exon)

0-10%	11-20%	21-30%	31-40%	41-50%	51-60%	>60%
*BM-CD34+	BM-CD105+Endothelial	WholeBrain	*OlfactoryBulb	*CerebellumPeduncles	*CingulateCortex	*CerebellumPeduncles
*721_B_lymphoblasts	leukemiachronicmyelogenous(k562)	atrioventricularnode	MedullaOblongata	PrefrontalCortex	subthalamicnucleus	*fetalbrain
*PB-CD56+NKCells	ciliaryganglion	fetalThyroid	CerebellumPeduncles	ParietalLobe	CerebellumPeduncles	PancreaticIslets
PB-BDCA4+Dentritic_Cells	PB-CD56+NKCells	lymphomaburkittsRaji	Hypothalamus	TestisLeydigCell	spinalcord	PrefrontalCortex
leukemiapromyelocytic(hl60)	leukemialymphoblastic(molt4)	Lung	SkeletalMuscle	MedullaOblongata	ParietalLobe	cerebellum
bonemarrow	PB-CD4+Tcells	testis	fetalbrain	fetalbrain	BM-CD71+EarlyErythroid	PB-CD19+Bcells
PB-CD19+Bcells	PB-CD8+Tcells	BM-CD71+EarlyErythroid	spinalcord	ColorectalAdenocarcinoma	fetalbrain	salivarygland
lymphnode	PB-BDCA4+Dentritic_Cells	CardiacMyocytes	PLACENTA	Pons	PB-CD56+NKCells	Amygdala
lymphomaburkittsDaudi	PB-CD19+Bcells	Pancreas	TemporalLobe	OccipitalLobe	Thyroid	CingulateCortex
PB-CD14+Monocytes	lymphnode	ColorectalAdenocarcinoma	bonemarrow	cerebellum	PrefrontalCortex	SmoothMuscle

* indicate significantly enriched at $P<0.05$
