# Supplementary material for: Discovery and Analysis of Evolutionarily Conserved Intronic Splicing Regulatory Elements
Source: PLoS Genet. 2007 May 25;3(5):e85. doi: 10.1371/journal.pgen.0030085 (PMC1877881; doi:10.1371/journal.pgen.0030085)

FIGURE S4. Expression of splicing factors in a panel of human tissue by available microarray data

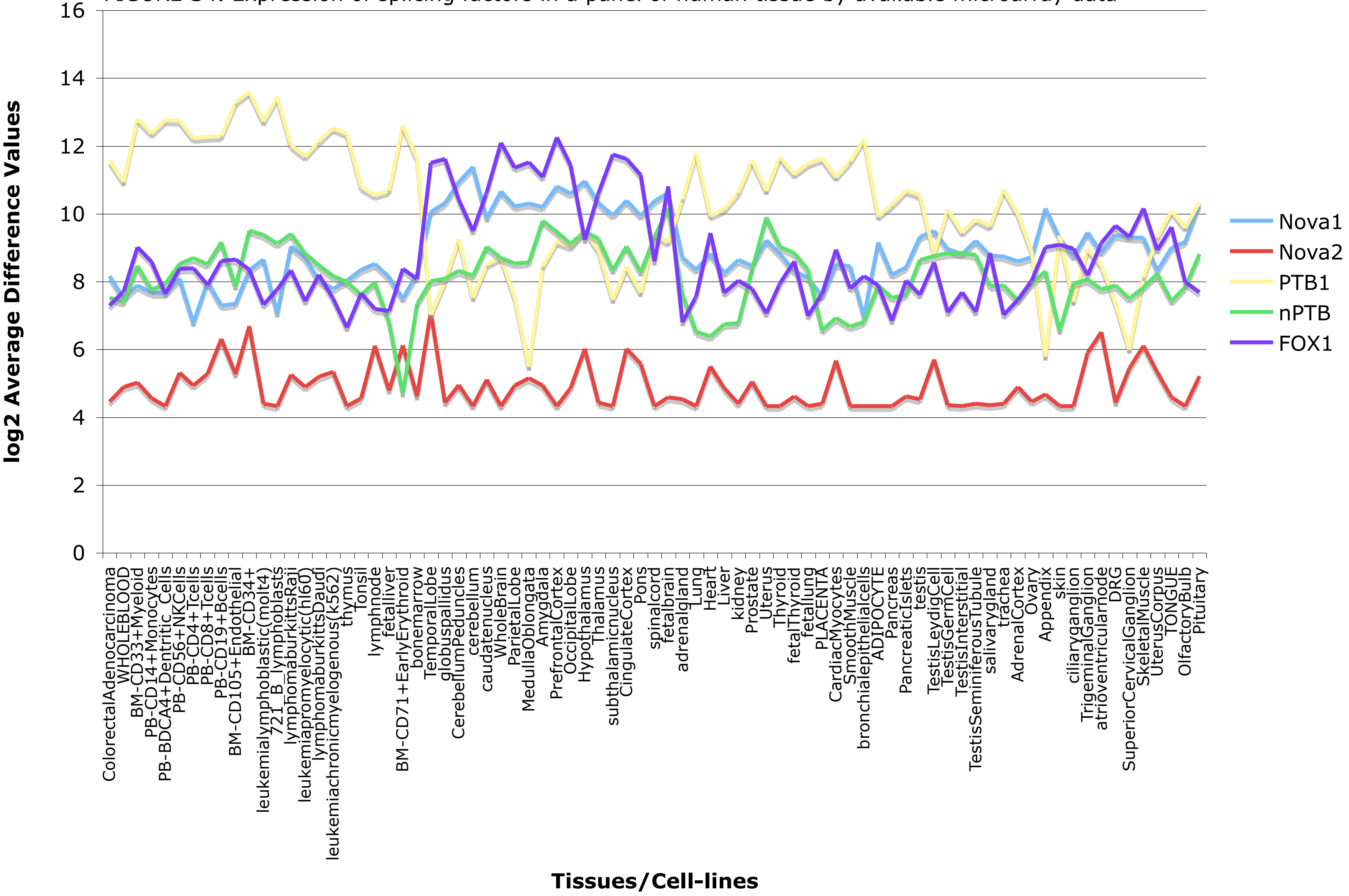

Supplement: Figure S4 — Average difference values (log base 2) are plotted on the y-axis; tissues and cell lines are depicted on the x-axis. (32KB PDF). [file pgen.0030085.sg004.pdf]
