# Supplementary material for: Discovery and Analysis of Evolutionarily Conserved Intronic Splicing Regulatory Elements
Source: PLoS Genet. 2007 May 25;3(5):e85. doi: 10.1371/journal.pgen.0030085 (PMC1877881; doi:10.1371/journal.pgen.0030085)
Supplement: Table S1 — Parents and children comprised each ISRE identified by our method. ESSs, ESEs, ISEs, and canonical splice signals (5′ss, 3′ss, and branch signals) that overlapped ISREs are listed (see Protocol S1 for how sequence overlap is determined). (221 KB DOC) [file pgen.0030085.st001.doc]

Table S1. Downstream ISREs and overlap with known splicing regulatory elements

| **Index** | **Parent** | **Children** | **Exonic Splicing Silencers** | **Exonic Splicing Enhancers** | **Intronic Splicing Enhancers** | **Canonical Splice Signals** |
| --- | --- | --- | --- | --- | --- | --- |
| 1 | GTAAC | GTAACC, GTAACA, GTAACCA, GTAACT, GTAACTA, | TAACTA |  |  | 5' splice signal |
| 2 | AAGTGT | AAGTGTC, |  |  |  | 5' splice signal |
| 3 | GTTTGT | GTTTGTT, GTTTGTC, GTTTGTA, GTTTGTG, | GTTTGT, TTTGTT, TTTGTA |  |  |  |
| 4 | ATTAACA | ATATTAA, TTAACA, CTATTAA, TATTAAC, | TATTAA, ATATTA, TTAACA |  |  |  |
| 5 | TGAAG | TTGAAGA, TTGAAG, TGAAGA, |  | TGAAGT, TGAAGA, GTGAAG, TGAAGG, ATGAAG, TGAAGC, TTGAAG, CTGAAG |  |  |
| 6 | TAACC | TAACCA, |  |  |  |  |
| 7 | TTGAAAT | TGAAATG, CTTGAAA, | GAAATG | CTTGAA |  |  |
| 8 | AATTG | AATTGGC, |  |  | AATTGT |  |
| 9 | CTGCT | CCTGCTG, CCTGCT, TGCTGCT, CTCTGCT, TTCTGCT, TCTGCT, GTCTGCT, |  | CTGCTC, CTGCTG, CCTGCT, CTCTGC, TGCTGC, TCTGCT |  |  |
| 10 | TTTATG | TTTTATG, | TTTATG, TTTTAT |  |  |  |
| 11 | TGATAAA | CTGATAA, TGATAA, |  |  |  |  |
| 12 | ATGTTT | TGTTTGA, GTTTA, GTTTAT, ATGTTTA, | TGTTTA, AGTTTA, ATGTTT, GTTTAA, GGTTTA, TGTTTG, GTTTAT, GTTTAG |  |  |  |
| 13 | TTTCCAA | TTCCAA, |  |  |  |  |
| 14 | AAGTC | AAGTCA, AAGTCT, |  |  |  | 5' splice signal |
| 15 | AAAGA | AAAGAA, TTAAAGA, TAAAGA, AAAGAAA, |  | AAGAAA, AAAGAT, AAAAGA, GAAAGA, AAAGAA, AAAGAG, AAAGAC, CAAAGA, TAAAGA |  |  |
| 16 | GTACGT | GTACGTA, GTACGTG, |  |  |  | 5' splice signal |
| 17 | GTTAAA | AAGTTAA, AGTTAAA, |  |  |  |  |
| 18 | GAGCTG | AGCTGCA, TGAGCTG, GAGCT, |  | GAGCTA, GAGCTG, GGAGCT, GAGCTT |  |  |
| 19 | TCATTTT | GTCATTT, CTCATTT, | CATTTT, TCATTT |  |  |  |
| 20 | TGCATG | TGCATGA, ATGCATG, CTGCATG, TGCATGC, TGCATGT, TGCATGG, GTGCATG, | ATGCAT |  |  |  |
| 21 | TTCTT | TTTCTTT, ATTTCTT, TTCTTTA, TTCTTA, TTTCTTA, TTTTCTT, TTCTTT, TTCTTGG, TTCTTG, TTTCTTG, | TTTTCT, TTCTTA, ATTTCT, TCTTTA, TTTCTT, TTCTTT |  |  |  |
| 22 | TTTATC | TTTATCA, |  |  |  |  |
| 23 | ACATTT | ACATTTC, |  |  |  |  |
| 24 | TGCCAGC | TTGCCAG, CTGCCAG, GCCAGCT, |  | CTGCCA, CCAGCT |  |  |
| 25 | ATAATT | TAATTTT, ATAATTG, TAATTTC, AATAAT, AATAATT, AAATAAT, | ATAATT, TAATTT, AAATAA |  |  |  |
| 26 | GTAGG | GTAGGAA, GTAGGTC, GTAGGAG, GTAGGTT, GTAGGTA, GTAGGA, GTAGGC, GTAGGT, GTAGGTG, GTAGGCA, | CGTAGG, AGTAGG, GTAGGT, GTAGGG, TGTAGG, GGTAGG, TAGGTT, TAGGTA | GTAGGA |  | 5' splice signal |
| 27 | GTATCCT | GTATCC, ATCCTTT, ATCCTT, TATCCTT, |  |  |  | U12 splice signal |
| 28 | CATTTG | CCATTTG, CATTTGA, |  |  |  |  |
| 29 | ACTAAC | ACTAACT, CACTAA, CACTAAC, CTAAC, TACTAAC, ACTAACA, CTAACC, ACTAACC, |  |  |  |  |
| 30 | TTTCAG | GTTTCAG, TTCAG, ATTTTCA, CATTTCA, CTTTCAG, TTCAGA, AATTTCA, TTTCAGA, ATTTCAG, TTCAGAA, TTTTCAG, | TTTTCA | TTCAGC, TTCAGA, ATTCAG, TCAGAA, CTTCAG |  |  |
| 31 | AATTGA | AATTGAA, AAATTGA, |  | ATTGAA |  |  |
| 32 | TTAGCA | TTAGCAA, |  |  |  |  |
| 33 | CAAAT | TCAAAT, TTCAAAT, AGCAAAT, | CAAATG | AGCAAA |  |  |
| 34 | TAATG | ATTAATG, CTAATG, TAATGG, GCTAATG, CTAATGG, TCTAATG, TAATGT, TAATGAA, | TAATGC, TAATGG, TTAATG, ATAATG, ATTAAT, TAATGA, TAATGT, GTAATG | AATGAA |  |  |
| 35 | TTTTGAT | TTGATC, TTTGATG, |  |  |  |  |
| 36 | AGAAAT | AAGAAAT, TAGAAAT, AGAAATA, GAGAAAT, AGAAATG, CAGAAAT, | GAAATG | AAGAAA, CAGAAA, GAGAAA |  |  |
| 37 | TTTCTA | TTTCTAG, ATTTCTA, TTTTCTA, TTCTAG, GTTTTCT, CATTTCT, TTTTTCT, AATTTCT, ATTTTCT, GATTTCT, | TTTTCT, ATTTCT, TTTTTC, TTTCTA, TTCTAG |  |  |  |
| 38 | TATTTC | TATTTCT, TATTTCA, ATTTCTG, ATTTCCA, TTATTTC, TATTTCC, | ATTTCT, TTATTT, TATTTC |  |  |  |
| 39 | TAACT | TAACTT, TTAACTT, TTTAACT, ATTAACT, TTTAAC, TTAACT, | TTTAAC, TTAACT, TAACTA |  |  |  |
| 40 | TGAGG | TGAGGCC, TGAGGG, TGAGGC, TGAGGA, |  | TTGAGG, TGAGGC, TGAGGA, CTGAGG, GAGGCC, GTGAGG | TGAGGG | 5' splice signal |
| 41 | TAAAAT | TAAAATT, TTTAAAA, TAAAATA, TTAAAAT, ATAAAAT, AAAATAA, TAAAATG, | TTTAAA, ATAAAA, TTAAAA, AAAATA, TAAAAT, AAATAA, AAAATG |  |  |  |
| 42 | TTTATA | TTTATAG, TTTTATA, | TTTATA, TTTTAT |  |  |  |
| 43 | TATCCT | ATATCCT, |  |  |  | U12 splice signal |
| 44 | GTTAGT | GTTAGTA, GTTAGTT, TTAGT, GTTAG, GTTAGTG, | GTTAGG, TTAGTG, TTAGTA, GTTAGT, TTTAGT, AGTTAG, GGTTAG, TTAGTT, TGTTAG, CTTAGT |  |  |  |
| 45 | TTTACAG | TTTACA, TTTTACA, | TTTACA |  |  |  |
| 46 | TATTTG | TTATTTG, TATTTGA, | TTATTT, TATTTG |  |  |  |
| 47 | GTACTGT | GTACTG, |  |  |  | 5' splice signal |
| 48 | TTAAG | CATTAAG, TTTTAAG, | TTTTAA |  |  |  |
| 49 | CATAAA | TCATAAA, | CATAAA |  |  |  |
| 50 | GCATG | AGCATG, GCATGT, GCATGAA, GCATGGC, | GGCATG |  |  |  |
| 51 | TGATTA | ATTGATT, CTGATTA, CTTGATT, |  |  |  |  |
| 52 | TTTTAAA | CATTTTA, AATTTTA, TATTTTA, TTAAATC, | TTAAAT, TTTAAA, CATTTT, TTTTAA, TATTTT, ATTTTA |  |  |  |
| 53 | CTGACT | TGACTTT, |  |  |  |  |
| 54 | ACTAAT | ACTAATG, ACTAATT, AACTAAT, CACTAAT, |  |  |  |  |
| 55 | GAGTA | GAGTATT, GAGTATC, GAGTAC, GAGTAA, GAGTAAA, GAGTACC, GAGTAT, |  | GGAGTA |  | 5' splice signal |
| 56 | TCTTAA | TCTTAAA, TCTTAAT, CTTAAT, CTTAAA, TTCTTAA, CTTAA, | TTCTTA |  |  |  |
| 57 | TTGGTT | TTTGGTT, TTTGGT, TTGGT, |  |  |  |  |
| 58 | ATATTT | AATATT, ATATTTT, | ATATTT, TATTTT |  |  |  |
| 59 | AGAGCCA | AGCCAAA, |  |  |  |  |
| 60 | TCTTT | TCTTTA, GTCTTTG, ATCTTT, TCTTTTG, TCTTTGC, TCTTTGA, TGTCTTT, TCTTTAA, | TCTTTA, TGTCTT, TTCTTT, TCTTTT |  |  |  |
| 61 | AGTTTT | AGTTTTA, | AGTTTT |  |  |  |
| 62 | GTATTT | AGTATTT, GTATT, GTATTTA, | TGTATT, GTATTT, TATTTA |  |  |  |
| 63 | TCAGA | CAGAA, CAGAAA, TCAGAG, TCAGAAA, GTCAGAG, GTCAGA, GTCAGAA, TCAGAA, |  | CCAGAA, CAGAAA, CAGAAG, TTCAGA, CAGAAT, GCAGAA, CAGAAC, ACAGAA, ATCAGA, TCAGAA |  |  |
| 64 | TAAGT | TAAGTT, TAAGTTT, TAAGTA, TAAGTTG, TAAGTCC, TAAGTGG, TAAGTAG, TAAGTGA, TAAGTAT, TAAGTC, TAAGTTC, TAAGTAA, TAAGTTA, TAAGTGT, TAAGTCT, TAAGTGC, TAAGTAC, TAAGTCA, TAAGTG, | GTAAGT, TAAGTT, AAGTAT, AAGTTT, TAAGTG | AAGTAG, AAGTGG, AAGTAC |  | 5' splice signal |
| 65 | AAGCA | AAAGCA, AAGCAT, AGCAGA, AAGCAG, AGCAG, CAAAGCA, AAGCAAA, AAGCAGA, |  | AGCAGG, AGCAAA, AAAGCA, AGCAGT, GAAGCA, AGCAGA, GAGCAG, AAGCAG, CAGCAG, AAGCAA, AGCAGC |  |  |
| 66 | TTCACAG | CACAG, TTCACA, TCACAG, |  | CACAGA |  |  |
| 67 | AGTAA | AGTAAA, AGTAAAT, |  |  |  |  |
| 68 | TCTGG | CTCTGG, TCTGGC, TTTCTGG, TCTGGG, TTCTGG, |  | ATCTGG, TCTGGA | GTCTGG, TCTGGG |  |
| 69 | AGCTTT | GCTTTGC, AAGCTTT, AGCTTTG, TAGCTTT, TTAGCTT, GCTTTT, GCTTTGA, AGCTT, |  | GAGCTT |  |  |
| 70 | TGATTTG | GATTTG, |  |  |  |  |
| 71 | TTTTGC | TTTTGCA, TTTTGCT, | TTTGCT |  |  |  |
| 72 | TAGAAA | CTAGAAA, TTAGAAA, TAGAA, TCTAGAA, AGAAAC, |  | GTAGAA, AGAAAC, TAGAAG |  |  |
| 73 | GTGAG | GTGAGCA, GTGAGAG, GTGAGGG, GTGAGCT, GTGAGC, GTGAGCC, GTGAGTG, GTGAGAA, GTGAGAC, GTGAGGA, GTGAGTA, GTGAGTT, GTGAGAT, GTGAGA, GTGAGGT, GTGAGGC, GTGAGG, |  | TGAGCA, TGAGAA, AGTGAG, TGAGGC, CGTGAG, TGAGGA, GTGAGG | TGAGGG | 5' splice signal |
| 74 | TTCTGT | TCTGT, TCTGTT, TCTGTG, ATCTGTT, TTCTGTG, TTCTGTT, | TCTGTT, TTCTGT |  |  |  |
| 75 | GTAAG | GTAAGAT, GTAAGC, GTAAGTC, GTAAGGC, GGTAAG, GTAAGAC, GTAAGTG, GTAAGTA, GTAAGAA, GTAAGT, GTAAGGG, GTAAGCA, GTAAGAG, GTAAGG, GTAAGTT, GTAAGA, GTAAGGA, GTAAGGT, GTAAGCC, GTAAGCT, | GTAAGT, TAAGTT, GTAAGG, TAAGGT, TAAGTG, GGTAAG |  |  | 5' splice signal |
| 76 | ATGAAA | ATGAAAA, ATGAA, ATGAAAT, AATGAAT, |  | ATGAAA, TATGAA, ATGAAG, AATGAA, GATGAA |  |  |
| 77 | AGAAAA | TAGAAAA, AGAAAAT, CAGAAAA, GAGAAAA, |  | CAGAAA, AGAAAA, GAAAAT, GAGAAA |  |  |
| 78 | TGAGC | TGAGCA, TGAGCT, CTGAGC, TCTGAGC, TGAGCAG, |  | TGAGCA, CTGAGC, GAGCAG |  | 5' splice signal |
| 79 | TGGCTT | TTGGCTT, TGGCTTC, GGCTT, TGGCTTT, TGGCTTG, CTGGCTT, GGCTTT, | GGCTTA, GGCTTT | CTGGCT |  |  |
| 80 | TTAATCT | TAATCT, TAATCTT, |  |  |  |  |
| 81 | AATTAT | AATTATT, ATTATTT, AATTATG, AAATTAT, ATTATT, | TTATTT, ATTATT |  |  |  |
| 82 | TGGAAAT | ATGGAAA, |  | ATGGAA, TGGAAA |  |  |
| 83 | CCACAG | CCCACAG, TCCACAG, |  |  |  |  |
| 84 | AAATGA | AATGAG, AAAATGA, AAATGAA, | AAATGA, AAAATG | AATGAA |  |  |
| 85 | GCAAGT | GCAAG, GCAAGTC, GCAAGTA, GCAAGTG, GCAAGTT, |  | GCAAGG, CAAGTA, GCAAGA, AGCAAG |  |  |
| 86 | GTAAAA | GTAAAAA, AGTAAAA, GTAAAAT, | TAAAAT, GTAAAA, TAAAAA |  |  |  |
| 87 | GTCTG | GTCTGT, |  |  | GTCTGG |  |
| 88 | AAATGT | AATGTTT, AATGTT, GAAATGT, AAATGTT, AAATGTC, | AATGTT, AAATGT, AATGTC, ATGTTT, GAAATG |  |  |  |
| 89 | TGCAT | TGCATT, TGCATTT, AATGCAT, CTGCAT, | ATGCAT, AATGCA, TGCATA |  |  |  |
| 90 | GAGAAA | AGAGAAA, GAGAA, |  | GGAGAA, TGAGAA, GAGAAG, GAGAAA, AGAGAA, GAGAAC |  |  |
| 91 | TTAGA | ATTAGA, TTAGAA, AATTAGA, ATTAGAA, | TTAGAT |  |  |  |
| 92 | TTTATAA | TTATAA, | TTTATA, TTATAA |  | TTATAA |  |
| 93 | GTTTT | TGTTTTA, GTTTTAA, GTTTTA, | AGTTTT, TGTTTT, TTTTAA, GTTTTT, GGTTTT, GTTTTG |  |  |  |
| 94 | GCTTGGC | TTGGCAA, TTGGCAT, |  |  |  |  |
| 95 | TAAGC | TAAGCT, TAAGCAT, TAAGCAA, TAAGCTT, TAAGCA, TAAGCC, TAAGCAG, TAAGCCA, AAGCCA, |  | AAGCAA, AAGCAG, AAGCCA |  | 5' splice signal |
| 96 | GTATG | GTATGTG, GTATGG, GTATGA, GTATGTA, GTATGAG, GTATGC, AGTATG, GTATGGA, AAGTATG, GTATGAA, GTATGTT, GTATGCA, GTATGT, GTATGGC, GAGTATG, GTATGTC, | TATGTC, GTATGT, TATGTT, TATGTA, TGTATG, GGTATG, AAGTAT, TATGCA | TATGAA, TATGGA, CGTATG |  | 5' splice signal |
| 97 | AAATT | TAAATT, CAAATTA, TGAAATT, GAAATTA, CAAATT, CTAAATT, TAAATTA, AAATTG, GAAATT, AAAATTC, ATAAATT, TAAATTG, GTAAATT, TAAATTT, TTAAATT, | TTAAAT, ATAAAT |  |  |  |
| 98 | GTAAT | GTAATGG, GTAATGT, AGTAATT, GTAATGC, GTAATTT, GTAATAA, GTAATGA, GTAATG, AGTAAT, GTAATTC, | TAATGC, TAATGA, TAATGG, TAATTT, GTAATG, TAATGT, TGTAAT |  |  | 5' splice signal |
| 99 | TTCTCT | TCTCTG, TTCTCTT, TCTCT, TTCTCTG, CTCTG, | TTCTCT, TCTCTA, TCTCTT | GCTCTG, ACTCTG, CCTCTG, CTCTGC |  |  |
| 100 | TGAGAA | TGAGA, TGAGAG, TGAGAAA, TGAGAAT, |  | TGAGAA, GAGAAA, ATGAGA |  | 5' splice signal |
| 101 | TTAGTT | TTAGTTT, TTTAGTT, | TTTAGT, TAGTTT, TTAGTT |  |  |  |
| 102 | TAAGG | TAAGGCT, TAAGGAG, TAAGGA, TAAGGCC, TAAGGG, TAAGGAC, TAAGGC, TAAGGAA, AAGGA, AAGGAA, AAGGAAA, | GTAAGG, TAAGGT | AAGGAC, CAAGGA, AAAGGA, AGGAAA, AAGGAT, AAGGAG, GAAGGA, AAGGAA |  | 5' splice signal |
| 103 | TGTTTAA | GTGTTT, TGTGTTT, GTTTAA, TCTGTTT, GTTTAAA, GTTTAAT, | TCTGTT, TTTAAT, TGTTTA, TTTAAA, GTGTTT, CTGTTT, GTTTAA, TGTGTT |  |  |  |
| 104 | GTCAGT | TCAGTG, GTCAGTA, GTCAGTC, TCAGTTT, GTCAGTG, TCAGTA, TCAGT, GTCAGTT, |  |  |  | 5' splice signal |
| 105 | AGAATT | TAGAATT, AAGAATT, AGAAT, AGAATTT, AAGAAT, |  | AGAATC, CAGAAT, AGAATG, AAGAAT |  |  |
| 106 | TAAATG | ATTAAAT, GTAAATG, TTAAATG, AAATGCT, TAAATGT, AAATGC, AAATGCA, TAAATGC, | TTAAAT, AAATGT, AAATGC, ATTAAA, AATGCT, TAAATG, AATGCA |  |  |  |
| 107 | AATTCA | TTAATTC, AATTC, GAATTC, TAATTCA, AATTCAA, AAATTCA, TAATTC, AATTCT, | TTAATT |  |  |  |
| 108 | TCCTTT | TTCCTT, TTTCCTT, CCTTTGC, TCCTTTG, TCCTT, | TTCCTT, TTTCCT |  |  |  |
| 109 | TAAGA | TAAGAT, TAAGAAG, TAAGAC, TAAGAAC, TTAAGA, TAAGAA, TAAGATT, TAAGAAA, TAAGAGA, TAAGAGC, TAAGATG, TAAGAAT, TAAGAG, TAAGAGG, |  | AAGAAA, AAGAGG, AAGATG, AAGAGA, AAGAAC, AAGAAG, AAGAAT |  | 5' splice signal |
| 110 | AAATCA | TAAATCA, AAATCAA, GTAAATC, |  | AATCAA |  |  |
| 111 | TAATTTG | ATTTGAT, ATTTGCT, ATTTGT, ATTTGG, | TAATTT, ATTTGT, TTTGCT |  |  |  |
| 112 | GAAATA | GAAATAA, TGAAATA, | AAATAA |  |  |  |
| 113 | TGGTTT | TTGGTTT, TGGTTTG, | GGTTTG, TGGTTT |  |  |  |
| 114 | TGTTAA | TGTTAAT, TGTTAAA, TTGTTAA, TTGTTA, | TTGTTA, TGTTAA |  |  |  |
| 115 | TGTCT | TGTCTG, TGTCTT, TTGTCTT, TGTCTGT, | TGTCTT, ATGTCT |  |  |  |
| 116 | GTTGGT | GTTGGTT, GTTGGTA, |  |  |  | 5' splice signal |
| 117 | TGAATT | TGAATTC, GAATTTG, TGAATTA, TTGAATT, ATGAATT, TGAATTT, GAATTA, GAATTAA, |  |  |  |  |
| 118 | AATTTA | AATTTAA, | ATTTAA, AATTTA |  |  |  |
| 119 | TATGT | TATGTAA, TATGTAT, TATGTA, TATGTT, | ATGTAA, TATGTC, TTATGT, GTATGT, TATGTT, TATGTA, ATGTAT, ATATGT |  |  |  |
| 120 | GCATTT | CATTTAA, CATTTAT, AGCATTT, CATTTA, | ATTTAT, ATTTAA | AGCATT |  |  |
| 121 | AAGTA | AAGTAG, AAGTATC, AAGTAC, AAGTAT, AAGTACC, AAGTAA, AAGTAAT, AAGTAAA, AAGTATT, AAGTAGA, AAGTACT, | AAGTAT | GAAGTA, AAGTAG, CAAGTA, AGTAGA, AAGTAC |  | 5' splice signal |
| 122 | GCTTCT | GCTTCTG, |  |  |  |  |
| 123 | TTCTAA | TCTAAAT, TCTAA, TCTAAT, TTTCTAA, TTCTAAT, TTCTA, TTCTAAA, TCTAAA, | TTCTAT, TTTCTA, GTTCTA, TTCTAG |  |  |  |
| 124 | GTTTCT | TTTCTGT, TGTTTC, GTTTCTT, GTTTCTG, TGTTTCT, | GTTTCT, TTTCTT, TGTTTC, TTCTGT |  |  |  |
| 125 | AGATTT | AGATTA, TAGATTT, AGATT, AAGATTT, AGATTTA, | AGATTA |  |  |  |
| 126 | GAAAAT | TGAAAAT, GAAAATG, GAAAATA, TTGAAAA, | AAAATA, AAAATG | GAAAAT |  |  |
| 127 | TGCTAA | TTTGCTA, TTGCTAA, ATGCTAA, | TTTGCT |  |  |  |
| 128 | AAGCT | AAGCTT, AAAGCT, |  | GAAGCT, AAGCTC, AAGCTA, AAGCTG, AAAGCT |  |  |
| 129 | CTTTGCT | TTGCTGA, TTGCTG, TTTGCTG, TTTGCTC, | TTTGCT | TGCTGA |  |  |
| 130 | TCTGA | TCTGACC, ATTCTGA, CTCTGA, TCTGAC, TTCTGAC, TCTGACA, TTCTGAG, TCTGAAG, CTTCTGA, TCTGAA, TCTGAAT, TTCTGAT, GCTCTGA, TCTGAG, TCTGAT, TCTGAAA, TTTCTGA, TTCTGA, TTCTGAA, |  | GCTCTG, TCTGAC, TCTGAA, CTGAAA, CTGACC, CTGAAG, ATCTGA |  |  |
| 131 | TTTCTC | ATTTCTC, TTTTCTC, TTTCTCA, TTCTCA, TTTCTCT, TTCTC, | TTTTCT, TTTCTC, TTCTCT, ATTTCT |  |  |  |
| 132 | TTTATTC | TTATTCA, | TTTATT |  |  |  |
| 133 | TTTGCC | TTTGCCA, TTTGCCT, CTTTGCC, |  |  |  |  |
| 134 | TGAAAG | TTGAAAG, GAAAG, AGAAAG, |  | GAAAGA, GGAAAG, GAAAGT, TGAAAG, GAAAGC, AGAAAG |  |  |
| 135 | TGTTCT | TGTTCTT, GTTCT, AGTTCT, | GTTCTG, GTTCTA, CGTTCT, AGTTCT |  |  |  |
| 136 | CTTTT | CTTTTA, CTTTTGA, | CTTTTT, TCTTTT |  |  |  |
| 137 | TTTTCTG | TGTTTTC, | TTTTCT, TGTTTT |  |  |  |
| 138 | TGAGT | TGAGTCC, TGAGTCT, TGAGTTT, TGAGTTG, TGAGTGC, TGAGTGG, TGAGTA, TGAGTGT, TGAGTAG, TGAGTG, TGAGTAA, TGAGTAT, TGAGTAC, TGAGTT, TGAGTGA, TGAGTTA, TGAGTTC, |  | GAGTGG |  | 5' splice signal |
| 139 | TTGCAG | CTTGCAG, TGTTGCA, CTTTGCA, |  |  |  |  |
| 140 | TAATA | TTAATAG, GTTAATA, | TAATAT, TTAATA |  |  |  |
| 141 | AGTAT | GTATC, AGTATT, AGTATC, | AGTATA, AAGTAT, TAGTAT |  |  | 5' splice signal |
| 142 | ATTCT | TAATTCT, TTATTCT, | TATTCT |  |  |  |
| 143 | TGCCTTT | TGCCTT, TTGCCT, TTGCCTT, GCCTTT, |  |  |  |  |
| 144 | ATCAAA | TCAAAA, |  | ATCAAA |  |  |
| 145 | GAGTG | GAGTGTC, GAGTGGG, GAGTGA, GAGTGTG, GAGTGT, GAGTGG, |  | GAGTGG | AGTGGG | 5' splice signal |
| 146 | TAGGT | TAGGTAT, TAGGTGG, TAGGTA, TAGGTC, TAGGTG, | GTAGGT, CTAGGT, TTAGGT, TAGGTA, AGGTAT, AGGTGG, TAGGTT |  |  | 5' splice signal |
| 147 | CTTTA | CTTTATT, | TTTATT, TCTTTA, CTTTAT |  |  |  |
| 148 | TTTAG | TTTAGAT, TTTTAGA, CTTTTAG, ATTTAG, TTTTAGC, ATTTTAG, TTTAGA, ATTTAGA, TTTAGAA, TTTAGCT, | TTTAGT, TTTTAG, ATTTTA, TTTAGG, TTAGAT, GTTTAG |  |  |  |
| 149 | TGATTTT | GATTTT, CTGATTT, |  |  |  |  |
| 150 | TTTCAT | TTCAT, ATTTCAT, TTTTCAT, | TTCATA, TTCATT, TTCATG, GTTCAT, TTTCAT, TTTTCA |  | TTTCAT |  |
| 151 | CTTTCA | TTTCAAG, CTTTC, CTTTCT, CTTTCTG, TCTTTCA, CCTTTCA, | CTTTCT |  | CCTTTC |  |
| 152 | AAGAT | AAGATT, AAGATTA, | AGATTA | GAAGAT, AAGATA, CAAGAT, AAGATG, AAAGAT, AAGATC |  |  |
| 153 | TGCTT | CTGCTTT, TGCTTC, CTGCTTC, TTGCTTG, GCTGCTT, CTGCTT, TGCTTGC, TGCTTCT, TTGCTT, TGCTTTT, TCTGCTT, TGCTTT, TGCTTG, TGCTTTG, TGTGCTT, CTGCTTG, TTGCTTC, TGCTTCA, CTTGCTT, TTGCTTT, CCTGCTT, TGCTTA, TTTGCTT, | ATGCTT, TTTGCT | TGTGCT, CCTGCT, TCTGCT |  |  |
| 154 | GTGGGT | GTGGGTA, | GTGGGT |  | GTGGGT | 5' splice signal |
| 155 | GTAAAG | GTAAAGC, GTAAAGT, TAAAGC, GTAAAGA, TAAAGT, |  | TAAAGA |  | 5' splice signal |
| 156 | CTGAA | TGCTGAA, CTGAAA, CTGAAAT, CTGAAAA, |  | TGCTGA, CTGAAC, GCTGAA, ACTGAA, CTGAAA, TCTGAA, CCTGAA, CTGAAG |  |  |
| 157 | TCTGC | TTCTGC, TCTGCA, TCTGCAG, TCTGCAT, TCTCTGC, TGTCTGC, TTTCTGC, CTCTGC, |  | TCTGCA, CTGCAG, CTCTGC, ATCTGC, TCTGCT |  |  |
| 158 | CTAAA | CTAAAT, |  |  |  |  |
