# Supplementary material for: Discovery and Analysis of Evolutionarily Conserved Intronic Splicing Regulatory Elements
Source: PLoS Genet. 2007 May 25;3(5):e85. doi: 10.1371/journal.pgen.0030085 (PMC1877881; doi:10.1371/journal.pgen.0030085)
Supplement: Table S2 — Parents and children comprised each ISREs identified by our method. ESSs, ESEs, ISEs, and canonical splice signals that overlap ISREs are listed (see Protocol S1 for how sequence overlap is determined). (310 KB DOC) [file pgen.0030085.st002.doc]

Table S2. Upstream ISREs and overlap with known splicing regulatory elements.

| **Index** | **Parent** | **Children** | **Exonic Splicing Silencers** | **Exonic Splicing Enhancers** | **Intronic Splicing Enhancers** | **Canonical Splice Signals** |
| --- | --- | --- | --- | --- | --- | --- |
| 1 | GTTTGT | GTTTGTT, GTTTGTC, GTTTGTG, | GTTTGT, TTTGTT |  |  |  |
| 2 | TCTCC | TCTCCTT, TCTCCA, |  | ATCTCC | GTCTCC, TCTCCC, CTCTCC, TCTCCT, TTCTCC, CTCCTT |  |
| 3 | GATTTT | GATTTTA, | ATTTTA |  |  |  |
| 4 | TTTTTC | CTTTTTC, ATTTTTC, GTTTTTC, TTTTT, ATTTTT, TTTTCTG, TTTTTCT, TTTTTAT, TTTTTA, TTTTTAA, | TTTTTG, TTTTTT, GTTTTT, TTTTCT, ATTTTT, TTTTTC, CTTTTT, TTTTAT, TTTTAA, TTTTTA |  |  |  |
| 5 | TAACC | TAACCA, TAACCC, TTAACCT, TAACCTC, TAACCT, |  |  |  |  |
| 6 | TTGAAAT | TGAAATG, TGAAATC, GAAATAA, TGAAATA, GAAATC, GAAATA, CTTGAAA, | AAATAA, GAAATG | CTTGAA, GAAATC |  |  |
| 7 | AAGCCA | AAAGCCA, |  | AAGCCA |  |  |
| 8 | AATTG | AATTGGC, AATTGA, |  |  |  |  |
| 9 | CTGCT | CCTGCTG, |  | CTGCTC, CTGCTG, CCTGCT, TCTGCT |  |  |
| 10 | TTTATG | TTTTATG, TTTATGC, | TTTATG, TTTTAT, TTATGC |  |  |  |
| 11 | TTCACA | TCACA, TTTCACA, |  |  |  |  |
| 12 | TGATAA | TTGATAA, TGATAAA, |  |  |  |  |
| 13 | ATGTTT | AATGTTT, ATGTTTT, TGTTTGA, | AATGTT, ATGTTT, TGTTTT, TGTTTG |  |  |  |
| 14 | TCCAG | GTTCCAG, CCTCCAG, TCTCCAG, CTTCCAG, |  | TCCAGA, GTCCAG |  | 3' splice signal |
| 15 | TTTCCAA | TCCAAA, TTCCAA, |  |  |  |  |
| 16 | TTATTTC | TATTTC, ATTTCCA, ATTATTT, TATTTCT, TATTTCA, ATTTCTG, ATTATT, TATTTCC, | ATTTCT, TTATTT, TATTTC, ATTATT |  |  |  |
| 17 | TGTGTT | GTGTTT, TGTGTTC, TTGTGTT, | GTGTTT, TTGTGT, TGTGTT |  |  |  |
| 18 | TCTTG | TCTTGT, CTTGGC, TCTTGTC, TCTTGC, TCTTGTT, TCTTGGC, TCTTGG, TCTTGCA, |  |  | CTTGGC |  |
| 19 | TTGTAA | TTGTAAT, | TGTAAT |  |  |  |
| 20 | CTTGAC | TTGACTT, TTGACAT, TTGACT, |  |  |  |  |
| 21 | TTAAAAC | TAAAAC, | TTAAAA |  |  |  |
| 22 | CTAAC | CACTAAC, AACTAAC, TCTAACA, CTAACC, GACTAAC, ACTAACC, ACTAAC, TTCTAAC, TACTAAC, CTAACA, ACTAACA, TCTAAC, CTAACCT, |  |  |  | Branch signal |
| 23 | AAAGCT | TAAAGC, TTAAAGC, TAAAGCT, |  | AAAGCT |  |  |
| 24 | TCTTC | TCTTCCT, CTCTTC, TCTTCAG, TCTTCT, TCTTCC, TCTTCA, |  | ATCTTC, CTTCAG, TCTTCA |  |  |
| 25 | TGCATG | TGCATGA, CTGCATG, TGCATGC, TGCATGT, TGCATGG, GTGCATG, GCATGTT, |  |  |  |  |
| 26 | TTCTT | TTTCTTC, CTTCTT, TTCTTGA, GTTCTT, TTTCTTA, TTCTTA, TTCTTAG, TTCTTGC, CTTTCTT, TTCTTCT, TTCTTGG, TTCTTG, TTTCTTG, TTCTTAA, ATTTCTT, TCTTCTT, TTCTTCC, TTCTTGT, TTTTCTT, TTCTTC, ATTCTT, | TTTTCT, TTCTTA, ATTTCT, TTTCTT, CTTTCT, TTCTTT |  |  |  |
| 27 | ACATTT | ACATTTT, AACATTT, | CATTTT |  |  |  |
| 28 | TTTATC | TTTTATC, TTATCAG, TTTATCA, TTATCT, TTATCTT, TTTATCT, | TTTTAT, TATCTT |  |  |  |
| 29 | ATTTTCT | TAATTTT, TATTTTC, TGATTTT, TCATTTT, | TTTTCT, TAATTT, CATTTT, TCATTT, TATTTT |  |  |  |
| 30 | GCTGACC | TGACCTG, TTGCTGA, |  | TGACCT, TGCTGA, GACCTG, CTGACC, GCTGAC |  |  |
| 31 | ATAATT | ATAAT, TAATTGT, CATAATT, TAATTTC, AATAATT, ATAATTT, GATAATT, | ATAATT, TAATTT, ATAATG, TATAAT |  |  |  |
| 32 | AACAG | TTAACAG, CCAACAG, CTAACAG, TAACAG, AAAACAG, CAACAG, TCAACAG, | TTAACA | GAACAG, AACAGA, AACAGC, AAACAG, CCAACA |  |  |
| 33 | CATTTG | CATTTGC, TGCATTT, CCATTTG, TCATTTG, | TCATTT |  |  |  |
| 34 | TTTCAG | GATTTCA, CTTCA, GTTTCA, CATTTCA, TTTTTCA, CCTTCAG, ATTTCAG, TTCAGAA, TTTTCAG, CTTCAG, GTTTCAG, TTCAG, ATTTTCA, TTCAGA, CTTTCAG, AATTTCA, TTTCAGA, | TTTTTC, TTTTCA | TTCAGC, TTCAGA, CCTTCA, ATTCAG, ACTTCA, CTTCAA, CTTCAC, TCAGAA, CTTCAG, TCTTCA |  | 3' splice signal |
| 35 | TTAGCA | TTAGCAT, |  |  |  |  |
| 36 | TTGCCT | TTGCCAG, TTTGCC, TGCCTTT, TTGCC, TGCCTT, TTGCCTT, TTTGCCA, TTTGCCT, |  |  | CTTGCC, TTGCCC |  |
| 37 | CAAAT | TCAAAT, CAAATAA, TTCAAAT, CAAATA, CAAATG, | AAATAA, CAAATG |  |  |  |
| 38 | TTTAAC | ATTTAAC, TTTAACT, TTTTAAC, TTTAACC, CTTTAAC, | TTTAAC, TTAACT, ATTTAA, TTTTAA |  |  |  |
| 39 | TAATG | CTAATG, TAATGG, GCTAATG, TAATGAT, TTAATGT, CTAATGG, TCTAATG, TAATGAC, TTAATGC, ATTAATG, CTTAATG, TAATGAA, | AATGAT, TAATGC, TAATGG, TTAATG, ATAATG, ATTAAT, TAATGA, TAATGT, GTAATG | AATGAA, AATGAC |  |  |
| 40 | AGAAAT | AAGAAAT, TAGAAAT, GAGAAA, AGAAATG, CAGAAAT, AGAAATT, | GAAATG | AAGAAA, CAGAAA, GAGAAA |  |  |
| 41 | TTTTGAT | TTGATG, TTTGATG, TTTGATA, |  |  |  |  |
| 42 | TAACT | TTAACTG, CTAACT, TTAACTC, TTAACT, TAACTG, TCTAACT, CTTAACT, TAACTT, TTAACTT, TAACTC, ATTAACT, TAACTCT, TAACTTG, CTAACTG, | TTAACT, TAACTA | AACTCT |  | Branch signal |
| 43 | TAAAAT | TAAAATT, TTTAAAA, TAAAATA, TTAAAAT, AAAATAA, TAAAATG, | TTTAAA, TTAAAA, AAAATA, TAAAAT, AAATAA, AAAATG |  |  |  |
| 44 | AATTACA | AATTAC, |  |  |  |  |
| 45 | TTCAAAA | ATTCAAA, |  |  |  |  |
| 46 | TTTATA | TTTTATA, TTATAAT, TTTATAA, TTATAA, | TTTATA, TTTTAT, TTATAA, TATAAT |  |  |  |
| 47 | CTTGTC | CTTGTCA, TTGTCAT, CTTGT, | TGTCAT, CTTGTA |  |  |  |
| 48 | TTTACAG | TTTACA, ATTTACA, TTTTACA, | TTTACA, ATTTAC |  |  |  |
| 49 | TGGATTT | TGGATT, TTTGGAT, |  | TTGGAT, TTTGGA |  |  |
| 50 | TTGCATT | GCATTTT, GCATTT, | CATTTT |  |  |  |
| 51 | TTAAG | ATTAAGC, TTTTAAG, TAAGC, TTAAGCA, TTAAGAA, ATTAAG, | TTTTAA |  |  |  |
| 52 | TTGGT | TTTTGG, TTTTGGA, | TTTTGG | TTTGGA |  |  |
| 53 | CATAAA | TCATAAA, | CATAAA |  |  |  |
| 54 | TGATTA | TTGATTA, CTGATTA, |  |  |  |  |
| 55 | GCTTTGC | AGCTTTG, AGCTTT, CTTTGCT, | TTTGCT |  |  |  |
| 56 | ATTAG | AATTAGT, CATTA, AATTAGA, TCATTAG, TTATTAG, | GATTAG, TCATTA, ATTAGG, TTATTA |  |  |  |
| 57 | TTTTAAA | CATTTTA, TATTTTA, TTAAATC, | TTAAAT, TTTAAA, CATTTT, TTTTAA, TATTTT, ATTTTA |  |  |  |
| 58 | ACTAAT | ACTAA, CACTAA, ACTAATT, ACTAATG, TACTAAT, GAACTAA, AACTAA, AAACTAA, TTACTAA, TCACTAA, | TTACTA |  |  | Branch signal |
| 59 | CTGACT | CTGACTT, TCTGACT, TGACTTT, CCCTGAC, |  | TCTGAC, CCTGAC, CCCTGA |  |  |
| 60 | ATATTT | AATATT, ATATTTT, | ATATTT, TATTTT |  |  |  |
| 61 | CATTTA | CATTTAA, TCATTTA, | ATTTAA, TCATTT |  |  |  |
| 62 | AAATCT | ATCTT, CTAATCT, TAATCT, TAATCTT, AAATCTT, AATCT, AATCTT, | TATCTT | ATCTTC |  |  |
| 63 | TTTTGGC | TTGGCAA, | TTTTGG |  |  |  |
| 64 | TCTTT | ATCTTTG, TCTTTCC, CTCTTTC, CTTCTTT, TTCTTTT, ATTCTTT, CTCTTT, TTCTTTA, TCTTTGG, GTCTT, TCTTTT, TCTTTGA, TCTTTCA, GTTCTTT, TGTCTTT, TCTCTTT, TCTTTA, TTCTTTC, TCTTTTC, TCTTTCT, GTCTTTG, TTTCTTT, TCTTTTT, ATCTTT, TCTTTTG, TCTTTGC, TCTTTC, TCTTTTA, TTCTTT, TCTTTAA, GTCTTT, | TCTTTA, CTTTCT, TCTCTT, TTTCTT, CTTTTT, TGTCTT, TCTTTT, TTCTTT |  |  |  |
| 65 | TTATTGA | TATTGAT, | TTATTG |  |  |  |
| 66 | TCAGA | CAGAA, CAGAAA, GTCAGA, TCAGAAA, TCAGAA, |  | CCAGAA, CAGAAA, CAGAAG, TTCAGA, CAGAAT, GCAGAA, CAGAAC, ACAGAA, ATCAGA, TCAGAA |  |  |
| 67 | TAAGT | TAAGTT, TAAGTTT, TAAGTAT, TAAGTA, TAAGTG, | GTAAGT, TAAGTT, AAGTAT, AAGTTT, TAAGTG |  |  |  |
| 68 | CTCTG | CTCTGG, CTCTGGG, TGCTCTG, CTCTGCA, CTCTGCT, CCTCTGC, CTCTGC, |  | GCTCTG, TCTGCA, ACTCTG, CCTCTG, TGCTCT, CTCTGC, TCTGCT | CTCTGG, CTCTGA, TCTCTG, CTCTGT, CCTCTG, TGCTCT, TCTGGG |  |
| 69 | TCTGG | TCTGGCA, TCTGGC, TCTGGA, ATCTGGC, TCTGGG, TCTGGAA, TTTCTGG, TTCTGG, |  | ATCTGG, TCTGGA, CTGGAA | CTCTGG, GTCTGG, TCTGGG |  |
| 70 | AATTC | TTAATTC, CTAATTC, AATTCAG, TAATTCA, AATTCA, TAATTC, | TTAATT | ATTCAG |  |  |
| 71 | TTTTCC | TTTCCAT, ATTTTCC, CTTTTCC, TTTTCCT, TTTCCAC, TTTCCTC, GTTTTCC, | TTTCCT | TTCCTC | TTCCTC |  |
| 72 | TTTTGC | TTTGCTA, CTTTTGC, TTTGCAC, ATTTTGC, TTTTGCT, TTTGCTC, | TTTGCT |  |  |  |
| 73 | GTGAG | GTGAGCA, GTGAGGG, GTGAGGA, GTGAGC, GTGAGTA, GTGAGTG, GTGAGTT, GTGAGA, GTGAGG, GTGAGGC, |  | TGAGCA, AGTGAG, TGAGGC, CGTGAG, TGAGGA, GTGAGG | TGAGGG, TGAGGC, GTGAGC, GGTGAG, TGAGTG, GTGAGG |  |
| 74 | TCCATTT | TTCCATT, TCCATT, CCATTTC, |  |  |  |  |
| 75 | AATTTT | AATTTTG, AATTTTC, GAATTTT, AATTTTA, | ATTTTA |  |  |  |
| 76 | CTTGATT | TTGATTC, CTTGAT, TGATTC, |  |  |  |  |
| 77 | GTAAG | GTAAGT, GTAAGGG, GTAAGC, GTAAGTC, GTAAGGC, GTAAGTG, GTAAGG, GTAAGTT, GTAAGA, GTAAGTA, GTAAGGA, GTAAGAA, GTAAGCT, | GTAAGT, TAAGTT, GTAAGG, TAAGTG, GGTAAG |  |  |  |
| 78 | ATGAAA | ATGAAAA, ATGAA, ATGAAAT, |  | ATGAAA, TATGAA, ATGAAG, AATGAA, GATGAA |  |  |
| 79 | AGAAAA | TAGAAAA, AGAAAAT, |  | AGAAAA, GAAAAT |  |  |
| 80 | TGGCTT | TTGGCTT, TGGCTTC, CTGGCT, GGCTT, TGGCTTT, TGGCTTG, CTGGCTT, GGCTTT, | GGCTTA, GGCTTT | CTGGCT | AGGCTT, GGCTTG, GGGCTT |  |
| 81 | CTCAG | TCTCAG, TCCTCAG, CCTCAG, |  | TCCTCA | CTCAGG, CCTCAG |  |
| 82 | TGGAAAT | CTGGAAA, ATGGAAA, |  | ATGGAA, TGGAAA, CTGGAA |  |  |
| 83 | AATTAT | AATTATT, AATTATG, AAATTAT, | ATTATT |  |  |  |
| 84 | AATAAT | GAATAA, GAATAAT, TGAATAA, AAATAAT, | AAATAA |  |  |  |
| 85 | TCCTAG | TTCCTAG, CTCCTAG, |  |  |  |  |
| 86 | CCACAG | CACAG, TCACAG, CCACA, TCCCACA, CCCACAG, TCCACAG, TCCACA, CTCACAG, CCCACA, TTCCACA, TTCACAG, |  | CACAGA | TCCCAC, CACAGG |  |
| 87 | TCATTTC | CTCATT, CATTTCT, GTCATTT, TCTCATT, CTCATTT, | ATTTCT, TCATTT |  |  |  |
| 88 | AAAGCA | TAAGCA, AAGCA, AAGCAAA, AAAGCAA, AAGCAG, TCAAAGC, AGCAG, |  | AGCAGG, AGCAAA, AAAGCA, AGCAGT, GAAGCA, AGCAGA, GAGCAG, AAGCAG, CAGCAG, AAGCAA, AGCAGC |  |  |
| 89 | AAATGA | AAATGAC, AAAATGA, GAAATGA, AATGAC, AAATGAA, | AAATGA, AAAATG, GAAATG | AATGAA, AATGAC |  |  |
| 90 | TTTATAG | ATTTTAT, TTATAG, | TTTATA, TTTTAT, ATTTTA |  |  |  |
| 91 | ATTAAAT | AGATTAA, ATATTAA, | TATTAA, TTAAAT, ATATTA, AGATTA, ATTAAA |  |  |  |
| 92 | CCTGCAG | CCTGCA, |  | CTGCAG, CCTGCA |  |  |
| 93 | TTACAG | CTTACAG, |  |  |  |  |
| 94 | AAATGT | AATGTT, AAAATGT, AATGTC, AAATGTT, CTAAATG, TTAAATG, TAAATGT, AATGT, AAATGTC, TAAATG, | AATGTT, TTAAAT, AATGTC, AAAATG, AAATGT, AATGTA, TAATGT, TAAATG |  |  |  |
| 95 | TGCAT | TGCATT, | ATGCAT, TGCATA |  |  |  |
| 96 | CTTCT | CTTCTG, CTTCTAA, GCTTCT, |  |  |  |  |
| 97 | TTAGAA | TAGAA, TAGAAA, TAGAATT, TTAGAAA, TTTAGAA, AGAAT, ATTAGAA, AGAATT, AGAATTT, |  | AGAATC, GTAGAA, CAGAAT, TAGAAG, AGAATG, AAGAAT |  |  |
| 98 | TGTTTC | GTGTTTC, TGTGTTT, TGTTTCA, ATGTTTC, CTGTTTC, TGTTTCT, ATTGTTT, | ATTGTT, GTGTTT, CTGTTT, GTTTCT, ATGTTT, TGTTTC, TTGTTT, TGTGTT |  |  |  |
| 99 | TTTAC | TTTTAC, TTTACT, TTTACTT, TTTTACT, | TTTACC, TTTACA, TTTACT, ATTTAC |  |  |  |
| 100 | GTTTT | CTGTTTT, TGTTTTT, GTGTTTT, TGTTTTC, GTTTTCT, GTTTTA, GTTTTGT, GTTTTCA, GTTTTGA, TGTTTTA, GTTTTAA, GTTTTAT, GTTTTG, GTTTTC, | GTTTTT, CTGTTT, TTTTCT, GTGTTT, AGTTTT, TTTTGT, TGTTTT, TTTTAT, TTTTCA, TTTTAA, GGTTTT, GTTTTG |  |  |  |
| 101 | CTTCCA | CCTTCCA, CCTTCC, TCTTCCA, |  |  | CCTTCC |  |
| 102 | TTCTAG | TTTCTA, TTTCTAG, TCTAA, ATTTCTA, TCTAAT, TTCTAAT, TTTTCTA, TTCTAGA, TTCTA, TTCTAA, CTCTAAT, TTTCTAA, CTTCTAG, TCTAATC, | TTTTCT, ATTTCT, TTCTAT, TTTCTA, GTTCTA, TTCTAG |  |  |  |
| 103 | AAATT | AAATTCT, TAAATT, CAAATTA, TGAAATT, GAAATTA, TCAAATT, CAAATT, TAAATTA, AAATTG, GAAATT, ATAAATT, TAAATTG, AAATTGA, AAATTCA, TTAAATT, | TTAAAT, ATAAAT |  |  |  |
| 104 | TTAAAC | TAAACT, TTAAACA, TTAAACT, TTTAAAC, TAAAC, TAAACTT, ATTAAAC, | TTTAAA, ATTAAA |  |  |  |
| 105 | TGAGAA | TGAGA, TGAGAAA, |  | TGAGAA, GAGAAA, ATGAGA | CTGAGA |  |
| 106 | TTTGTAG | TTGTAG, ATTTTGT, | TTTTGT, TTTGTA |  |  |  |
| 107 | GTCAGT | GTCAGTG, TTGTCAG, |  |  |  |  |
| 108 | TAAGA | TAAGAA, TAAGAAA, |  | AAGAAA |  |  |
| 109 | AAATCA | TAAATCA, AATCAAT, AAATCAA, |  | ATCAAT, AATCAA |  |  |
| 110 | TGTTGA | TGTTGAT, |  |  |  |  |
| 111 | CTTGC | CTTGCT, GCTTGCT, |  |  | CCTTGC, CTTGCC |  |
| 112 | TAATTTG | AGTAATT, ATTTGAT, GTAATTT, AATTTGA, ATTTGGC, AATTTGC, ATTTGCT, CCTAATT, AATTTGG, ATTTGG, | TAATTT, TTTGCT |  |  |  |
| 113 | CCTCT | TGCCTCT, |  | CCTCTG, ACCTCT | TGCCTC, CCCTCT, CCTCTG, CCTCTT, GCCTCT, TCCTCT, CCTCTC |  |
| 114 | TGGTTT | GTGGTTT, TCTGGTT, TTGGTTT, TGTGGTT, TGGTT, TGGTTTG, TGGTTTT, | GTGGTT, GGTTTG, ATGGTT, TGGTTT, TGGTTA, GGTTTT |  | TGGTTG |  |
| 115 | TGATTTC | GTTGATT, ATTGATT, GATTTCT, | ATTTCT |  |  |  |
| 116 | TGTTAA | TTGTTA, TTTGTTA, TGTTA, TGTTAAT, GTTAAA, TTGTTAA, TGTTAAA, TTGTTAG, GTTAAAT, | TTAAAT, TTGTTA, ATGTTA, GTGTTA, TGTTAT, TGTTAA, TGTTAG, TTTGTT |  |  |  |
| 117 | TGTGTC | TTGTGT, TGTGTCT, TTGTGTC, | TTGTGT |  |  |  |
| 118 | TCTCT | CTTCTCT, CTCTCTC, CTCTC, TCTCTTG, TTCTCTG, TCTCTT, CTCTCTG, TCTCTGC, TTCTCT, CTCTCT, TCTCTG, TCTCTC, TCTCTAG, TCTCTGG, TCTCTCC, TCTCTGT, TTCTCTT, TGTCTCT, TTCTCTC, TCTCTGA, TTTCTCT, CCTCTCT, TCTCTTC, TCTCTCT, | TTTCTC, TTCTCT, TCTCTA, TCTCTT | CTCTGC | CTCTGA, TGTCTC, CCTCTC, CTCTGG, CTCTGT, CTCTCT, TCTCTG, GTCTCT, CTCTCC |  |
| 119 | TTAACA | TAACATT, CCTTAAC, TAACA, GTTAAC, TTAACAT, TTTAACA, ATTAACA, TATTAAC, | TATTAA, TTTAAC, TTAACA |  |  |  |
| 120 | TTTGGT | TGTTTGG, TTTGGTT, TTTTGGT, TTGGTT, | GTTTGG, TTTTGG, TGTTTG |  |  |  |
| 121 | TGTCT | TGTCTC, CTGTCT, TGTCTGC, TGTCTT, TGTCTGT, TGTCTTC, GTGTCT, TGTCTTG, TTGTCTC, TGTCTAA, CTTGTCT, TTGTCTT, TGTCTCC, TCTGTCT, TGTCTG, CTGTCTG, CTGTCTT, | TGTCTT, ATGTCT |  | TGTCTG, GTCTCC, TGTCTC, TCTGTC |  |
| 122 | TTCCTT | TTCCTTA, TTTCCTT, TTCCTTG, TATCCTT, TCCTTG, TCCTTTG, TCCTTCC, TCCTTC, TCCTTAA, TTCCTTC, TCCTTAG, TCCTTGA, ATCCTTT, TCCTTT, TCCTT, CTTCCTT, TCCTTA, TGTCCTT, CTTAG, TTCCTTT, | TTCCTT, TTTCCT, CTTAGG, CTTAGT, GCTTAG | TGTCCT, CCTTGA | TGTCCT, CCTTCC, TCCTTG, CTCCTT |  |
| 123 | TGAATT | TGAATTC, CTGAATT, GAATTTG, TGAATTA, TTGAATT, TGAATTT, GAATTA, GAATTAA, |  |  |  |  |
| 124 | AATTTA | AATTTAT, AATTTAG, AATTTAA, | ATTTAT, ATTTAA, AATTTA |  |  |  |
| 125 | GTTTCT | GGTTTCT, GTTTCTT, GTTTCTG, | GTTTCT, TTTCTT |  |  |  |
| 126 | TGCTAA | TTGCTAA, CTGCTAA, ATGCTAA, TGCTAAA, |  |  |  |  |
| 127 | AGATTT | CAGATTT, AGATTTA, |  |  |  |  |
| 128 | GAAAAT | TGAAAAT, GAAAATG, TTGAAAA, | AAAATG | GAAAAT |  |  |
| 129 | GTTTAAT | TTAATCT, GTTTA, TTAATCC, TGTTTAA, GTTTAA, | TTTAAT, TGTTTA, AGTTTA, GTTTAA, GGTTTA, GTTTAT, GTTTAG |  |  |  |
| 130 | TTTGACT | CTTTGAC, GCTTTGA, |  |  |  |  |
| 131 | TCTGA | TCTGACC, ATTCTGA, CTCTGA, TCTGAC, TTCTGAC, TCTGACA, TTCTGAG, TCTGAAG, TCTGAA, TCTGAAT, TCTGAGC, TTCTGAT, GCTCTGA, CTCTGAC, TCTGAG, TCTGATG, TCTGAAA, TCTGAT, TTTCTGA, ATCTGA, TTCTGA, TTCTGAA, |  | GCTCTG, TCTGAC, CTGAGC, TCTGAA, CTGAAA, CTGACC, CTGAAG, ATCTGA | CTCTGA, TCTGAG |  |
| 132 | TCTGTT | TCTGTG, TCTGTTG, TCTGT, TCTGTTC, TTCTGTT, TTCTGTG, TCTGTGT, ATCTGTT, TTGTCC, TCTGTTT, CTGTC, TCTGTC, TGTCC, GTCTGTC, TTTCTGT, TTCTGT, TTCTGTC, TCTGTCC, | CTGTTT, TCTGTT, TTCTGT | TGTCCT, CTGTCG | TGTCCT, CTCTGT, TGTCCC, CCTGTC, TCTGTC |  |
| 133 | TTTATTC | TTATTC, TTATTCA, TTATTCT, | TTTATT, TATTCT |  |  |  |
| 134 | TGAAAG | TTGAAAG, |  | TGAAAG |  |  |
| 135 | TGTTCT | TGTTCTT, CTGTTCT, TGTTCTC, GTTCT, | GTTCTG, GTTCTA, CGTTCT, AGTTCT |  |  |  |
| 136 | CTTTT | CTTTTA, CTTTTCA, GCTTTTA, GGCTTTT, CTTTTAT, CTTTTAA, CTTTTC, TCCTTTT, CTTTTCT, CCTTTTG, GCTTTTC, GCTTTT, CTTTTGA, CCTTTT, CCTTTTA, | TTTTCT, CTTTTT, TTTTAT, TTTTAA, TCTTTT, TTTTCA, GGCTTT |  |  |  |
| 137 | ATTTGT | GATTTG, AATTTGT, ATTTGTT, TTATTTG, TGATTTG, CATTTGT, TATTTG, TTTGTGG, | TTATTT, ATTTGT, TTTGTT, TATTTG |  |  |  |
| 138 | TGAGT | TGAGTGG, TGAGTT, TGAGTA, TGAGTGA, TGAGTG, TGAGTGC, |  | GAGTGG | CTGAGT, TGAGTG, TGAGTC |  |
| 139 | CCCCAG | CCCAG, TCCCCAG, |  | ACCCAG, CCCAGG | TCCCCA, CCCAGG, TCCCAG |  |
| 140 | TTGCAG | ATTGCAG, TGTTGCA, AATTGCA, CTTGCA, CTTGCAG, TTTTGCA, CTTTGCA, GTTGCAG, |  |  |  |  |
| 141 | CTGAT | CTGATTT, TGATG, |  | CTGATC, TGATGA, ATGATG |  |  |
| 142 | TAATA | CTAATA, TTAATAG, TTAATAT, | TAATAT, TTAATA |  |  |  |
| 143 | TCTTA | CCTTAAT, TCTTAAA, CTTAAT, CCTTAA, TCTTAA, CTTAA, TCTTACA, TCTTAAT, CTTAAA, CTTAAC, TCTTAG, CTTAAAT, | TTAAAT, TTCTTA, TCTTAT |  |  |  |
| 144 | ATTCT | TAATTCT, ATTCTG, ATTCTAA, AATTCT, | TATTCT |  |  |  |
| 145 | ATCAAA | TCAAAAT, GATCAAA, |  | GATCAA, ATCAAA |  |  |
| 146 | CTTTA | CTTTATT, | TTTATT, TCTTTA, CTTTAT |  |  |  |
| 147 | TTTAG | TTTTAGA, CTTTAG, CTTTTAG, ATTTAG, TCTTTAG, ATTTTAG, ATTTAGA, TTTAGA, TGTTTAG, TTAGA, TATTTAG, GTTTTAG, | TGTTTA, TCTTTA, TTTAGG, TTAGAT, GTTTAG, TTTAGT, TTTTAG, TATTTA, ATTTTA |  |  |  |
| 148 | TTGCTG | TTTGCTG, | TTTGCT |  |  |  |
| 149 | TTTCAT | GTTCATT, TTCAT, TTCATTA, ATTTCAT, TTTTCAT, CTTCATT, | TTCATA, TTCATT, TTCATG, GTTCAT, TTTCAT, TCATTA, TTTTCA |  |  |  |
| 150 | CTTTCA | TTTCAAG, GCTTTC, TTTCAAC, CCTTTC, GCTTTCT, CTTTC, CTTTCT, CTTTCTG, CTTTCC, CCTTTCA, TTTCAAT, GCTTTCA, CTTTCCT, CTTTCCA, CTTTCAA, CCTTTCT, TCCTTTC, | TTTCCT, CTTTCT |  |  |  |
| 151 | TTCTC | TTTCTC, TCTCAT, TTTCTCA, CTTCTC, TTCTCAG, CTTCTCA, TTTTCTC, ATTTCTC, TTCTCA, CTTTCTC, TTCTCAT, TTTCTCC, TCTTCTC, TCTCA, | TTTTCT, TTTCTC, TTCTCT, ATTTCT, CTTTCT |  | TTCTCC |  |
| 152 | TGCTT | TGCTTCC, CTGCTTT, TGCTTC, CTGCTTC, GCTGCTT, CTGCTT, TGCTTGC, TGCTTCT, TTGCTT, GTTGCTT, TGCTTTT, TCTGCTT, TGCTTT, TGCTTG, AATGCT, TGCTTTG, TGTGCTT, TGCTTAA, AATGCTT, TGCTTTA, TTGCTTA, TTGCTTC, CTTGCTT, TGCTTTC, ATGCTTT, TTGCTTT, TGCTTA, TTTGCTT, | AATGCT, ATGCTT, TTTGCT | TGTGCT, TCTGCT |  |  |
| 153 | TCTGC | TTCTGC, TCTGCA, TTCTGCT, TTCTGCA, TTTCTGC, TCTGCT, TCTGCC, TCTGCCA, TCTGCTG, |  | TCTGCA, CTGCCA, CTGCTG, CTCTGC, ATCTGC, TCTGCT | TCTGCC |  |
| 154 | CTGAA | TGAAG, CTGAAA, CTGAAAT, |  | TGAAGT, TGAAGA, GTGAAG, CTGAAC, GCTGAA, TGAAGG, ATGAAG, TGAAGC, ACTGAA, TCTGAA, CTGAAA, TTGAAG, CCTGAA, CTGAAG |  |  |
| 155 | GTAGGT | GTAGGTG, | GTAGGT |  |  |  |
| 156 | CTAAA | GCTAA, TCTAAAT, TTCTAAA, GCTAAAT, CTAAAT, TCTAAAA, GCTAAA, CTAAAA, AAGCTAA, TCTAAA, |  | AAGCTA |  |  |
