# Supplementary material for: Discovery and Analysis of Evolutionarily Conserved Intronic Splicing Regulatory Elements
Source: PLoS Genet. 2007 May 25;3(5):e85. doi: 10.1371/journal.pgen.0030085 (PMC1877881; doi:10.1371/journal.pgen.0030085)
Supplement: Table S3 — The highest Z score for the ISRE and the k-mer with the highest Z score are represented in the last two columns (see Protocol S1 for calculation of the Z scores for each ISRE). (175 KB DOC) [file pgen.0030085.st003.doc]

Table S3. ISREs enriched proximal to alternatively spliced exons.

|  | **Index** | **Parent** | **Highest Z-score** | **Child with highest Z-score** |
| --- | --- | --- | --- | --- |
| Downstream ISRE | 50 | GCATG | 28.79 | GCATG |
| Downstream ISRE | 20 | TGCATG | 21.20 | TGCATG |
| Downstream ISRE | 89 | TGCAT | 19.67 | TGCAT |
| Downstream ISRE | 29 | ACTAAC | 15.96 | CTAAC |
| Downstream ISRE | 153 | TGCTT | 12.02 | TGCTT |
| Downstream ISRE | 6 | TAACC | 11.09 | TAACC |
| Downstream ISRE | 9 | CTGCT | 10.00 | CTGCT |
| Downstream ISRE | 157 | TCTGC | 9.30 | TCTGC |
| Downstream ISRE | 39 | TAACT | 8.93 | TAACT |
| Downstream ISRE | 122 | GCTTCT | 7.99 | GCTTCT |
| Downstream ISRE | 136 | CTTTT | 7.78 | CTTTT |
| Downstream ISRE | 69 | AGCTTT | 7.70 | AGCTT |
| Downstream ISRE | 79 | TGGCTT | 7.52 | GGCTT |
| Downstream ISRE | 150 | TTTCAT | 7.18 | TTCAT |
| Downstream ISRE | 99 | TTCTCT | 7.07 | TCTCT |
| Downstream ISRE | 115 | TGTCT | 7.00 | TGTCT |
| Downstream ISRE | 44 | GTTAGT | 6.77 | GTTAG |
| Downstream ISRE | 98 | GTAAT | 6.40 | GTAATG |
| Downstream ISRE | 34 | TAATG | 6.36 | TAATG |
| Downstream ISRE | 56 | TCTTAA | 6.26 | CTTAA |
| Downstream ISRE | 68 | TCTGG | 6.21 | TCTGG |
| Downstream ISRE | 154 | GTGGGT | 6.21 | GTGGGTA |
| Downstream ISRE | 71 | TTTTGC | 6.20 | TTTTGC |
| Downstream ISRE | 93 | GTTTT | 6.10 | GTTTT |
| Downstream ISRE | 54 | ACTAAT | 5.98 | ACTAAT |
| Downstream ISRE | 74 | TTCTGT | 5.97 | TCTGT |
| Downstream ISRE | 87 | GTCTG | 5.89 | GTCTG |
| Downstream ISRE | 33 | CAAAT | 5.81 | CAAAT |
| Downstream ISRE | 57 | TTGGTT | 5.64 | TTGGT |
| Downstream ISRE | 97 | AAATT | 5.46 | AAATT |
| Downstream ISRE | 151 | CTTTCA | 5.37 | CTTTC |
| Downstream ISRE | 63 | TCAGA | 5.35 | TCAGA |
| Downstream ISRE | 102 | TAAGG | 5.33 | TAAGGG |
| Downstream ISRE | 60 | TCTTT | 5.22 | TCTTT |
| Downstream ISRE | 131 | TTTCTC | 5.16 | TTCTC |
| Downstream ISRE | 140 | TAATA | 5.10 | TAATA |
| Downstream ISRE | 21 | TTCTT | 5.06 | TTCTTG |
| Downstream ISRE | 88 | AAATGT | 5.04 | AATGTT |
| Downstream ISRE | 67 | AGTAA | 4.92 | AGTAA |
| Downstream ISRE | 113 | TGGTTT | 4.84 | TGGTTT |
| Downstream ISRE | 1 | GTAAC | 4.79 | GTAAC |
| Downstream ISRE | 129 | CTTTGCT | 4.77 | TTGCTG |
| Downstream ISRE | 12 | ATGTTT | 4.76 | GTTTA |
| Downstream ISRE | 65 | AAGCA | 4.68 | AGCAG |
| Downstream ISRE | 3 | GTTTGT | 4.62 | GTTTGT |
| Downstream ISRE | 142 | ATTCT | 4.57 | ATTCT |
| Upstream ISRE | 95 | TGCAT | 15.10 | TGCAT |
| Upstream ISRE | 4 | TTTTTC | 14.60 | TTTTT |
| Upstream ISRE | 121 | TGTCT | 14.32 | TGTCT |
| Upstream ISRE | 118 | TCTCT | 13.97 | TCTCT |
| Upstream ISRE | 25 | TGCATG | 12.72 | TGCATG |
| Upstream ISRE | 136 | CTTTT | 12.32 | CTTTT |
| Upstream ISRE | 132 | TCTGTT | 12.16 | TCTGT |
| Upstream ISRE | 26 | TTCTT | 12.05 | TTCTT |
| Upstream ISRE | 150 | CTTTCA | 12.03 | CTTTC |
| Upstream ISRE | 96 | CTTCT | 11.97 | CTTCT |
| Upstream ISRE | 152 | TGCTT | 11.68 | TGCTT |
| Upstream ISRE | 64 | TCTTT | 11.01 | TCTTT |
| Upstream ISRE | 151 | TTCTC | 10.96 | TTCTC |
| Upstream ISRE | 9 | CTGCT | 10.95 | CTGCT |
| Upstream ISRE | 58 | ACTAAT | 10.76 | ACTAA |
| Upstream ISRE | 100 | GTTTT | 10.75 | GTTTT |
| Upstream ISRE | 22 | CTAAC | 10.35 | CTAAC |
| Upstream ISRE | 2 | TCTCC | 9.94 | TCTCC |
| Upstream ISRE | 153 | TCTGC | 9.87 | TCTGC |
| Upstream ISRE | 80 | TGGCTT | 9.79 | GGCTT |
| Upstream ISRE | 24 | TCTTC | 9.77 | TCTTCT |
| Upstream ISRE | 113 | CCTCT | 9.44 | CCTCT |
| Upstream ISRE | 47 | CTTGTC | 9.40 | CTTGT |
| Upstream ISRE | 5 | TAACC | 9.24 | TAACC |
| Upstream ISRE | 18 | TCTTG | 9.19 | TCTTGT |
| Upstream ISRE | 17 | TGTGTT | 8.77 | TGTGTT |
| Upstream ISRE | 149 | TTTCAT | 8.60 | TTCAT |
| Upstream ISRE | 68 | CTCTG | 8.56 | CTCTG |
| Upstream ISRE | 122 | TTCCTT | 8.19 | TCCTTT |
| Upstream ISRE | 42 | TAACT | 8.10 | TAACT |
| Upstream ISRE | 72 | TTTTGC | 8.07 | TTTTGC |
| Upstream ISRE | 52 | TTGGT | 8.01 | TTGGT |
| Upstream ISRE | 71 | TTTTCC | 7.89 | TTTTCC |
| Upstream ISRE | 29 | ATTTTCT | 7.81 | TCATTTT |
| Upstream ISRE | 94 | AAATGT | 7.71 | AATGT |
| Upstream ISRE | 119 | TTAACA | 7.47 | TAACA |
| Upstream ISRE | 13 | ATGTTT | 7.43 | ATGTTT |
| Upstream ISRE | 74 | TCCATTT | 7.42 | TCCATT |
| Upstream ISRE | 99 | TTTAC | 7.34 | TTTAC |
| Upstream ISRE | 114 | TGGTTT | 7.31 | TGGTT |
| Upstream ISRE | 102 | TTCTAG | 7.28 | TCTAA |
| Upstream ISRE | 98 | TGTTTC | 7.25 | TGTTTC |
| Upstream ISRE | 1 | GTTTGT | 7.24 | GTTTGT |
| Upstream ISRE | 144 | ATTCT | 7.20 | ATTCT |
| Upstream ISRE | 51 | TTAAG | 7.17 | TTAAG |
| Upstream ISRE | 135 | TGTTCT | 7.06 | GTTCT |
| Upstream ISRE | 57 | TTTTAAA | 7.00 | CATTTTA |
| Upstream ISRE | 116 | TGTTAA | 6.95 | TGTTA |
| Upstream ISRE | 154 | CTGAA | 6.79 | CTGAA |
| Upstream ISRE | 146 | CTTTA | 6.74 | CTTTA |
| Upstream ISRE | 38 | TTTAAC | 6.67 | TTTAAC |
| Upstream ISRE | 141 | CTGAT | 6.54 | CTGAT |
| Upstream ISRE | 131 | TCTGA | 6.41 | TCTGA |
| Upstream ISRE | 3 | GATTTT | 6.38 | GATTTT |
| Upstream ISRE | 56 | ATTAG | 6.36 | CATTA |
| Upstream ISRE | 111 | CTTGC | 6.30 | CTTGC |
| Upstream ISRE | 36 | TTGCCT | 6.29 | TTGCC |
| Upstream ISRE | 125 | GTTTCT | 6.23 | GTTTCT |
| Upstream ISRE | 70 | AATTC | 6.23 | AATTC |
| Upstream ISRE | 148 | TTGCTG | 6.08 | TTGCTG |
| Upstream ISRE | 126 | TGCTAA | 5.83 | TGCTAA |
| Upstream ISRE | 140 | TTGCAG | 5.82 | TTTTGCA |
| Upstream ISRE | 39 | TAATG | 5.81 | TAATG |
| Upstream ISRE | 137 | ATTTGT | 5.78 | TATTTG |
| Upstream ISRE | 16 | TTATTTC | 5.68 | ATTATT |
| Upstream ISRE | 55 | GCTTTGC | 5.68 | GCTTTGC |
| Upstream ISRE | 78 | ATGAAA | 5.64 | ATGAA |
| Upstream ISRE | 27 | ACATTT | 5.62 | ACATTT |
| Upstream ISRE | 28 | TTTATC | 5.49 | TTATCT |
| Upstream ISRE | 34 | TTTCAG | 5.49 | CTTCA |
| Upstream ISRE | 156 | CTAAA | 5.46 | CTAAA |
| Upstream ISRE | 87 | TCATTTC | 5.29 | CATTTCT |
| Upstream ISRE | 120 | TTTGGT | 5.17 | TTTGGT |
| Upstream ISRE | 60 | ATATTT | 5.13 | ATATTTT |
| Upstream ISRE | 62 | AAATCT | 5.09 | ATCTT |
| Upstream ISRE | 20 | CTTGAC | 5.08 | TTGACT |
| Upstream ISRE | 110 | TGTTGA | 5.01 | TGTTGA |
| Upstream ISRE | 76 | CTTGATT | 4.87 | TGATTC |
| Upstream ISRE | 69 | TCTGG | 4.87 | TCTGG |
| Upstream ISRE | 48 | TTTACAG | 4.84 | TTTACA |
| Upstream ISRE | 59 | CTGACT | 4.81 | CTGACT |
| Upstream ISRE | 104 | TTAAAC | 4.80 | TTTAAAC |
| Upstream ISRE | 117 | TGTGTC | 4.79 | TTGTGT |
| Upstream ISRE | 101 | CTTCCA | 4.78 | CCTTCC |
| Upstream ISRE | 133 | TTTATTC | 4.75 | TTATTC |
| Upstream ISRE | 143 | TCTTA | 4.72 | CTTAAA |
| Upstream ISRE | 11 | TTCACA | 4.71 | TCACA |
| Upstream ISRE | 8 | AATTG | 4.60 | AATTG |
