# Supplementary material for: Discovery and Analysis of Evolutionarily Conserved Intronic Splicing Regulatory Elements
Source: PLoS Genet. 2007 May 25;3(5):e85. doi: 10.1371/journal.pgen.0030085 (PMC1877881; doi:10.1371/journal.pgen.0030085)
Supplement: Table S4 — Parents are indicated here to represent the ISRE. (488 KB DOC) [file pgen.0030085.st004.doc]

Table S4. Gene Ontology terms enriched in genes containing downstream ISRE-proximal exons, and upstream ISRE-proximal exons.

| **Index** | **Parent** | **Molecular Function** | **Biological Process** | **Cellular component** |
| --- | --- | --- | --- | --- |
| D1 | ***GTAAC*** | - | GO0008380|P|RNA splicing | - |
| D2 | ***AAGTGT*** | GO0008270|F|zinc ion binding | - | - |
| D3 | ***GTTTGT*** | - | - | - |
| D4 | ***ATTAACA*** | - | - | GO0030529|C|ribonucleoprotein complex |
| D5 | ***TGAAG*** | - | - | - |
| D6 | ***TAACC*** | - | GO0006355|P|regulation of transcription, DNA-dependent | - |
| D7 | ***TTGAAAT*** | GO0004713|F|protein-tyrosine kinase activity | - | - |
| D8 | ***AATTG*** | - | - | - |
| D9 | ***CTGCT*** | - | - | - |
| D10 | ***TTTATG*** | - | - | - |
| D11 | ***TGATAAA*** | - | - | - |
| D12 | ***ATGTTT*** | - | GO0001501|P|skeletal development, GO0006955|P|immune response | - |
| D13 | ***TTTCCAA*** | - | - | - |
| D14 | ***AAGTC*** | - | - | - |
| D15 | ***AAAGA*** | - | - | - |
| D16 | ***GTACGT*** | - | - | - |
| D17 | ***GTTAAA*** | - | GO0007049|P|cell cycle, GO0007067|P|mitosis | - |
| D18 | ***GAGCTG*** | GO0003707|F|steroid hormone receptor activity | - | - |
| D19 | ***TCATTTT*** | - | GO0000398|P|nuclear mRNA splicing, via spliceosome | - |
| D20 | ***TGCATG*** | - | GO0006813|P|potassium ion transport, GO0007268|P|synaptic transmission | - |
| D21 | ***TTCTT*** | GO0004872|F|receptor activity, GO0016757|F|transferase activity, transferring glycosyl groups | GO0007155|P|cell adhesion | GO0005634|C|nucleus |
| D22 | ***TTTATC*** | - | - | - |
| D23 | ***ACATTT*** | - | - | - |
| D24 | ***TGCCAGC*** | - | - | - |
| D25 | ***ATAATT*** | - | - | - |
| D26 | ***GTAGG*** | - | GO0009653|P|morphogenesis | - |
| D27 | ***GTATCCT*** | GO0005261|F|cation channel activity, GO0005525|F|GTP binding | GO0006865|P|amino acid transport, GO0006812|P|cation transport | GO0005576|C|extracellular |
| D28 | ***CATTTG*** | - | - | - |
| D29 | ***ACTAAC*** | - | GO0006936|P|muscle contraction | - |
| D30 | ***TTTCAG*** | GO0016853|F|isomerase activity, GO0004295|F|trypsin activity | GO0007517|P|muscle development | GO0005624|C|membrane fraction, GO0015629|C|actin cytoskeleton |
| D31 | ***AATTGA*** | - | - | - |
| D32 | ***TTAGCA*** | - | - | - |
| D33 | ***CAAAT*** | - | GO0006936|P|muscle contraction | - |
| D34 | ***TAATG*** | - | - | - |
| D35 | ***TTTTGAT*** | - | - | - |
| D36 | ***AGAAAT*** | - | - | - |
| D37 | ***TTTCTA*** | - | GO0006813|P|potassium ion transport, GO0006412|P|protein biosynthesis | GO0016021|C|integral to membrane |
| D38 | ***TATTTC*** | - | - | - |
| D39 | ***TAACT*** | GO0003723|F|RNA binding, GO0005201|F|extracellular matrix structural constituent | - | - |
| D40 | ***TGAGG*** | GO0016820|F|hydrolase activity, acting on acid anhydrides, catalyzing transmembrane movement of substances | - | - |
| D41 | ***TAAAAT*** | GO0003779|F|actin binding | - | - |
| D42 | ***TTTATA*** | - | - | - |
| D43 | ***TATCCT*** | - | - | - |
| D44 | ***GTTAGT*** | - | GO0007399|P|neurogenesis, GO0000398|P|nuclear mRNA splicing, via spliceosome, GO0006445|P|regulation of translation, GO0009653|P|morphogenesis | GO0016021|C|integral to membrane |
| D45 | ***TTTACAG*** | - | - | - |
| D46 | ***TATTTG*** | - | - | - |
| D47 | ***GTACTGT*** | - | - | - |
| D48 | ***TTAAG*** | GO0004221|F|ubiquitin thiolesterase activity, GO0004197|F|cysteine-type endopeptidase activity | - | - |
| D49 | ***CATAAA*** | - | - | GO0005887|C|integral to plasma membrane |
| D50 | ***GCATG*** | GO0005515|F|protein binding | - | - |
| D51 | ***TGATTA*** | GO0017111|F|nucleoside-triphosphatase activity | - | - |
| D52 | ***TTTTAAA*** | GO0005198|F|structural molecule activity | - | - |
| D53 | ***CTGACT*** | - | - | - |
| D54 | ***ACTAAT*** | - | - | - |
| D55 | ***GAGTA*** | - | - | - |
| D56 | ***TCTTAA*** | - | - | - |
| D57 | ***TTGGTT*** | - | - | - |
| D58 | ***ATATTT*** | - | GO0000074|P|regulation of cell cycle, GO0006412|P|protein biosynthesis | - |
| D59 | ***AGAGCCA*** | - | - | - |
| D60 | ***TCTTT*** | - | - | - |
| D61 | ***AGTTTT*** | GO0003677|F|DNA binding | GO0000398|P|nuclear mRNA splicing, via spliceosome | GO0005634|C|nucleus |
| D62 | ***GTATTT*** | GO0004872|F|receptor activity, GO0005509|F|calcium ion binding | - | GO0016020|C|membrane, GO0005634|C|nucleus |
| D63 | ***TCAGA*** | - | - | - |
| D64 | ***TAAGT*** | GO0005488|F|binding, GO0003723|F|RNA binding, GO0008270|F|zinc ion binding | GO0007229|P|integrin-mediated signaling pathway, GO0006955|P|immune response, GO0006512|P|ubiquitin cycle | GO0000776|C|kinetochore, GO0005634|C|nucleus, GO0005643|C|nuclear pore |
| D65 | ***AAGCA*** | GO0030145|F|manganese ion binding | - | - |
| D66 | ***TTCACAG*** | - | - | - |
| D67 | ***AGTAA*** | - | GO0006512|P|ubiquitin cycle | - |
| D68 | ***TCTGG*** | GO0003700|F|transcription factor activity | GO0006366|P|transcription from Pol II promoter | GO0005634|C|nucleus |
| D69 | ***AGCTTT*** | GO0008248|F|pre-mRNA splicing factor activity | GO0008380|P|RNA splicing | - |
| D70 | ***TGATTTG*** | - | - | - |
| D71 | ***TTTTGC*** | - | GO0006508|P|proteolysis and peptidolysis, GO0045786|P|negative regulation of cell cycle | - |
| D72 | ***TAGAAA*** | - | - | - |
| D73 | ***GTGAG*** | GO0004872|F|receptor activity, GO0004024|F|alcohol dehydrogenase activity, zinc-dependent, GO0004722|F|protein serine/threonine phosphatase activity, GO0008092|F|cytoskeletal protein binding, GO0003700|F|transcription factor activity, GO0005198|F|structural molecule activity, GO0004263|F|chymotrypsin activity, GO0005245|F|voltage-gated calcium channel activity | GO0006816|P|calcium ion transport, GO0007049|P|cell cycle, GO0007131|P|meiotic recombination, GO0006094|P|gluconeogenesis, GO0016055|P|Wnt receptor signaling pathway, GO0007010|P|cytoskeleton organization and biogenesis, GO0006897|P|endocytosis, GO0006979|P|response to oxidative stress, GO0007165|P|signal transduction, GO0000910|P|cytokinesis | GO0005576|C|extracellular, GO0005794|C|Golgi apparatus, GO0005625|C|soluble fraction, GO0019717|C|synaptosome, GO0045202|C|synapse, GO0005859|C|muscle myosin |
| D74 | ***TTCTGT*** | - | - | - |
| D75 | ***GTAAG*** | GO0003774|F|motor activity, GO0004857|F|enzyme inhibitor activity, GO0004840|F|ubiquitin conjugating enzyme activity | GO0008283|P|cell proliferation, GO0006118|P|electron transport, GO0006364|P|rRNA processing, GO0030155|P|regulation of cell adhesion, GO0006605|P|protein targeting, GO0042110|P|T-cell activation, GO0019538|P|protein metabolism, GO0006512|P|ubiquitin cycle | GO0005743|C|mitochondrial inner membrane, GO0030529|C|ribonucleoprotein complex, GO0005779|C|integral to peroxisomal membrane, GO0030530|C|heterogeneous nuclear ribonucleoprotein complex |
| D76 | ***ATGAAA*** | - | - | - |
| D77 | ***AGAAAA*** | GO0005279|F|amino acid-polyamine transporter activity | GO0007165|P|signal transduction | - |
| D78 | ***TGAGC*** | - | - | - |
| D79 | ***TGGCTT*** | GO0003700|F|transcription factor activity | GO0006512|P|ubiquitin cycle | - |
| D80 | ***TTAATCT*** | - | - | - |
| D81 | ***AATTAT*** | - | - | - |
| D82 | ***TGGAAAT*** | - | - | - |
| D83 | ***CCACAG*** | GO0016491|F|oxidoreductase activity | - | - |
| D84 | ***AAATGA*** | GO0008092|F|cytoskeletal protein binding | - | - |
| D85 | ***GCAAGT*** | - | - | - |
| D86 | ***GTAAAA*** | - | - | - |
| D87 | ***GTCTG*** | - | GO0007268|P|synaptic transmission | - |
| D88 | ***AAATGT*** | - | GO0007417|P|central nervous system development | - |
| D89 | ***TGCAT*** | - | - | - |
| D90 | ***GAGAAA*** | - | - | - |
| D91 | ***TTAGA*** | GO0051082|F|unfolded protein binding | GO0006955|P|immune response | - |
| D92 | ***TTTATAA*** | GO0005488|F|binding | - | - |
| D93 | ***GTTTT*** | GO0005488|F|binding, GO0003676|F|nucleic acid binding | GO0000398|P|nuclear mRNA splicing, via spliceosome | - |
| D94 | ***GCTTGGC*** | - | - | - |
| D95 | ***TAAGC*** | - | - | - |
| D96 | ***GTATG*** | GO0004872|F|receptor activity, GO0004721|F|phosphoprotein phosphatase activity, GO0008248|F|pre-mRNA splicing factor activity, GO0005516|F|calmodulin binding, GO0005509|F|calcium ion binding, GO0000155|F|two-component sensor molecule activity, GO0005215|F|transporter activity | GO0045449|P|regulation of transcription, GO0006281|P|DNA repair, GO0000160|P|two-component signal transduction system (phosphorelay) | GO0005887|C|integral to plasma membrane, GO0005576|C|extracellular |
| D97 | ***AAATT*** | GO0016829|F|lyase activity, GO0016491|F|oxidoreductase activity | GO0008544|P|epidermis development, GO0006512|P|ubiquitin cycle | GO0016021|C|integral to membrane |
| D98 | ***GTAAT*** | GO0005509|F|calcium ion binding | GO0000398|P|nuclear mRNA splicing, via spliceosome | GO0005863|C|striated muscle thick filament, GO0005694|C|chromosome |
| D99 | ***TTCTCT*** | - | - | GO0016020|C|membrane |
| D100 | ***TGAGAA*** | - | GO0006520|P|amino acid metabolism | GO0005634|C|nucleus |
| D101 | ***TTAGTT*** | - | - | - |
| D102 | ***TAAGG*** | - | - | - |
| D103 | ***TGTTTAA*** | - | GO0007186|P|G-protein coupled receptor protein signaling pathway, GO0007417|P|central nervous system development | - |
| D104 | ***GTCAGT*** | - | - | - |
| D105 | ***AGAATT*** | - | - | - |
| D106 | ***TAAATG*** | - | GO0007275|P|development | - |
| D107 | ***AATTCA*** | - | - | - |
| D108 | ***TCCTTT*** | GO0005245|F|voltage-gated calcium channel activity | GO0006898|P|receptor mediated endocytosis | - |
| D109 | ***TAAGA*** | GO0003779|F|actin binding | - | - |
| D110 | ***AAATCA*** | - | - | - |
| D111 | ***TAATTTG*** | GO0003824|F|catalytic activity | - | - |
| D112 | ***GAAATA*** | - | - | - |
| D113 | ***TGGTTT*** | - | - | - |
| D114 | ***TGTTAA*** | - | - | - |
| D115 | ***TGTCT*** | - | - | - |
| D116 | ***GTTGGT*** | - | - | - |
| D117 | ***TGAATT*** | - | - | - |
| D118 | ***AATTTA*** | - | - | - |
| D119 | ***TATGT*** | - | GO0015031|P|protein transport | - |
| D120 | ***GCATTT*** | - | - | - |
| D121 | ***AAGTA*** | GO0005509|F|calcium ion binding, GO0004840|F|ubiquitin conjugating enzyme activity | - | - |
| D122 | ***GCTTCT*** | - | - | - |
| D123 | ***TTCTAA*** | - | - | - |
| D124 | ***GTTTCT*** | - | - | - |
| D125 | ***AGATTT*** | - | GO0008380|P|RNA splicing | - |
| D126 | ***GAAAAT*** | - | - | - |
| D127 | ***TGCTAA*** | - | - | - |
| D128 | ***AAGCT*** | - | - | - |
| D129 | ***CTTTGCT*** | - | - | - |
| D130 | ***TCTGA*** | - | - | - |
| D131 | ***TTTCTC*** | - | - | - |
| D132 | ***TTTATTC*** | - | - | - |
| D133 | ***TTTGCC*** | - | - | - |
| D134 | ***TGAAAG*** | GO0004842|F|ubiquitin-protein ligase activity | - | - |
| D135 | ***TGTTCT*** | - | - | - |
| D136 | ***CTTTT*** | GO0004872|F|receptor activity | GO0000398|P|nuclear mRNA splicing, via spliceosome, GO0006955|P|immune response, GO0008380|P|RNA splicing | - |
| D137 | ***TTTTCTG*** | - | - | - |
| D138 | ***TGAGT*** | GO0008083|F|growth factor activity | GO0008624|P|induction of apoptosis by extracellular signals, GO0046839|P|phospholipid dephosphorylation | GO0005624|C|membrane fraction, GO0005886|C|plasma membrane, GO0008021|C|synaptic vesicle |
| D139 | ***TTGCAG*** | - | - | GO0005634|C|nucleus |
| D140 | ***TAATA*** | GO0008248|F|pre-mRNA splicing factor activity, GO0004221|F|ubiquitin thiolesterase activity, GO0005070|F|SH3/SH2 adaptor protein activity | GO0000398|P|nuclear mRNA splicing, via spliceosome, GO0006511|P|ubiquitin-dependent protein catabolism | GO0005887|C|integral to plasma membrane |
| D141 | ***AGTAT*** | GO0016301|F|kinase activity, GO0003723|F|RNA binding, GO0004842|F|ubiquitin-protein ligase activity | - | - |
| D142 | ***ATTCT*** | - | - | - |
| D143 | ***TGCCTTT*** | - | - | - |
| D144 | ***ATCAAA*** | - | - | - |
| D145 | ***GAGTG*** | - | - | - |
| D146 | ***TAGGT*** | GO0019992|F|diacylglycerol binding | - | - |
| D147 | ***CTTTA*** | - | - | - |
| D148 | ***TTTAG*** | GO0005178|F|integrin binding | - | GO0016021|C|integral to membrane, GO0005654|C|nucleoplasm |
| D149 | ***TGATTTT*** | - | - | - |
| D150 | ***TTTCAT*** | GO0003676|F|nucleic acid binding | GO0000398|P|nuclear mRNA splicing, via spliceosome, GO0007275|P|development | GO0005764|C|lysosome |
| D151 | ***CTTTCA*** | - | GO0006333|P|chromatin assembly or disassembly | GO0000785|C|chromatin |
| D152 | ***AAGAT*** | - | - | - |
| D153 | ***TGCTT*** | - | - | - |
| D154 | ***GTGGGT*** | GO0005515|F|protein binding | - | - |
| D155 | ***GTAAAG*** | - | - | - |
| D156 | ***CTGAA*** | - | - | - |
| D157 | ***TCTGC*** | - | - | - |
| D158 | ***CTAAA*** | - | - | - |
| U1 | ***GTTTGT*** | GO0003723|F|RNA binding, GO0004674|F|protein serine/threonine kinase activity | - | GO0005634|C|nucleus |
| U2 | ***TCTCC*** | GO0004221|F|ubiquitin thiolesterase activity, GO0005249|F|voltage-gated potassium channel activity | GO0006869|P|lipid transport, GO0006813|P|potassium ion transport, GO0006511|P|ubiquitin-dependent protein catabolism, GO0008203|P|cholesterol metabolism, GO0008284|P|positive regulation of cell proliferation | - |
| U3 | ***GATTTT*** | - | - | - |
| U4 | ***TTTTTC*** | GO0005488|F|binding, GO0004842|F|ubiquitin-protein ligase activity, GO0005215|F|transporter activity, GO0005529|F|sugar binding, GO0016491|F|oxidoreductase activity, GO0016563|F|transcriptional activator activity | GO0008544|P|epidermis development, GO0006812|P|cation transport, GO0007155|P|cell adhesion, GO0006814|P|sodium ion transport, GO0015031|P|protein transport, GO0006629|P|lipid metabolism, GO0007229|P|integrin-mediated signaling pathway, GO0045786|P|negative regulation of cell cycle, GO0006306|P|DNA methylation, GO0006470|P|protein amino acid dephosphorylation, GO0006333|P|chromatin assembly or disassembly | GO0016020|C|membrane, GO0005667|C|transcription factor complex, GO0005576|C|extracellular, GO0005578|C|extracellular matrix (sensu Metazoa), GO0005839|C|proteasome core complex (sensu Eukaryota), GO0005654|C|nucleoplasm |
| U5 | ***TAACC*** | - | - | - |
| U6 | ***TTGAAAT*** | - | - | - |
| U7 | ***AAGCCA*** | - | - | - |
| U8 | ***AATTG*** | - | - | - |
| U9 | ***CTGCT*** | - | - | GO0000151|C|ubiquitin ligase complex |
| U10 | ***TTTATG*** | - | - | GO0005634|C|nucleus |
| U11 | ***TTCACA*** | - | GO0006508|P|proteolysis and peptidolysis | - |
| U12 | ***TGATAA*** | - | - | - |
| U13 | ***ATGTTT*** | - | - | - |
| U14 | ***TCCAG*** | GO0008083|F|growth factor activity, GO0005245|F|voltage-gated calcium channel activity | GO0006816|P|calcium ion transport, GO0006869|P|lipid transport, GO0009653|P|morphogenesis | - |
| U15 | ***TTTCCAA*** | - | GO0008284|P|positive regulation of cell proliferation, GO0006412|P|protein biosynthesis | GO0005622|C|intracellular, GO0005615|C|extracellular space |
| U16 | ***TTATTTC*** | - | - | - |
| U17 | ***TGTGTT*** | - | - | - |
| U18 | ***TCTTG*** | - | GO0006898|P|receptor mediated endocytosis, GO0007160|P|cell-matrix adhesion | - |
| U19 | ***TTGTAA*** | - | GO0006915|P|apoptosis | - |
| U20 | ***CTTGAC*** | - | - | - |
| U21 | ***TTAAAAC*** | - | - | - |
| U22 | ***CTAAC*** | GO0003824|F|catalytic activity | - | GO0015629|C|actin cytoskeleton |
| U23 | ***AAAGCT*** | - | - | - |
| U24 | ***TCTTC*** | GO0030528|F|transcription regulator activity | - | - |
| U25 | ***TGCATG*** | GO0003779|F|actin binding | - | - |
| U26 | ***TTCTT*** | GO0004295|F|trypsin activity | GO0006629|P|lipid metabolism, GO0007229|P|integrin-mediated signaling pathway | GO0005624|C|membrane fraction |
| U27 | ***ACATTT*** | - | GO0007417|P|central nervous system development | - |
| U28 | ***TTTATC*** | - | - | - |
| U29 | ***ATTTTCT*** | GO0003677|F|DNA binding | GO0008544|P|epidermis development | - |
| U30 | ***GCTGACC*** | - | - | - |
| U31 | ***ATAATT*** | GO0005200|F|structural constituent of cytoskeleton | - | - |
| U32 | ***AACAG*** | - | - | - |
| U33 | ***CATTTG*** | - | - | - |
| U34 | ***TTTCAG*** | GO0030374|F|ligand-dependent nuclear receptor transcription coactivator activity | GO0006310|P|DNA recombination | GO0000119|C|mediator complex, GO0005819|C|spindle, GO0008021|C|synaptic vesicle |
| U35 | ***TTAGCA*** | - | - | - |
| U36 | ***TTGCCT*** | - | - | - |
| U37 | ***CAAAT*** | - | - | - |
| U38 | ***TTTAAC*** | - | - | - |
| U39 | ***TAATG*** | GO0004721|F|phosphoprotein phosphatase activity | - | - |
| U40 | ***AGAAAT*** | - | - | - |
| U41 | ***TTTTGAT*** | - | - | - |
| U42 | ***TAACT*** | GO0004872|F|receptor activity | - | GO0005615|C|extracellular space |
| U43 | ***TAAAAT*** | GO0015293|F|symporter activity | - | - |
| U44 | ***AATTACA*** | - | - | - |
| U45 | ***TTCAAAA*** | - | - | - |
| U46 | ***TTTATA*** | GO0005488|F|binding, GO0017111|F|nucleoside-triphosphatase activity | GO0006511|P|ubiquitin-dependent protein catabolism, GO0006512|P|ubiquitin cycle | GO0005576|C|extracellular |
| U47 | ***CTTGTC*** | - | - | GO0005634|C|nucleus |
| U48 | ***TTTACAG*** | GO0016491|F|oxidoreductase activity | GO0007169|P|transmembrane receptor protein tyrosine kinase signaling pathway | GO0005634|C|nucleus |
| U49 | ***TGGATTT*** | - | - | - |
| U50 | ***TTGCATT*** | - | - | - |
| U51 | ***TTAAG*** | GO0003676|F|nucleic acid binding, GO0005516|F|calmodulin binding, GO0016829|F|lyase activity | GO0007155|P|cell adhesion, GO0006886|P|intracellular protein transport | GO0005624|C|membrane fraction |
| U52 | ***TTGGT*** | GO0003676|F|nucleic acid binding | GO0000398|P|nuclear mRNA splicing, via spliceosome | - |
| U53 | ***CATAAA*** | - | GO0006470|P|protein amino acid dephosphorylation | - |
| U54 | ***TGATTA*** | - | - | - |
| U55 | ***GCTTTGC*** | - | - | - |
| U56 | ***ATTAG*** | GO0004197|F|cysteine-type endopeptidase activity | GO0007010|P|cytoskeleton organization and biogenesis | GO0005604|C|basement membrane |
| U57 | ***TTTTAAA*** | GO0004872|F|receptor activity, GO0003723|F|RNA binding | GO0006512|P|ubiquitin cycle | GO0005634|C|nucleus |
| U58 | ***ACTAAT*** | GO0004221|F|ubiquitin thiolesterase activity | - | - |
| U59 | ***CTGACT*** | - | - | - |
| U60 | ***ATATTT*** | GO0005178|F|integrin binding | GO0000074|P|regulation of cell cycle | - |
| U61 | ***CATTTA*** | - | - | - |
| U62 | ***AAATCT*** | GO0003755|F|peptidyl-prolyl cis-trans isomerase activity | - | - |
| U63 | ***TTTTGGC*** | - | - | - |
| U64 | ***TCTTT*** | GO0008248|F|pre-mRNA splicing factor activity, GO0004497|F|monooxygenase activity, GO0019825|F|oxygen binding | GO0006470|P|protein amino acid dephosphorylation | GO0005863|C|striated muscle thick filament, GO0005634|C|nucleus, GO0005643|C|nuclear pore |
| U65 | ***TTATTGA*** | - | - | - |
| U66 | ***TCAGA*** | - | GO0007399|P|neurogenesis | - |
| U67 | ***TAAGT*** | GO0019992|F|diacylglycerol binding | - | - |
| U68 | ***CTCTG*** | GO0003677|F|DNA binding, GO0005215|F|transporter activity, GO0005234|F|glutamate-gated ion channel activity, GO0005262|F|calcium channel activity | GO0007268|P|synaptic transmission, GO0006886|P|intracellular protein transport | GO0016021|C|integral to membrane, GO0016020|C|membrane |
| U69 | ***TCTGG*** | - | - | - |
| U70 | ***AATTC*** | - | - | - |
| U71 | ***TTTTCC*** | - | GO0006118|P|electron transport | GO0005634|C|nucleus |
| U72 | ***TTTTGC*** | - | GO0000398|P|nuclear mRNA splicing, via spliceosome, GO0008380|P|RNA splicing | - |
| U73 | ***GTGAG*** | GO0004872|F|receptor activity, GO0003735|F|structural constituent of ribosome, GO0005125|F|cytokine activity | GO0006890|P|retrograde transport, Golgi to ER, GO0007126|P|meiosis, GO0006955|P|immune response, GO0007601|P|visual perception, GO0007507|P|heart development, GO0006629|P|lipid metabolism, GO0006952|P|defense response, GO0007267|P|cell-cell signaling, GO0006350|P|transcription, GO0006298|P|mismatch repair, GO0006839|P|mitochondrial transport, GO0006096|P|glycolysis | - |
| U74 | ***TCCATTT*** | - | - | - |
| U75 | ***AATTTT*** | - | - | - |
| U76 | ***CTTGATT*** | - | - | - |
| U77 | ***GTAAG*** | GO0003735|F|structural constituent of ribosome | GO0016043|P|cell organization and biogenesis | GO0016021|C|integral to membrane, GO0005737|C|cytoplasm, GO0005634|C|nucleus |
| U78 | ***ATGAAA*** | GO0004872|F|receptor activity | - | - |
| U79 | ***AGAAAA*** | - | - | - |
| U80 | ***TGGCTT*** | - | - | - |
| U81 | ***CTCAG*** | GO0005516|F|calmodulin binding | GO0006512|P|ubiquitin cycle | - |
| U82 | ***TGGAAAT*** | - | - | - |
| U83 | ***AATTAT*** | - | - | - |
| U84 | ***AATAAT*** | GO0004842|F|ubiquitin-protein ligase activity | GO0016567|P|protein ubiquitination | - |
| U85 | ***TCCTAG*** | - | - | - |
| U86 | ***CCACAG*** | - | GO0007399|P|neurogenesis, GO0006955|P|immune response | GO0005624|C|membrane fraction |
| U87 | ***TCATTTC*** | - | - | - |
| U88 | ***AAAGCA*** | GO0005247|F|voltage-gated chloride channel activity, GO0017111|F|nucleoside-triphosphatase activity | GO0006813|P|potassium ion transport, GO0007067|P|mitosis, GO0045786|P|negative regulation of cell cycle | GO0005625|C|soluble fraction |
| U89 | ***AAATGA*** | - | - | - |
| U90 | ***TTTATAG*** | GO0003677|F|DNA binding | - | GO0005634|C|nucleus |
| U91 | ***ATTAAAT*** | - | - | - |
| U92 | ***CCTGCAG*** | - | GO0007267|P|cell-cell signaling, GO0006512|P|ubiquitin cycle | - |
| U93 | ***TTACAG*** | GO0008026|F|ATP-dependent helicase activity | - | - |
| U94 | ***AAATGT*** | - | - | GO0005634|C|nucleus |
| U95 | ***TGCAT*** | GO0016491|F|oxidoreductase activity | - | - |
| U96 | ***CTTCT*** | - | - | - |
| U97 | ***TTAGAA*** | - | GO0006928|P|cell motility | GO0005863|C|striated muscle thick filament |
| U98 | ***TGTTTC*** | GO0003700|F|transcription factor activity, GO0008270|F|zinc ion binding | GO0006355|P|regulation of transcription, DNA-dependent | - |
| U99 | ***TTTAC*** | - | GO0006260|P|DNA replication | - |
| U100 | ***GTTTT*** | GO0003700|F|transcription factor activity, GO0005509|F|calcium ion binding, GO0004263|F|chymotrypsin activity, GO0004295|F|trypsin activity, GO0017111|F|nucleoside-triphosphatase activity, GO0003702|F|RNA polymerase II transcription factor activity | GO0000398|P|nuclear mRNA splicing, via spliceosome, GO0006811|P|ion transport, GO0007155|P|cell adhesion | GO0005581|C|collagen |
| U101 | ***CTTCCA*** | GO0004222|F|metalloendopeptidase activity | - | - |
| U102 | ***TTCTAG*** | GO0004383|F|guanylate cyclase activity | - | - |
| U103 | ***AAATT*** | GO0004888|F|transmembrane receptor activity, GO0004197|F|cysteine-type endopeptidase activity, GO0003779|F|actin binding | GO0007517|P|muscle development, GO0006936|P|muscle contraction | GO0005625|C|soluble fraction |
| U104 | ***TTAAAC*** | - | - | - |
| U105 | ***TGAGAA*** | - | - | - |
| U106 | ***TTTGTAG*** | GO0003723|F|RNA binding | - | - |
| U107 | ***GTCAGT*** | - | - | - |
| U108 | ***TAAGA*** | - | - | - |
| U109 | ***AAATCA*** | - | - | - |
| U110 | ***TGTTGA*** | - | - | - |
| U111 | ***CTTGC*** | GO0003700|F|transcription factor activity | - | - |
| U112 | ***TAATTTG*** | - | GO0006817|P|phosphate transport, GO0007160|P|cell-matrix adhesion | GO0005578|C|extracellular matrix (sensu Metazoa) |
| U113 | ***CCTCT*** | - | - | GO0005887|C|integral to plasma membrane |
| U114 | ***TGGTTT*** | - | - | - |
| U115 | ***TGATTTC*** | - | - | - |
| U116 | ***TGTTAA*** | GO0003677|F|DNA binding | GO0007605|P|perception of sound, GO0006118|P|electron transport | GO0016020|C|membrane, GO0005576|C|extracellular, GO0005634|C|nucleus |
| U117 | ***TGTGTC*** | - | - | - |
| U118 | ***TCTCT*** | GO0051082|F|unfolded protein binding, GO0003700|F|transcription factor activity, GO0003899|F|DNA-directed RNA polymerase activity, GO0016853|F|isomerase activity | GO0007399|P|neurogenesis, GO0007268|P|synaptic transmission, GO0007565|P|pregnancy | - |
| U119 | ***TTAACA*** | GO0016491|F|oxidoreductase activity | - | - |
| U120 | ***TTTGGT*** | - | GO0000398|P|nuclear mRNA splicing, via spliceosome, GO0006260|P|DNA replication | - |
| U121 | ***TGTCT*** | GO0003713|F|transcription coactivator activity | - | - |
| U122 | ***TTCCTT*** | GO0000166|F|nucleotide binding | - | GO0005891|C|voltage-gated calcium channel complex |
| U123 | ***TGAATT*** | - | GO0045786|P|negative regulation of cell cycle | - |
| U124 | ***AATTTA*** | - | - | - |
| U125 | ***GTTTCT*** | - | - | - |
| U126 | ***TGCTAA*** | - | - | GO0005887|C|integral to plasma membrane |
| U127 | ***AGATTT*** | - | - | - |
| U128 | ***GAAAAT*** | - | - | GO0005886|C|plasma membrane |
| U129 | ***GTTTAAT*** | - | - | GO0005634|C|nucleus |
| U130 | ***TTTGACT*** | - | - | - |
| U131 | ***TCTGA*** | GO0003735|F|structural constituent of ribosome | GO0000398|P|nuclear mRNA splicing, via spliceosome | - |
| U132 | ***TCTGTT*** | GO0003700|F|transcription factor activity, GO0004527|F|exonuclease activity | GO0006812|P|cation transport | - |
| U133 | ***TTTATTC*** | - | - | - |
| U134 | ***TGAAAG*** | - | - | - |
| U135 | ***TGTTCT*** | GO0003723|F|RNA binding, GO0008248|F|pre-mRNA splicing factor activity, GO0005198|F|structural molecule activity | GO0006396|P|RNA processing, GO0007420|P|brain development, GO0006915|P|apoptosis | - |
| U136 | ***CTTTT*** | - | - | - |
| U137 | ***ATTTGT*** | - | GO0000398|P|nuclear mRNA splicing, via spliceosome | GO0016021|C|integral to membrane |
| U138 | ***TGAGT*** | - | - | - |
| U139 | ***CCCCAG*** | GO0003707|F|steroid hormone receptor activity, GO0008083|F|growth factor activity, GO0005509|F|calcium ion binding, GO0003684|F|damaged DNA binding | GO0007283|P|spermatogenesis, GO0016481|P|negative regulation of transcription, GO0006511|P|ubiquitin-dependent protein catabolism | - |
| U140 | ***TTGCAG*** | - | - | - |
| U141 | ***CTGAT*** | GO0003735|F|structural constituent of ribosome | - | GO0005840|C|ribosome |
| U142 | ***TAATA*** | - | - | - |
| U143 | ***TCTTA*** | GO0003723|F|RNA binding, GO0003700|F|transcription factor activity | - | GO0005634|C|nucleus |
| U144 | ***ATTCT*** | - | - | - |
| U145 | ***ATCAAA*** | - | - | - |
| U146 | ***CTTTA*** | - | GO0045786|P|negative regulation of cell cycle, GO0006355|P|regulation of transcription, DNA-dependent | - |
| U147 | ***TTTAG*** | GO0003723|F|RNA binding, GO0003774|F|motor activity, GO0004295|F|trypsin activity, GO0003779|F|actin binding, GO0004840|F|ubiquitin conjugating enzyme activity | GO0006816|P|calcium ion transport, GO0045449|P|regulation of transcription, GO0000398|P|nuclear mRNA splicing, via spliceosome, GO0007155|P|cell adhesion, GO0007169|P|transmembrane receptor protein tyrosine kinase signaling pathway, GO0006260|P|DNA replication | GO0005576|C|extracellular, GO0000785|C|chromatin, GO0005863|C|striated muscle thick filament, GO0005859|C|muscle myosin |
| U148 | ***TTGCTG*** | - | - | - |
| U149 | ***TTTCAT*** | GO0005200|F|structural constituent of cytoskeleton, GO0003725|F|double-stranded RNA binding, GO0016829|F|lyase activity, GO0030528|F|transcription regulator activity | - | - |
| U150 | ***CTTTCA*** | - | GO0008544|P|epidermis development | - |
| U151 | ***TTCTC*** | GO0003707|F|steroid hormone receptor activity | - | - |
| U152 | ***TGCTT*** | GO0004263|F|chymotrypsin activity | - | GO0005576|C|extracellular |
| U153 | ***TCTGC*** | - | - | - |
| U154 | ***CTGAA*** | - | - | - |
| U155 | ***GTAGGT*** | GO0005488|F|binding, GO0003723|F|RNA binding | - | - |
| U156 | ***CTAAA*** | - | GO0008544|P|epidermis development | GO0005625|C|soluble fraction |
