# Supplementary material for: Discovery and Analysis of Evolutionarily Conserved Intronic Splicing Regulatory Elements
Source: PLoS Genet. 2007 May 25;3(5):e85. doi: 10.1371/journal.pgen.0030085 (PMC1877881; doi:10.1371/journal.pgen.0030085)
Supplement: Table S5 — Shown are tissue-expression biases of genes containing (A) downstream ISRE-proximal exons and (B) upstream ISRE-proximal exons. (145 KB PDF) [file pgen.0030085.st005.pdf]

Table S5. Tissue expression biases of genes containing downstream ISRE-proximal exons and upstream ISRE-proximal exons.

Index	Parent	Significantly depleted in tissues (p<0.001)	Significantly enriched in tissues (p<0.001)
D1	***GTAAC***	-	-
D2	***AAGTGT***	-	-
D3	***GTTTGT***	Whole Brain	BM-CD34+
D4	***ATTAACA***	-	-
D5	***TGAAG***	Lung, Colorectal Adenocarcinoma	BM-CD34+
D6	***TAACC***	-	-
D7	***TTGAAAT***	-	-
D8	***AATTG***	Lung, Testis, Liver	BM-CD34+
D9	***CTGCT***	-	Temporal Lobe
D10	***TTTATG***	Lymphoma Burkitts Raji	-
D11	***TGATAAA***	-	Salivary Gland
D12	***ATGTTT***	Heart, PB-CD14+ Monocytes, Lymphoma Burkitts Raji, Liver	Superior Cervical Ganglion
D13	***TTTCCAA***	-	-
D14	***AAGTC***	-	-
D15	***AAAGA***	Heart, Lymphoma Burkitts Raji, Liver	Occipital Lobe, DRG, Appendix, Superior Cervical Ganglion, Trigeminal Ganglion
D16	***GTACGT***	-	Caudate Nucleus, Whole Brain
D17	***GTTAAA***	-	-
D18	***GAGCTG***	-	-
D19	***TCATTTT***	Heart	-
D20	***TGCATG***	Liver, Lymphoma Burkitts Daudi	Occipital Lobe, Fetal Brain, Parietal Lobe, Hypothalamus, Thalamus, Whole Brain, Cingulate Cortex, Medulla Oblongata, Temporal Lobe, Cerebellum Peduncles, Prefrontal Cortex, Amygdala, Cerebellum, Globus Pallidus, Subthalamic Nucleus

D21	***TTCTT***	Heart, Lung, Lymphoma Burkitts Raji, Liver, Colorectal Adenocarcinoma, Prostate	BM-CD34+, 721B Lymphoblasts, BM-CD105+ Endothelial, Ciliary Ganglion
D22	***TTTATC***	Lung, Liver	-
D23	***ACATTT***	-	-
D24	***TGCCAGC***	-	-
D25	***ATAATT***	Lung, Heart, Lymphoma Burkitts Raji	Atrioventricular Node, Appendix, Testis Interstitial, Trigeminal Ganglion, Testis Seminiferous Tubule, Ovary, Testis Germ Cell, Testis Leydig Cell
D26	***GTAGG***	-	Caudate Nucleus, Cingulate Cortex
D27	***GTATCCT***	Placenta	721B Lymphoblasts
D28	***CATTTG***	-	-
D29	***ACTAAC***	-	Prefrontal Cortex
D30	***TTTCAG***	Heart, Lung, Tonsil, Liver, Colorectal Adenocarcinoma	BM-CD34+, Superior Cervical Ganglion, 721B Lymphoblasts
D31	***AATTGA***	-	-
D32	***TTAGCA***	-	-
D33	***CAAAT***	Heart, Lung, PB-CD56+ NK Cells	Occipital Lobe, Superior Cervical Ganglion, Fetal Liver, Globus Pallidus, Pituitary, Trigeminal Ganglion
D34	***TAATG***	Lung, Heart	Appendix, Superior Cervical Ganglion, Ciliary Ganglion
D35	***TTTTGAT***	-	-
D36	***AGAAAT***	Heart, Testis, Lymphoma Burkitts Raji, Colorectal Adenocarcinoma	-
D37	***TTTCTA***	Heart, Lung, Cerebellum, Colorectal	BM-CD34+, Testis Germ Cell, Testis

		Adenocarcinoma	Interstitial
D38	***TATTTC***	Lung	-
D39	***TAACT***	Lung, Heart, Liver	Appendix
D40	***TGAGG***	-	-
D41	***TAAAAT***	Heart, Lung, Liver, Bone Marrow, Colorectal Adenocarcinoma	BM-CD34+, Appendix, Testis Interstitial, Superior Cervical Ganglion, BM-CD105+ Endothelial, Leukemia Promyelocytic (hl60), Testis Seminiferous Tubule
D42	***TTTATA***	Lung, Heart, Liver	Adipocyte, Trigeminal Ganglion
D43	***TATCCT***	Lung	-
D44	***GTTAGT***	Lung, Liver, Adrenal Gland, Cerebellum	Testis Germ Cell, PB-CD19+ B-cells, BM-CD34+, 721B Lymphoblasts, BM-CD105+ Endothelial
D45	***TTTACAG***	-	-
D46	***TATTTG***	Heart	-
D47	***GTACTGT***	-	-
D48	***TTAAG***	Lung, Colorectal Adenocarcinoma	PB-CD19+ B-cells, BM-CD34+
D49	***CATAAA***	-	Occipital Lobe, Appendix, Trigeminal Ganglion
D50	***GCATG***	Adrenal Gland, Testis Interstitial, PB-CD8 T-cells, BM-CD105+ Endothelial, Testis Seminiferous Tubule	Cerebellum Peduncles, Prefrontal Cortex, Amygdala, Fetal Brain, Occipital Lobe, Parietal Lobe, Cingulate Cortex, Medulla Oblongata
D51	***TGATTA***	Lung	-
D52	***TTTTAAA***	Heart, Lung, Liver, Adrenal Cortex	BM-CD34+, PB-CD56+ NK Cells, PB-BDCA4+ Dentritic Cells
D53	***CTGACT***	-	-
D54	***ACTAAT***	Placenta	-
D55	***GAGTA***	-	-
D56	***TCTTAA***	Heart, Lung, Liver, Caudate	BM-CD34+, Superior

		Nucleus	Cervical Ganglion
D57	***TTGGTT***	Heart	-
D58	***ATATTT***	Lung, Heart, Lymphoma Burkitts Raji, PB-CD14+ Monocytes, Liver, Whole Brain	Testis Germ Cell, Skin, Testis Leydig Cell, Superior Cervical Ganglion, BM-CD105+ Endothelial
D59	***AGAGCCA***	-	-
D60	***TCTTT***	Heart, Lung, Whole Brain	BM-CD34+, Lymphoma Burkitts Daudi, 721B Lymphoblasts, Salivary Gland
D61	***AGTTTT***	Lung, Heart	PB-CD19+ B-cells, BM-CD34+, PB-CD8 T-cells, 721B Lymphoblasts, BM-CD105+ Endothelial
D62	***GTATTT***	Lung, Heart, Lymphoma Burkitts Raji, Liver, Adrenal Gland, Kidney, Adipocyte	BM-CD34+, BM-CD105+ Endothelial
D63	***TCAGA***	BM-CD34+, PB-CD56+ NK Cells, PB-BDCA4+ Dentritic Cells	-
D64	***TAAGT***	Lung, Heart, Lymphoma Burkitts Raji, PB-CD14+ Monocytes, Liver, Kidney, Colorectal Adenocarcinoma	PB-CD19+ B-cells, Occipital Lobe, Fetal Brain, BM-CD34+, Parietal Lobe, Superior Cervical Ganglion, 721B Lymphoblasts
D65	***AAGCA***	-	-
D66	***TTCACAG***	-	Kidney
D67	***AGTAA***	Heart	-
D68	***TCTGG***	-	Subthalamic Nucleus
D69	***AGCTTT***	Lung, Placenta	-
D70	***TGATTTG***	-	-
D71	***TTTTGC***	PB-CD14+ Monocytes	-
D72	***TAGAAA***	Heart, Lung, PB-CD14+ Monocytes, Lymphoma Burkitts Raji, Liver	BM-CD34+

D73	***GTGAG***	Appendix, Testis Interstitial, 721B Lymphoblasts, PB-CD8 T-cells, BM-CD105+ Endothelial, Leukemia Promyelocytic (hl60), Testis Seminiferous Tubule, Trigeminal Ganglion, PB-CD19+ B-cells, Testis Germ Cell, BM-CD34+, Testis Leydig Cell, PB-CD4+ T-cells, Superior Cervical Ganglion, Ciliary Ganglion	PB-CD14+ Monocytes, Lymphoma Burkitts Raji, Kidney, Colorectal Adenocarcinoma, Thalamus, Whole Brain, Cingulate Cortex, Lung, Heart, Pancreas, Liver, Cerebellum, Placenta, Cardiac Myocytes
D74	***TTCTGT***	-	-
D75	***GTAAG***	Heart, Lung, Lymphoma Burkitts Raji, Liver, Adrenal Gland, Kidney	Occipital Lobe, Testis Interstitial, 721B Lymphoblasts, BM-CD105+ Endothelial, Bronchial Epithelial Cells, Leukemia Promyelocytic (hl60), Testis Germ Cell, BM-CD34+, Testis Leydig Cell, Superior Cervical Ganglion, PB-CD56+ NK Cells, Adipocyte
D76	***ATGAAA***	Lung, Heart, PB-CD4+ T-cells, PB-CD8 T-cells	Parietal Lobe, Superior Cervical Ganglion, Skeletal Muscle, Uterus Corpus, Trigeminal Ganglion
D77	***AGAAAA***	Lymphoma Burkitts Raji	-
D78	***TGAGC***	-	Skeletal Muscle
D79	***TGGCTT***	-	-
D80	***TTAATCT***	Heart	-
D81	***AATTAT***	Lung, Heart,	-

		Cerebellum Peduncles, Lymphoma Burkitts Raji, Liver	
D82	***TGGAAAT***	-	-
D83	***CCACAG***	-	Heart
D84	***AAATGA***	-	-
D85	***GCAAGT***	721B Lymphoblasts	Cerebellum Peduncles
D86	***GTAAAA***	Heart, Lung, Liver	Occipital Lobe, Testis Germ Cell, Testis Interstitial, Testis Seminiferous Tubule
D87	***GTCTG***	-	Lung, Cingulate Cortex
D88	***AAATGT***	Lung, Heart, Liver	-
D89	***TGCAT***	Lung	Superior Cervical Ganglion
D90	***GAGAAA***	-	-
D91	***TTAGA***	Lung, Heart	BM-CD34+, Lymphoma Burkitts Daudi
D92	***TTTATAA***	Lung, Cardiac Myocytes	-
D93	***GTTTT***	Heart, Lung, Liver, Cerebellum	Occipital Lobe, BM-CD34+, Bronchial Epithelial Cells
D94	***GCTTGGC***	-	Cerebellum, Cingulate Cortex
D95	***TAAGC***	-	-
D96	***GTATG***	Lung, Heart, Liver, Colorectal Adenocarcinoma, Cardiac Myocytes	Testis Germ Cell, BM-CD34+, Appendix, Testis Leydig Cell, PB-CD56+ NK Cells, 721B Lymphoblasts, BM-CD105+ Endothelial
D97	***AAATT***	Lymphoma Burkitts Raji, Colorectal Adenocarcinoma, Lung, Heart, Testis, Liver, Adrenal Gland, Placenta, Cerebellum	Parietal Lobe, Appendix, Testis Interstitial, 721B Lymphoblasts, BM-CD105+ Endothelial, Leukemia Promyelocytic (hl60), Trigeminal Ganglion, Testis

			Germ Cell, BM-CD34+, Superior Cervical Ganglion
D98	***GTAAT***	Lung, Heart, Lymphoma Burkitts Raji, Liver, Colorectal Adenocarcinoma, Placenta, Cerebellum	PB-CD19+ B-cells, BM-CD34+, Appendix, Superior Cervical Ganglion, 721B Lymphoblasts, PB-CD56+ NK Cells, BM-CD105+ Endothelial, Leukemia Promyelocytic (hl60), Trigeminal Ganglion
D99	***TTCTCT***	BM-CD34+, 721B Lymphoblasts, BM-CD105+ Endothelial	-
D100	***TGAGAA***	Lung, Colorectal Adenocarcinoma	Appendix, Superior Cervical Ganglion
D101	***TTAGTT***	-	PB-CD56+ NK Cells
D102	***TAAGG***	Lung, Heart	-
D103	***TGTTTAA***	-	Occipital Lobe
D104	***GTCAGT***	-	Globus Pallidus
D105	***AGAATT***	Lung, Heart, Lymphoma Burkitts Raji, Liver, Placenta	BM-CD34+
D106	***TAAATG***	Heart, Lung, Cerebellum Peduncles, Lymphoma Burkitts Raji, Liver	-
D107	***AATTCA***	Heart	Parietal Lobe
D108	***TCCTTT***	-	-
D109	***TAAGA***	Lung, Liver	BM-CD34+
D110	***AAATCA***	-	Appendix, Trigeminal Ganglion
D111	***TAATTTG***	Heart, Placenta	BM-CD34+, BM-CD105+ Endothelial
D112	***GAAATA***	-	Testis Germ Cell
D113	***TGGTTT***	-	-
D114	***TGTTAA***	Lung	BM-CD34+
D115	***TGTCT***	-	-
D116	***GTTGGT***	-	-
D117	***TGAATT***	Lung, Heart	PB-CD19+ B-cells
D118	***AATTTA***	-	Trigeminal

			Ganglion
D119	***TATGT***	Liver	Amygdala, Occipital Lobe
D120	***GCATTT***	Lung	-
D121	***AAGTA***	Lung, Heart, Liver, Cerebellum	BM-CD34+, 721B Lymphoblasts, BM-CD105+ Endothelial
D122	***GCTTCT***	-	-
D123	***TTCTAA***	Lung, Heart, PB-CD14+ Monocytes, Bone Marrow, Colorectal Adenocarcinoma, Whole Brain	Testis Germ Cell, Occipital Lobe, Testis Leydig Cell
D124	***GTTTCT***	Heart	PB-CD56+ NK Cells
D125	***AGATTT***	Lung, Heart, Liver	Appendix, Superior Cervical Ganglion
D126	***GAAAAT***	-	-
D127	***TGCTAA***	-	Occipital Lobe
D128	***AAGCT***	Colorectal Adenocarcinoma	-
D129	***CTTTGCT***	-	-
D130	***TCTGA***	-	-
D131	***TTTCTC***	-	-
D132	***TTTATTC***	-	-
D133	***TTTGCC***	-	-
D134	***TGAAAG***	-	-
D135	***TGTTCT***	-	-
D136	***CTTTT***	Heart, Lung, Liver, Bone Marrow, Tongue, Whole Brain	BM-CD34+, 721B Lymphoblasts
D137	***TTTTCTG***	Lung	Leukemia Promyelocytic (hl60)
D138	***TGAGT***	Testis Germ Cell, BM-CD34+, Testis Leydig Cell, Testis Interstitial, 721B Lymphoblasts, Leukemia Promyelocytic (hl60), Testis Seminiferous Tubule	PB-CD14+ Monocytes, Colorectal Adenocarcinoma, Whole Brain, Lung, Heart, Pancreas, Liver, Placenta
D139	***TTGCAG***	-	-
D140	***TAATA***	Lung, Heart, Lymphoma Burkitts Raji, Testis, Liver, Placenta, Whole Brain	PB-CD19+ B-cells, BM-CD33+ Myeloid, BM-CD34+, DRG, PB-CD56+ NK Cells, BM-CD105+

			Endothelial
D141	***AGTAT***	Lung, Heart, Lymphoma Burkitts Raji, Adipocyte	BM-CD34+
D142	***ATTCT***	Lung, Heart, Liver	Appendix
D143	***TGCCTTT***	Lung	-
D144	***ATCAAA***	Leukemia Lymphoblastic (molt4)	Atrioventricular Node
D145	***GAGTG***	-	Prefrontal Cortex, Prostate
D146	***TAGGT***	-	Fetal Liver
D147	***CTTTA***	Lung, Liver, Whole Brain	-
D148	***TTTAG***	Lung, Heart, Liver, Adrenal Gland, Prostate	Testis Interstitial, 721B Lymphoblasts, BM-CD105+ Endothelial, Trigeminal Ganglion, BM-CD34+, PB-CD19+ B-cells, Testis Germ Cell, Testis Leydig Cell, PB-CD4+ T-cells, PB-CD56+ NK Cells
D149	***TGATTTT***	Lung	Uterus
D150	***TTTCAT***	Lung, Heart, Lymphoma Burkitts Raji	Smooth Muscle, Fetal Brain, BM-CD34+, Superior Cervical Ganglion
D151	***CTTTCA***	-	-
D152	***AAGAT***	Heart, Lung	Appendix
D153	***TGCTT***	Lung, Heart	-
D154	***GTGGGT***	-	Whole Brain
D155	***GTAAAG***	-	Lymphoma Burkitts Daudi
D156	***CTGAA***	-	-
D157	***TCTGC***	-	Lung, Cerebellum Peduncles
D158	***CTAAA***	Heart	Testis Germ Cell, Testis Leydig Cell, Testis Interstitial, Superior Cervical Ganglion
U1	***GTTTGT***	-	Parietal Lobe
U2	***TCTCC***	BM-CD34+, 721B Lymphoblasts, PB-CD8 T-cells,	Cerebellum Peduncles, Lymphoma

		BM-CD105+ Endothelial	Burkitts Raji, Cerebellum, Caudate Nucleus, Whole Brain
U3	***GATTTT***	Heart, Cardiac Myocytes	PB-CD56+ NK Cells
U4	***TTTTTC***	Lymphoma Burkitts Raji, PB-CD14+ Monocytes, Colorectal Adenocarcinoma, Caudate Nucleus, Whole Brain, Liver, Tongue, Prostate, Kidney, Thalamus, Heart, Lung, Pancreas, Placenta, Cerebellum, Cardiac Myocytes	BM-CD33+ Myeloid, Testis Interstitial, PB-CD8 T-cells, Leukemia Promyelocytic (hl60), Testis Seminiferous Tubule, Trigeminal Ganglion, PB-CD19+ B-cells, Lymphoma Burkitts Daudi, PB-CD56+ NK Cells, Appendix, 721B Lymphoblasts, BM-CD105+ Endothelial, Testis Germ Cell, BM-CD34+, Testis Leydig Cell, Superior Cervical Ganglion
U5	***TAACC***	721B Lymphoblasts	-
U6	***TTGAAAT***	Heart, Lung, Tongue	Appendix, Superior Cervical Ganglion, Ciliary Ganglion
U7	***AAGCCA***	-	-
U8	***AATTG***	Lung, Heart	BM-CD105+ Endothelial
U9	***CTGCT***	-	Whole Brain
U10	***TTTATG***	Whole Brain, Thalamus	-
U11	***TTCACA***	-	-
U12	***TGATAA***	-	-
U13	***ATGTTT***	Heart, Lung	Appendix, Superior Cervical Ganglion
U14	***TCCAG***	BM-CD34+, PB-CD19+ B-cells, 721B Lymphoblasts, PB-CD56+ NK Cells, BM-CD105+ Endothelial	Pancreatic Islets, Lung, Adrenal Gland, Cerebellum, Placenta, Adipocyte, Cardiac

			Myocytes, Cingulate Cortex
U15	***TTTCCAA***	Lung, PB-CD14+ Monocytes, BM-CD33+ Myeloid	Superior Cervical Ganglion, Olfactory Bulb
U16	***TTATTTC***	Lung, Heart, Liver, Placenta	Testis Germ Cell, Fetal Brain, Testis Leydig Cell, Leukemia Promyelocytic (hl60), Ciliary Ganglion
U17	***TGTGTT***	Liver	-
U18	***TCTTG***	-	-
U19	***TTGTAA***	Heart	-
U20	***CTTGAC***	-	-
U21	***TTAAAAC***	-	-
U22	***CTAAC***	-	Prefrontal Cortex
U23	***AAAGCT***	-	-
U24	***TCTTC***	-	-
U25	***TGCATG***	Testis Germ Cell, Pancreas, Leukemia Chronic Myelogenous (k562)	Amygdala, Occipital Lobe, Fetal Brain, Prefrontal Cortex, Uterus
U26	***TTCTT***	Heart, Lung, Lymphoma Burkitts Raji, Liver, Bone Marrow, Whole Brain, Prostate	BM-CD33+ Myeloid, Testis Interstitial, BM-CD105+ Endothelial, Testis Seminiferous Tubule, Testis Germ Cell, BM-CD34+, PB-CD19+ B-cells, Testis Leydig Cell, Superior Cervical Ganglion
U27	***ACATTT***	Heart, Lung	Lymphoma Burkitts Daudi
U28	***TTTATC***	Colorectal Adenocarcinoma	-
U29	***ATTTTCT***	Lung, Testis, Liver, Colorectal Adenocarcinoma	BM-CD34+, Superior Cervical Ganglion
U30	***GCTGACC***	-	-
U31	***ATAATT***	Lung, Heart, Liver, Colorectal Adenocarcinoma	Occipital Lobe, Atrioventricular Node, DRG, Appendix, Superior Cervical

			Ganglion, Ciliary Ganglion
U32	***AACAG***	Heart, Liver	PB-CD19+ B-cells
U33	***CATTTG***	Heart	-
U34	***TTTCAG***	Lung, Heart, Lymphoma Burkitts Raji, Liver, Colorectal Adenocarcinoma	Testis Leydig Cell, Hypothalamus, Testis Interstitial, Superior Cervical Ganglion, Lymphoma Burkitts Daudi, Testis Seminiferous Tubule, Trigeminal Ganglion
U35	***TTAGCA***	-	-
U36	***TTGCCT***	-	-
U37	***CAAAT***	Lung, Liver, Colorectal Adenocarcinoma	Fetal Brain, Skin, Occipital Lobe, Testis Leydig Cell, Testis Interstitial, Fetal Liver, Ciliary Ganglion
U38	***TTTAAC***	Heart, Lung, Liver	-
U39	***TAATG***	Lung, Testis	Appendix, Superior Cervical Ganglion
U40	***AGAAAT***	Heart, Lymphoma Burkitts Raji	Tonsil, Appendix
U41	***TTTTGAT***	PB-CD14+ Monocytes	-
U42	***TAACT***	Heart, Lung, Lymphoma Burkitts Raji, Liver	BM-CD34+, Fetal Brain, Prefrontal Cortex, Testis Leydig Cell
U43	***TAAAAT***	Heart, Lung, Lymphoma Burkitts Raji, Liver, Colorectal Adenocarcinoma, Whole Brain	BM-CD33+ Myeloid, Appendix, 721B Lymphoblasts, BM-CD105+ Endothelial, PB-CD19+ B-cells, BM-CD34+, Superior Cervical Ganglion, PB-CD56+ NK Cells, Ciliary Ganglion
U44	***AATTACA***	BM-CD33+ Myeloid	-

U45	***TTCAAAA***	-	Atrioventricular Node
U46	***TTTATA***	Lung, Heart, Liver	BM-CD34+, Testis Interstitial, BM-CD105+ Endothelial, Ciliary Ganglion
U47	***CTTGTC***	-	Leukemia Promyelocytic (hl60)
U48	***TTTACAG***	Liver	-
U49	***TGGATTT***	-	PB-CD56+ NK Cells, PB-CD8 T-cells
U50	***TTGCATT***	-	-
U51	***TTAAG***	Cerebellum Peduncles, Heart, Lung, Liver, Cerebellum, Whole Brain	PB-CD19+ B-cells, BM-CD34+, Testis Leydig Cell, Superior Cervical Ganglion, PB-CD56+ NK Cells, 721B Lymphoblasts, BM-CD105+ Endothelial
U52	***TTGGT***	-	-
U53	***CATAAA***	-	-
U54	***TGATTA***	-	-
U55	***GCTTTGC***	Appendix	-
U56	***ATTAG***	Heart, Lung, Liver	Testis Leydig Cell, Superior Cervical Ganglion, Hypothalamus
U57	***TTTTAAA***	Lung, Heart, Liver, Cerebellum, Whole Brain	BM-CD34+, Testis Interstitial, BM-CD105+ Endothelial
U58	***ACTAAT***	Heart, Liver	Occipital Lobe, Trigeminal Ganglion
U59	***CTGACT***	BM-CD34+	-
U60	***ATATTT***	Heart, Lung, Thymus	Testis Germ Cell, Testis Interstitial
U61	***CATTTA***	Heart	-
U62	***AAATCT***	Heart, Liver	Ovary, Appendix, Superior Cervical Ganglion
U63	***TTTTGGC***	-	-
U64	***TCTTT***	Heart, Lung, Thyroid, Liver, Adrenal Gland, Colorectal Adenocarcinoma, Placenta	PB-CD19+ B-cells, BM-CD34+, Appendix, Superior Cervical Ganglion, PB-

U65	***TTATTGA***	-	CD56+ NK Cells Prefrontal Cortex
U66	***TCAGA***	721B Lymphoblasts, PB-CD56+ NK Cells	-
U67	***TAAGT***	Heart, Liver	BM-CD34+, PB-CD4+ T-cells, 721B Lymphoblasts, PB-CD8 T-cells, BM-CD105+ Endothelial
U68	***CTCTG***	BM-CD34+, PB-CD19+ B-cells, 721B Lymphoblasts, Lymphoma Burkitts Daudi, BM-CD105+ Endothelial, PB-BDCA4+ Dentritic Cells, Leukemia Promyelocytic (hl60)	Cerebellum Peduncles, Lung, Heart, Liver
U69	***TCTGG***	BM-CD34+	Lung, Kidney, Cerebellum
U70	***AATTC***	-	Appendix, Trigeminal Ganglion
U71	***TTTTCC***	-	-
U72	***TTTTGC***	Heart	-
U73	***GTGAG***	Atrioventricular Node, Testis Interstitial, Testis Seminiferous Tubule, Trigeminal Ganglion, Medulla Oblongata, Occipital Lobe, Appendix, Hypothalamus, Fetal Liver, Skin, Ovary, BM-CD34+, Testis Germ Cell, Testis Leydig Cell, Superior Cervical Ganglion, Adipocyte, Skeletal Muscle, Ciliary Ganglion	PB-CD14+ Monocytes, Lymphoma Burkitts Raji, Colorectal Adenocarcinoma, PB-BDCA4+ Dentritic Cells, Whole Brain, Lung, Heart, Liver, Bone Marrow
U74	***TCCATTT***	Liver	-

U75	***AATTTT***	Lung, Heart, Liver, Adrenal Gland, Colorectal Adenocarcinoma	BM-CD34+, 721B Lymphoblasts
U76	***CTTGATT***	-	-
U77	***GTAAG***	Occipital Lobe, Prefrontal Cortex, Atrioventricular Node, Ovary, Parietal Lobe, Superior Cervical Ganglion, Ciliary Ganglion	PB-CD8 T-cells, 721B Lymphoblasts, BM-CD105+ Endothelial, PB-BDCA4+ Dentritic Cells, BM-CD34+, PB-CD4+ T-cells, Lymphoma Burkitts Daudi, PB-CD56+ NK Cells
U78	***ATGAAA***	Lung, Heart, Liver	Ovary, Testis Germ Cell, Testis Interstitial, Superior Cervical Ganglion
U79	***AGAAAA***	Heart, Lung, Liver	Superior Cervical Ganglion
U80	***TGGCTT***	-	Cerebellum
U81	***CTCAG***	BM-CD34+, PB-CD19+ B-cells, 721B Lymphoblasts, BM-CD105+ Endothelial	Lung, Heart, Cerebellum, Whole Brain
U82	***TGGAAAT***	-	-
U83	***AATTAT***	Lung	-
U84	***AATAAT***	Lymphoma Burkitts Raji	Atrioventricular Node, Skin, Appendix, Testis Interstitial
U85	***TCCTAG***	-	Fetal Liver, Uterus Corpus
U86	***CCACAG***	BM-CD34+, Testis Germ Cell, 721B Lymphoblasts	Cingulate Cortex
U87	***TCATTTC***	-	Prefrontal Cortex, Occipital Lobe, Parietal Lobe, Cingulate Cortex
U88	***AAAGCA***	BM-CD34+, 721B Lymphoblasts	-
U89	***AAATGA***	Heart, Lung	Testis Leydig Cell
U90	***TTTATAG***	Lung, Heart, Liver	Trigeminal Ganglion
U91	***ATTAAAT***	-	Appendix, DRG

U92	***CCTGCAG***	-	Lung, Whole Brain
U93	***TTACAG***	Heart, Thalamus	-
U94	***AAATGT***	Heart, Lung, Testis, Liver, Tongue	Appendix, Superior Cervical Ganglion
U95	***TGCAT***	Lymphoma Burkitts Raji	-
U96	***CTTCT***	BM-CD34+	-
U97	***TTAGAA***	Heart, Lung, PB-CD14+ Monocytes, Liver, Whole Brain, Prostate	BM-CD34+, Appendix, Superior Cervical Ganglion, Trigeminal Ganglion
U98	***TGTTTC***	Heart	-
U99	***TTTAC***	Heart, Lung, Liver, Placenta	Testis Germ Cell
U100	***GTTTT***	Heart, Lung, Pancreas, Liver, Trachea, Colorectal Adenocarcinoma, Tongue, Whole Brain	PB-CD19+ B-cells, BM-CD34+, Testis Leydig Cell, PB-CD56+ NK Cells, 721B Lymphoblasts, BM-CD105+ Endothelial
U101	***CTTCCA***	BM-CD34+, 721B Lymphoblasts	Heart, Trigeminal Ganglion
U102	***TTCTAG***	Lung, Heart, Lymphoma Burkitts Raji, PB-CD14+ Monocytes, Liver, Bone Marrow	Ovary, Testis Leydig Cell, Superior Cervical Ganglion, Testis Interstitial, Testis Seminiferous Tubule
U103	***AAATT***	PB-CD14+ Monocytes, Colorectal Adenocarcinoma, Whole Brain, Lung, Heart, Liver, Tongue, Placenta, Prostate	Fetal Brain, Appendix, Testis Interstitial, 721B Lymphoblasts, BM-CD105+ Endothelial, Trigeminal Ganglion, Testis Seminiferous Tubule, PB-CD19+ B-cells, Ovary, BM-CD34+, Testis Germ Cell, Testis Leydig Cell, Superior Cervical Ganglion, PB-

			CD56+ NK Cells, Ciliary Ganglion
U104	***TTAAAC***	Lung, Whole Brain	-
U105	***TGAGAA***	-	-
U106	***TTTGTAG***	Lung, Liver, Spinal Cord	BM-CD34+, 721B Lymphoblasts, BM-CD105+ Endothelial
U107	***GTCAGT***	Temporal Lobe	-
U108	***TAAGA***	-	-
U109	***AAATCA***	Lung, Testis	Fetal Liver
U110	***TGTTGA***	-	-
U111	***CTTGC***	-	-
U112	***TAATTTG***	Heart	Testis Interstitial, BM-CD105+ Endothelial
U113	***CCTCT***	BM-CD34+, 721B Lymphoblasts, BM-CD105+ Endothelial	Heart, Prefrontal Cortex, Occipital Lobe, Cerebellum, Whole Brain
U114	***TGGTTT***	Liver	BM-CD34+
U115	***TGATTTC***	-	-
U116	***TGTTAA***	Heart, Lung, Lymphoma Burkitts Raji, Liver, Placenta, Colorectal Adenocarcinoma, Prostate	BM-CD34+, 721B Lymphoblasts, BM-CD105+ Endothelial, Leukemia Promyelocytic (hl60), Ciliary Ganglion
U117	***TGTGTC***	-	Uterus Corpus
U118	***TCTCT***	721B Lymphoblasts, PB-BDCA4+ Dentritic Cells, BM-CD105+ Endothelial, Bronchial Epithelial Cells, Leukemia Promyelocytic (hl60), BM-CD34+, PB-CD19+ B-cells, Lymphoma Burkitts Daudi	Fetal Brain, Occipital Lobe, Caudate Nucleus, Cingulate Cortex, Whole Brain, Medulla Oblongata, Heart, Cerebellum Peduncles, Prefrontal Cortex, Cerebellum
U119	***TTAACA***	Lung, Heart, Liver	Fetal Brain, BM-CD34+, Superior Cervical Ganglion, 721B Lymphoblasts
U120	***TTTGGT***	-	BM-CD34+
U121	***TGTCT***	-	Cerebellum Peduncles,

			Prefrontal Cortex, Parietal Lobe, Cingulate Cortex
U122	***TTCCTT***	Colorectal Adenocarcinoma	-
U123	***TGAATT***	Lung	Superior Cervical Ganglion
U124	***AATTTA***	Lung, Heart, Placenta	Atrioventricular Node, Testis Leydig Cell, Testis Interstitial, Ciliary Ganglion
U125	***GTTTCT***	Colorectal Adenocarcinoma, Globus Pallidus	-
U126	***TGCTAA***	Testis	Caudate Nucleus
U127	***AGATTT***	-	-
U128	***GAAAAT***	Heart, Lung, Lymphoma Burkitts Raji, Liver	Testis Leydig Cell, Superior Cervical Ganglion, Testis Interstitial, Testis Seminiferous Tubule
U129	***GTTTAAT***	Heart, Lung, Adrenal Gland, Colorectal Adenocarcinoma	-
U130	***TTTGACT***	-	-
U131	***TCTGA***	-	-
U132	***TCTGTT***	BM-CD34+, BM-CD105+ Endothelial	Prefrontal Cortex, Placenta, Kidney, Olfactory Bulb
U133	***TTTATTC***	-	-
U134	***TGAAAG***	-	-
U135	***TGTTCT***	-	PB-CD19+ B-cells
U136	***CTTTT***	Lymphoma Burkitts Raji, Colorectal Adenocarcinoma, Heart, Lung, Liver, Bone Marrow, Placenta, Tongue, Prostate	PB-CD19+ B-cells, Fetal Brain, BM-CD34+, 721B Lymphoblasts, PB-CD8 T-cells, PB-CD56+ NK Cells
U137	***ATTTGT***	Heart, Lung	Testis Interstitial, Ciliary Ganglion
U138	***TGAGT***	Testis Leydig Cell, Testis Interstitial, Ciliary Ganglion	Lung, Heart, Lymphoma Burkitts Raji

U139	***CCCCAG***	Testis Interstitial, 721B Lymphoblasts, BM-CD105+ Endothelial, PB-BDCA4+ Dentritic Cells, Leukemia Promyelocytic (hl60), Testis Seminiferous Tubule, Pituitary, Leukemia Lymphoblastic (molt4), BM-CD34+, Testis Germ Cell, Testis Leydig Cell, PB-CD56+ NK Cells	Lymphoma Burkitts Raji, Kidney, Colorectal Adenocarcinoma, Thalamus, Cingulate Cortex, Whole Brain, Cerebellum Peduncles, Heart, Lung, Tonsil, Liver, Cerebellum, Cardiac Myocytes
U140	***TTGCAG***	-	-
U141	***CTGAT***	-	Trigeminal Ganglion
U142	***TAATA***	Colorectal Adenocarcinoma, Whole Brain, Cerebellum Peduncles, Lung, Heart, Testis, Liver, Cerebellum, Placenta	PB-CD19+ B-cells, BM-CD34+, PB-CD56+ NK Cells, 721B Lymphoblasts, Trigeminal Ganglion
U143	***TCTTA***	Lung, Heart, Liver	Atrioventricular Node, BM-CD33+ Myeloid, BM-CD34+, Superior Cervical Ganglion, 721B Lymphoblasts, PB-BDCA4+ Dentritic Cells, Leukemia Promyelocytic (hl60)
U144	***ATTCT***	Lung, Heart, Lymphoma Burkitts Raji, Liver	Occipital Lobe, Hypothalamus
U145	***ATCAAA***	-	Fetal Brain
U146	***CTTTA***	Heart, Lung, Liver, Cerebellum	BM-CD34+
U147	***TTTAG***	Colorectal Adenocarcinoma, Whole Brain, Lung, Cerebellum Peduncles,	BM-CD33+ Myeloid, Testis Interstitial, 721B Lymphoblasts,

		Heart, Pancreas, Adrenal Gland, Liver, Tonsil, Placenta, Cerebellum	BM-CD105+ Endothelial, Leukemia Promyelocytic (hl60), Testis Seminiferous Tubule, Testis Germ Cell, BM-CD34+, PB-CD19+ B-cells, Testis Leydig Cell, Superior Cervical Ganglion, PB-CD56+ NK Cells, Lymphoma Burkitts Daudi
U148	***TTGCTG***	Cerebellum Peduncles	-
U149	***TTTCAT***	Lung, Heart, Testis, Liver, Cerebellum	BM-CD34+, Occipital Lobe, Superior Cervical Ganglion, 721B Lymphoblasts
U150	***CTTTCA***	Lung, Liver	Occipital Lobe, Superior Cervical Ganglion
U151	***TTCTC***	PB-CD14+ Monocytes	-
U152	***TGCTT***	Lung, Heart, Liver, Colorectal Adenocarcinoma	-
U153	***TCTGC***	Fetal Liver	-
U154	***CTGAA***	Lung, Liver	PB-CD19+ B-cells, Lymphoma Burkitts Daudi
U155	***GTAGGT***	Pancreas, Testis Seminiferous Tubule	-
U156	***CTAAA***	Lung, Heart, Lymphoma Burkitts Raji	Occipital Lobe, Fetal Brain, Testis Leydig Cell
