# Supplementary material for: Discovery and Analysis of Evolutionarily Conserved Intronic Splicing Regulatory Elements
Source: PLoS Genet. 2007 May 25;3(5):e85. doi: 10.1371/journal.pgen.0030085 (PMC1877881; doi:10.1371/journal.pgen.0030085)
Supplement: Table S6 — (104 KB DOC) [file pgen.0030085.st006.doc]

Supplementary Table 6. Ultraconserved elements (UCEs) which overlapped ACEScan positive

exons (hg17 coordinates)

| Chr | UCE start | UCE end | ACE Exon start | ACE Exon end | Strand | Description of gene containing ACE |
| --- | --- | --- | --- | --- | --- | --- |
| 1 | 96983758 | 96984074 | 96983995 | 96984029 | 1 | polypyrimidine tract binding protein 2 |
| 1 | 36028283 | 36028520 | 36028367 | 36028504 | 1 | eukaryotic translation initiation factor 2C, 1 |
| 1 | 23412598 | 23412866 | 23412650 | 23412816 | -1 | heterogeneous nuclear ribonucleoprotein R |
| 1 | 114992095 | 114992296 | 114992133 | 114992226 | -1 | upstream of NRAS isoform 2 |
| 2 | 144543340 | 144543629 | 144543280 | 144543372 | -1 | glycosyltransferase-like domain containing 1 |
| 2 | 20399961 | 20400258 | 20399971 | 20400208 | -1 | pumilio homolog 2 |
| 2 | 50193686 | 50193904 | 50193743 | 50193833 | -1 | neurexin 1 isoform alpha precursor |
| 2 | 63135459 | 63135742 | 63135428 | 63135552 | 1 | EH domain binding protein 1 |
| 2 | 174771216 | 174771422 | 174771386 | 174771483 | -1 | GTP-binding protein PTD004 isoform 1 |
| 2 | 174771916 | 174772253 | 174772158 | 174772299 | -1 | GTP-binding protein PTD004 isoform 1 |
| 2 | 172648138 | 172648584 | 172648436 | 172648541 | 1 | histone acetyltransferase 1 |
| 3 | 153647085 | 153647296 | 153647190 | 153647244 | 1 | muscleblind-like 1 isoform a |
| 4 | 76936303 | 76936520 | 76936345 | 76936444 | -1 | Ras-GTPase activating protein SH3 domain-binding |
| 5 | 178978803 | 178979111 | 178978875 | 178979014 | -1 | heterogeneous nuclear ribonucleoprotein H1 |
| 5 | 72231442 | 72231690 | 72231588 | 72231664 | 1 | transportin 1 |
| 5 | 32415894 | 32416107 | 32415936 | 32416035 | -1 | zinc finger RNA binding protein |
| 5 | 94232224 | 94232438 | 94232332 | 94232434 | -1 | multiple C2-domains with two transmembrane |
| 6 | 94025785 | 94025985 | 94025792 | 94025918 | -1 | ephrin receptor EphA7 |
| 6 | 86381217 | 86381435 | 86381417 | 86381784 | -1 | synaptotagmin binding, cytoplasmic RNA |
| 9 | 125757560 | 125757780 | 125757518 | 125757760 | 1 | pre-B-cell leukemia transcription factor 3 |
| 9 | 137318327 | 137318534 | 137318440 | 137318503 | 1 | NMDA receptor 1 isoform NR1-2 precursor |
| 9 | 83819838 | 83820044 | 83819930 | 83819974 | -1 | heterogeneous nuclear ribonucleoprotein K |
| 9 | 122133444 | 122133646 | 122133581 | 122133673 | 1 | mitochondrial ribosome recycling factor isoform |
| 10 | 98705445 | 98705661 | 98704699 | 98705581 | 1 | ligand-dependent corepressor |
| 10 | 11348467 | 11348706 | 11348566 | 11348646 | 1 | CUG triplet repeat, RNA binding protein 2 |
| 11 | 31741481 | 31741710 | 31741534 | 31741673 | 1 | elongation protein 4 homolog |
| 11 | 82872807 | 82873024 | 82872817 | 82872919 | -1 | chapsyn-110 |
| 12 | 52144738 | 52144978 | 52144810 | 52144903 | 1 | poly(rC)-binding protein 2 isoform a |
| 12 | 52733867 | 52734167 | 52733967 | 52734412 | 1 | homeo box C4 |
| 12 | 52708708 | 52709096 | 52708626 | 52708972 | 1 | homeo box C6 isoform 2 |
| 13 | 96806821 | 96807125 | 96807050 | 96807104 | 1 | - |
| 14 | 36846966 | 36847270 | 36847011 | 36847045 | 1 | mirror-image polydactyly 1 |
| 14 | 44635500 | 44635789 | 44635376 | 44635711 | 1 | PRP39 pre-mRNA processing factor 39 homolog |
| 15 | 65665156 | 65665428 | 65665281 | 65665322 | 1 | mitogen-activated protein kinase kinase 5 |
| 15 | 65827358 | 65827675 | 65827622 | 65827649 | 1 | mitogen-activated protein kinase kinase 5 |
| 15 | 72701295 | 72701569 | 72701513 | 72701610 | 1 | CDC-like kinase 3 isoform hclk3 |
| 16 | 68237865 | 68238075 | 68237920 | 68237982 | 1 | nuclear factor of activated T-cells 5 isoform a |
| 16 | 24486504 | 24486752 | 24486613 | 24486715 | 1 | retinoblastoma-binding protein 6 isoform 1 |
| 17 | 22658849 | 22659082 | 22658896 | 22660425 | 1 | WD SOCS-box protein 1 isoform 3 |
| 17 | 34820045 | 34820421 | 34820359 | 34820500 | -1 | peroxisome proliferator-activated receptor |
| 18 | 51405239 | 51405448 | 51405273 | 51405365 | -1 | transcription factor 4 isoform b |
| 19 | 8433269 | 8433507 | 8433412 | 8433465 | 1 | heterogeneous nuclear ribonucleoprotein M |
| 20 | 10221745 | 10221956 | 10221808 | 10221926 | 1 | synaptosomal-associated protein 25 isoform |
| 20 | 33791793 | 33792037 | 33791860 | 33791933 | -1 | RNA-binding region containing protein 2 isoform |
| 22 | 17770463 | 17770673 | 17770559 | 17770670 | -1 | HIR (histone cell cycle regulation defective, S. |
| X | 102847636 | 102847844 | 102847538 | 102847800 | 1 | proteolipid protein 1 isoform 1 |
| X | 122324793 | 122325243 | 122325059 | 122325174 | 1 | glutamate receptor 3 isoform flop precursor |
| X | 70156245 | 70156466 | 70156347 | 70156407 | 1 | neuroligin 3 |
| X | 21294217 | 21294474 | 21294259 | 21294406 | 1 | connector enhancer of kinase suppressor of Ras |
