# Supplementary material for: Discovery and Analysis of Evolutionarily Conserved Intronic Splicing Regulatory Elements
Source: PLoS Genet. 2007 May 25;3(5):e85. doi: 10.1371/journal.pgen.0030085 (PMC1877881; doi:10.1371/journal.pgen.0030085)
Supplement: Protocol S1 — (64 KB DOC) [file pgen.0030085.sd001.doc]

Protocol S1

**Supplementary Information for Evolutionarily Conserved Mammalian Intronic Splicing Regulatory Elements**

Exon-intron datasets

Genome sequences and alignments: the genome sequences of human (hg17), dog (canFam1), rat (rn3) and mouse (mm5) were obtained from the University of California Santa Cruz (UCSC), as were the whole-genome multiz and pair-wise alignments. The four-way mammalian (4-mammal) whole-genome alignment (hg17, canFam1, mm5, rn3) was extracted from the 8-way vertebrate multiz alignments (hg17, panTrol1, mm5, rn3, canFam1, galGal2, fr1, danRer1). The lists of known human genes (obtained March 2005; knownGene.txt.gz; containing 43,401 entries) and known isoforms (knownIsoforms.txt.gz; containing 43,286 entries in 21,397 unique isoform clusters) with annotated exon alignments to human hg17 genomic sequence were processed as follows. Known genes that were mapped to different isoform clusters were discarded. All mRNAs aligned to hg17 that were greater than 300 bases long were clustered with the known isoforms. Genes containing less than 3 exons were also removed from further consideration.

Human promoter sequences

Approximately 20,000 human, mouse, rat and dog (hg17, mm5, rn3 and canFam1) alignments representing the 2kb upstream and downstream of the transcriptional start site of human promoters were obtained from the supplementary materials of Xie et al. 2005. Conservation enrichment scores were computed as described below. K-mers (5,6 and 7) with corrected p-values less than 0.001 were excluded from enriched k-mers calculated from the intronic regions.

Xie X, Lu J, Kulbokas EJ, Golub TR, Mootha V, Lindblad-Toh K, Lander ES, Kellis M. (2005) Systematic discovery of regulatory motifs in human promoters and 3' UTRs by comparison of several mammals. Nature. 2005 Mar 17;434(7031):338-45.

Calculating conservation scores for k-mers to discover ISREs

The conservation score (S) of a sequence element of length *k* (k-mer) was represented by the non-parametric χ^2^ statistic with Yates correction, computed from the two by two contingency table, T (T_11_: number of occurrences of the element perfectly conserved across alignments; T_12_: number of occurrences of all other conserved elements of similar length; T_21_: number of occurrences of element in the reference genome only (human); T_22_: number of occurrences of all other elements of similar length in the reference genomes). Counts in the table had to be greater than 10. To correct for multiple hypothesis testing, p-values were multiplied by the total number of comparisons. Enriched elements with corrected p-values less than 0.001 were considered significant.

Clustering k-mers to generate ISRE

*Generating families (parents and children).* To cluster elements, we first perform a ‘backward’ pass, going from the longest elements to the shortest elements. If a (k+1)-bp element contains a perfect match to a j-bp element where j is less than (k+1)-bp long, the enrichment scores of both elements are compared. The lower-scoring element (child element) is designated to ‘belong’ to the higher-scoring element (parent element). Member elements are henceforth represented by the core element. Elements with no cores are designated cores. Next we perform a ‘forward’ pass, where k-bp core elements are compared to j-bp core elements, where j is greater than k-bp long. Similarly, the lower-scoring element is designed as a child of the higher-scoring element, designated parent. At this stage, it is possible that some children have multiple parents i.e. different parents may share children.

*Collapsing families.* Two families are collapsed if the number of shared children is larger than the size of the smaller family. The new parent is the higher-scoring parent.

*Matching parents.* Next, if parents are exactly overlapping by 5 bases or more, families are combined again. At this stage, singletons are removed (parents with no families).

*Identifying unique parents.*  Finally, children that are shared by different parents are matched to the closer parent, based on hamming distance between multiple parents.

Our clustering strategy is conservative in that k-mers that might have been clustered together by other approaches are purposely kept separate. For example, motifs D20 and D50 are not clustered together, and are identified as separate clusters (below). One argument for our approach using this example is that there exists a conserved k-mer AGCATG that did not fit in motif D20, but can be explained by motif D50. We prefer, subjectively of course, to leave these ISREs separate and later, by experimental means, to group D20 and D50 as being both substrates of the same splicing factor, if that is true. In this case, the mammalian ortholog of FOX1 binds to both ISREs.

D50 GCATG, AGCATG, GCATGT, GCATGAA, GCATGGC

D20 TGCATG,TGCATGA, ATGCATG, CTGCATG, TGCATGC, TGCATGT, TGCATGG, GTGCATG

Representation of ISRE and enumeration of ISRE occurrences

An ISRE can be represented as (i) a set of sequences, or (ii) a weight matrix. As an example, both representations for ISRE D20 are depicted below.

Set representation for D20, with the parent TGCATG represented in **bold**. The parent is not necessarily the shortest sequence of the set, but occurs with the highest conservation score.

**TGCATG**, TGCATGA, ATGCATG, CTGCATG, TGCATGC, TGCATGT, TGCATGG, GTGCATG

To generate the weight matrix for each ISRE, we identified all occurrences of conserved ISREs (all sequences in the set representation) in their respective intronic regions, and extracted the ISRE and a single nucleotide flanking the ISRE from the human sequence. The weight matrix representation for D20 is depicted below.

A C G T

1 0.275 0.199 0.204 0.323

2 0.212 0.278 0.213 0.297

3 0.000 0.000 0.000 1.000

4 0.000 0.000 1.000 0.000

5 0.000 1.000 0.000 0.000

6 1.000 0.000 0.000 0.000

7 0.000 0.000 0.000 1.000

8 0.000 0.000 1.000 0.000

9 0.203 0.258 0.215 0.324

10 0.241 0.232 0.229 0.299

To avoid over-counting, if a sequence in the ISRE set is present, for example ATGCATG (bold and underlined), if another sequence in the set (TGCATG) overlaps the sequence (within 5 bases), then the ISRE is only counted once.

CGATGCTACGATAAATGC**ATGCATG**ACGCGCTA

Calculation of Positional Bias

In order to determine if a conserved splicing regulatory element (SRE) was enriched at particular positions in the intronic regions, we constructed a Z-score representing the enrichment of the conserved SRE at a particular position above background in multiple-aligned sequences. First we define an alignment block as a segment of sequence in human that is aligned across multiple organisms. Given a sequence M (e.g. TGCATG, below) of L-bases (e.g. L=6) long and a window of size W beginning at position j in the alignment block, we enumerate the number of times that M is perfectly conserved across the alignment (see example below). Second, we randomly move columns of bases that are perfectly conserved with other columns that are also perfectly conserved. In our example below, column c is swapped with column q, and column j is swapped with column u. This method of shuffling preserves the local sequence biases in a window, the conservation rate of every nucleotide across multiple genomes, and the length distribution of conserved sequences. Thirdly, each window was shuffled twenty times, storing the number of times M is perfectly conserved in each shuffled alignment. This is repeated for all alignment blocks (e.g. all downstream intronic regions). The Z-score Z(M,j) for a sequence M at a window of size W beginning at position j is calculated as (M(j) – U(j))/S(j), where M(j) is the number of occurrences of conserved M at windows beginning at position j, U(j) is the average number of occurrences of conserved M at shuffled windows beginning at position j; and S(j) is the standard deviation of the occurrences of conserved M at shuffled windows beginning at position j. An SRE M of Z(M,j) greater or equal to 4.5 is considered significantly enriched in windows beginning at position j (P-value of 1E-5).

Example:

Unshuffled alignment block: TGCATG occurs once in this window. Row 1-4 represents the orthologous sequence from different species, and row 5 represents the alignment. N represents imperfect conserved nucleotides in a column.

1 ATCGGCTGCATGACGTGCTAT

2 CACGGTTGCATGCACTGCCTT

3 GTCAGATGCATGGAGTGCAGT

4 TTCGGCTGCATGACCTGCGCT

**5 NNCNGNTGCATGNNNTGCNNT**

abcdefghijklmnopqrstu

Shuffled alignment block, preserving regions of conservation, TGCATG does not occur in this shuffled block.

1 ATGGGCTGCTTGACGTCCTAA

2 CAGGGTTGCTTGCACTCCCTA

3 GTGAGATGCTTGGAGTCCAGA

4 TTGGGCTGCTTGACCTCCGCA

**5 NNGNGNTGCTTGNNNTCCNNA**

abcdefghijklmnopqrstu

Identifying canonical signals by positional biases

To determine which ISREs are 5′ splice sites and 3′ splice sites, we generated Z-scores for positional enrichment, starting at j (j ranging from position +1 to +10, and -20 to -10 in introns) at 1 base increments, with windows of 10 bases long. Significantly higher (10-1000-fold) Z-scores at positions +1, +2 and +3 are consistent with 5′ splice sites. Significantly higher Z-scores at positions -2 and -1 are consistent with the 3′ splice sites.

# Discussion of conserved U12-type introns in mammals

# Four hundred and seventeen exons from three hundred and seventy-nine genes were found to contain perfectly conserved U12-type 5′ splice sites (ATATCCT and GTATCCT from positions +1 to +7 of the intron). Approximately 25% (102 introns) contained the U12 branch signal “CCTT[AG]AC”, were located in a U12-type intron, 8-15 bases from the 3′ splice site. This demonstrates that approximately 2% of mammalian genes contain U12-type introns, which may be an underestimate, as genome-wide alignments are likely to miss some introns, and gene annotations are known to be incomplete. In addition, 8.4% (32 genes) of U12-type intron-containing genes have more than one U12-type intron in the same genes, three-fold higher than expected by chance. In fact, the sodium/hydrogen exchanger 6 and 7 genes (NHE-6 and NHE-7) contained three U12-type introns. Genes with two U12-type introns are as follows: solute carrier family 24 member 6, hypothetical protein FLJ31795, chromodomain helicase DNA binding protein 4, solute carrier family 12 member 5, cofactor required for Sp1 transcriptional, mitogen-activated protein kinase 10 isoform 2, hypothetical protein FLJ25037, diaphanous homolog 3, mitogen-activated protein kinase 9 isoform 1, zinc finger, CW-type with coiled-coil domain 3, potassium chloride cotransporter KCC3, solute carrier family 12 (potassium/chloride, kinetochore protein Spc24, Vac14 homolog, suppressor of actin mutations 2-like isoform a, xanthine dehydrogenase, calcium channel, voltage-dependent, alpha, kinesin-associated protein 3, ring finger protein 121 isoform 1, mitogen-activated protein kinase 8 isoform 2, spermine synthase, crystallin, zeta-like 1 isoform a, hypothetical protein FLJ39075, KIAA1036, diaphanous 1, HECT domain containing 2 isoform a, adaptor-related protein complex 2, alpha 2, exportin 7, guanylate binding protein 2,, RAN binding protein 17 and polymerase (RNA) III (DNA directed) polypeptide.

# An analysis of the Gene Ontology Consortium terms found a statistically significant enrichment (p<0.001) in genes involved with signaling and “information-processing” (MAP kinase activity, ATP binding, phosphoinositide phospholipase C activity, guanyl-nucleotide exchange factor activity). The biological processes that are significantly enriched are chloride transpor, cation transport, protein-nucleus import, docking, mRNA-nucleus export, response to stress, sodium ion transport and small GTPase mediated signal transduction. This confirms cursory observations on smaller sets of genes containing U12-type introns (Burge, Padgett et al. 1998). Analysis of gene expression from available microarray data (Su, Wiltshire et al. 2004) showed interestingly that U12-type intron-containing genes were biased for differential expression in bone marrow CD34 positive cells and B lymphoblasts.

Burge CB, Padgett RA, Sharp PA. Evolutionary fates and origins of U12-type introns. (1998) Mol Cell. Dec;2(6):773-85.

Su AI, Wiltshire T, Batalov S, Lapp H, Ching KA, Block D, Zhang J, Soden R, Hayakawa M, Kreiman G, Cooke MP, Walker JR, Hogenesch JB. (2004) A gene atlas of the mouse and human protein-encoding transcriptomes. Proc Natl Acad Sci U S A. Apr 20;101(16):6062-7.

Overlap of ISREs with known splicing regulatory elements

We obtained three sets of splicing regulatory elements in order to determine if our candidate intronic splicing regulatory elements (ISREs) have other splicing regulatory roles. Specifically, we tested if our ISREs overlapped with existing exonic splicing enhancers (ESEs), exonic splicing silencers (ESS) and intronic splicing enhancers (ISEs), that were identified from previous systematic computational or experimental screens. Two sequences were considered to overlap if the shorter sequence is a substring of the longer sequence. Mammalian-specific ISEs were obtained from (Yeo et al, 2004); ISEs were divided into ISEs that were enriched in the intronic regions near the 5′ss (ISE5) and ISEs that are enriched near the 3′ss (ISE33). We utilized a set of ‘trusted’ exonic splicing enhancer (ESE) and exonic splicing silencer (ESS) hexanucleotides that were previously generated (*in press* Stadler et al 2006). In summary, the authors pooled two sources of ESE to generate a trusted set of 666 ESEs: 238 hexamers from the RESCUE-ESE method (Fairbrother et al 2002), and 537 hexamers (each hexamer had to be observed at least 4 times) extracted from 2,069 PESE octamers (Zhang and Chasin, 2004). The authors also pooled two sources of ESS to generate a trusted set of 386 ESS: 176 hexamers (each hexamer had to be observed twice) extracted from 133 decamers (Wang et al. 2004), and 240 hexamers (each hexamer had to be observed at least 4 times) extracted from 974 PESS octamers (Zhang and Chasin, 2004). The splicing regulatory elements that were generated by large-scale comparisons of exonic and intronic sequences (Fairbrother et al, 2002; Zhang and Chasin, 2004; Yeo et al, 2004) are likely to be involved with constitutive splicing, because ~10% of exons in the genome are alternatively spliced.

Fairbrother WG, Yeh RF, Sharp PA, Burge CB (2002) Predictive identification of exonic splicing enhancers in human genes. Science 297(5583): 1007-1013.

Wang Z, Rolish ME, Yeo G, Tung V, Mawson M et al. (2004) Systematic identification and analysis of exonic splicing silencers. Cell 119(6): 831-845.

Stadler M.B., Shomron, N., Yeo, G.W, Schneider, A. Xiao, X. and Burge, C.B (2006). Inference of Splicing Regulatory Activities by Sequence Neighborhood Analysis', *Plos Genetics,* in press.

Yeo G, Hoon S, Venkatesh B, Burge CB (2004) Variation in sequence and organization of splicing regulatory elements in vertebrate genes. Proc Natl Acad Sci U S A 101(44): 15700-15705.

Zhang XH, Chasin LA (2004) Computational definition of sequence motifs governing constitutive exon splicing. Genes Dev 18(11): 1241-1250.

Calculation of Z-score for enrichment near alternatively spliced exons

Human exons with transcript evidence (human mRNAs and expressed sequence tages) for exon inclusion/exclusion were designated skipped exons. Constitutive exons are exons with no evidence for alternative splicing (alternative 3′ splice site usage, 5′ splice site usage, intron retention, or mutually-exclusive exons). We generated separate datasets for the upstream and downstream intronic regions (400 bases) flanking skipped exons. A similar dataset was generated for constitutive exons. Next, the human intronic regions were aligned to orthologous intronic regions in human, mouse, rat and dog. The alternative splicing conservation enrichment score of a sequence element of length *k* (k-mer) was represented by the non-parametric χ^2^ statistic with Yates correction, computed from the two by two contingency table, T (T_11_: number of occurrences of the element perfectly conserved across alignments proximal to skipped exons; T_12_: number of occurrences of the element in human proximal to skipped exons; T_21_: number of occurrences of element perfectly conserved across alignments proximal to constitutive exons); T_22_: number of occurrences of the element in human proximal to constitutive exons). Counts in the table had to be greater than 5. The final score for each ISRE was the maximum Z-score (χ value) associated with a sequence element from the set of sequences representing the ISRE.

Analysis of enrichment/depletion of ISREs in intronic regions flanking brain and muscle-specific alternative exons

The tables below indicate the number of events obtained from supplementary material (Sugnet et al, 2006). Each event comprises of 100 bases of upstream or downstream intronic regions flanking brain/muscle-specific inclusion/skipping events from the mouse genome (mm2). The level of significance related to the confidence of the authors’ prediction of inclusion/skipping from the splicing-specific microarray. We also obtained 62,433 constitutive upstream/downstream events as a control set (Sugnet et al, 2006).

|  | Brain inclusion events | | | Brain skipping events | | |
| --- | --- | --- | --- | --- | --- | --- |
| Level of significance of events | 1.0 | 1.5 | 2.0 | 1.0 | 1.5 | 2.0 |
| Number of upstream/ downstream introns | 69 | 54 | 36 | 69 | 56 | 36 |

|  | Muscle inclusion events | | | Muscle skipping events | | |
| --- | --- | --- | --- | --- | --- | --- |
| Level of significance of events | 1.0 | 1.5 | 2.0 | 1.0 | 1.5 | 2.0 |
| Number of upstream/ downstream introns | 17 | 16 | 12 | 10 | 7 | 2 |

To determine the enrichment or depletion of ISREs in mouse introns proximal to tissue-specific alternative events, we first computed F, the frequency of an ISRE in a set of sequences of size N. For each r of 5000 random selections (R=5000), N sequences are randomly chosen from the control set and G_r_, the frequency of an ISRE in the random set R is computed. The p-value for enrichment of an ISRE is computed as (1 – E/R), where E is the number of times that (F>G_r_). The p-value for depletion is computed as (1-D/R), where D is the number of times that (G_r_>F). Due to the small numbers of muscle-skipping events, the p-values for depletion are not reliable, and hence the muscle-skipping events are excluded.

Sugnet CW, Srinivasan K, Clark TA, O'Brien G, Cline MS, Wang H, Williams A, Kulp D, Blume JE, Haussler D, Ares M Jr. (2006) Unusual intron conservation near tissue-regulated exons found by splicing microarrays. PLoS Comput Biol. Jan;2(1):e4.
